# Synthesis and Structure–Activity Relationships for the Anti-Mycobacterial Activity of 3-Phenyl-*N*-(Pyridin-2-ylmethyl)Pyrazolo[1,5-*a*]Pyrimidin-7-Amines

**DOI:** 10.3390/ph15091125

**Published:** 2022-09-08

**Authors:** Hamish S. Sutherland, Peter J. Choi, Guo-Liang Lu, Anna C. Giddens, Amy S. T. Tong, Scott G. Franzblau, Christopher B. Cooper, Brian D. Palmer, William A. Denny

**Affiliations:** 1 Auckland Cancer Society Research Centre, School of Medical Sciences, University of Auckland, Private Bag 92019, Auckland 1142, New Zealand; 2 Maurice Wilkins Centre, University of Auckland, Private Bag 92019, Auckland 1142, New Zealand; 3 Institute for Tuberculosis Research, College of Pharmacy, University of Illinois at Chicago, 833 South Wood Street, Chicago, IL 60612, USA; 4 Global Alliance for TB Drug Development, 40 Wall Street, New York, NY 10005, USA

**Keywords:** pyrazolopyrimidines, structure–activity relationships, synthesis, tuberculosis, ATP synthase

## Abstract

Pyrazolo[1,5-*a*]pyrimidines have been reported as potent inhibitors of mycobacterial ATP synthase for the treatment of *Mycobacterium tuberculosis* (*M.tb*). In this work, we report the design and synthesis of approximately 70 novel 3,5-diphenyl-*N*-(pyridin-2-ylmethyl)pyrazolo[1,5-*a*]pyrimidin-7-amines and their comprehensive structure–activity relationship studies. The most effective pyrazolo[1,5-*a*]pyrimidin-7-amine analogues contained a 3-(4-fluoro)phenyl group, together with a variety of 5-alkyl, 5-aryl and 5-heteroaryl substituents. A range of substituted 7-(2-pyridylmethylamine) derivatives were also active. Some of these compounds exhibited potent in vitro *M.tb* growth inhibition, low hERG liability and good mouse/human liver microsomal stabilities, highlighting their potential as inhibitors of *M.tb*.

## 1. Introduction

Tuberculosis (TB) is the deadliest infectious disease around the globe, claiming over a billion lives in the past two hundred years [1,2,3]. According to the World Health Organization (WHO), TB has claimed the lives of 1.3 million people, with an estimated 9.9 million new cases of TB in 2020 [4]. The current major hurdle of the global TB challenge is the fight against the drug-resistant forms of the disease [5,6]. The emergence of drug-resistant strains of *Mycobacterium tuberculosis* (*M.tb*), the causative agent of TB, is on the rise, with treatment success rates dropping for patients with multidrug-resistant (MDR) TB. The latest WHO data for drug-resistant TB suggests an estimated 465,000 new cases and 182,000 deaths resulted from MDR-TB in 2019 [7]. In the past two decades, there have also been alarming increases in *M.tb* strains with resistance to all available TB drugs, resulting in extensively and totally drug-resistant/incurable tuberculosis [8,9,10,11]. The development of novel TB agents to treat these resistant strains of *M.tb* is urgently needed.

**Figure 1:**
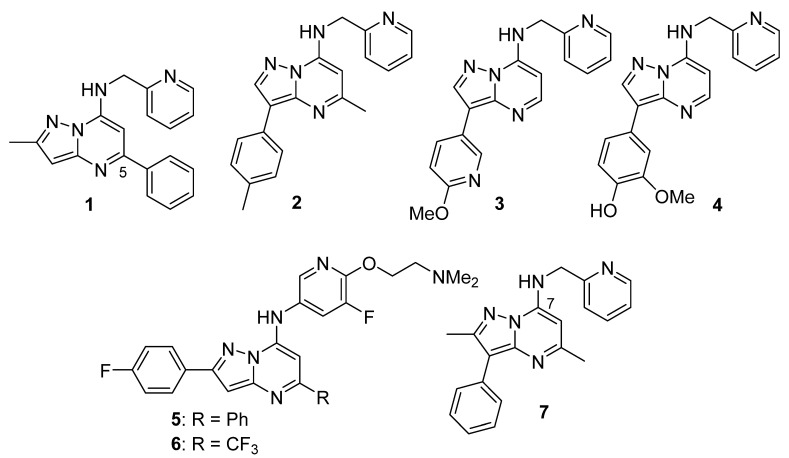
Pyrazolo[1,5-*a*]pyrimidine-based drug candidates.

Pyrazolo[1,5-*a*]pyrimidines have been reported as potential drugs in a number of different areas (Figure 1); as VEGF/src inhibitors (e.g., **1**) [12], as apoptosis inducers (e.g., **2**) [13], for treatment of Duchenne muscular dystrophy (e.g., **3**) [14] and as cyclin-dependent kinase inhibitors (e.g., **4**) [15]. In a recent paper, Tantry et al. [16,17] also discuss 2-phenyl-5-substituted pyrazolo[1,5-*a*]pyrimidines as inhibitors of mycobacterial ATP synthase. Modelling studies suggest the latter compounds bind between the Atp-a and Atp-c (chain-B) subunits of the enzyme, with the pendant 5-phenyl ring occupying the hydrophobic space between the two Atp-c subunits. Example compounds **5** and **6** were modest inhibitors of both depletion of ATP (using an *M. smegmatis* inverted membrane vesicle assay to measure ATP inhibition via the oxidative phosphorylation process) and of inhibition of *M.tb* bacteria in culture, and were active *in vivo* in an acute mouse model of tuberculosis. Chibale et al. [18] also explored SAR for 7-substituted pyrazolo[1,5-*a*]pyrimidines as *M.tb* drugs (e.g., **7**) and showed that the optimal C-7 side chain was 2-pyridinemethanamine.

In the present paper, we extend the latter work with the synthesis of a series of novel 3,5-diphenyl-*N*-(pyridin-2-ylmethyl)pyrazolo[1,5-*a*]pyrimidin-7-amines with substituted 3- and/or 5-phenyl rings, exploring relationships between substituent patterns, overall physicochemical properties and their antimycobacterial activity (inhibition of *M.tb* in culture) (Table 1, Table 2 and Table 3).

## 2. Results

**Scheme 1:**
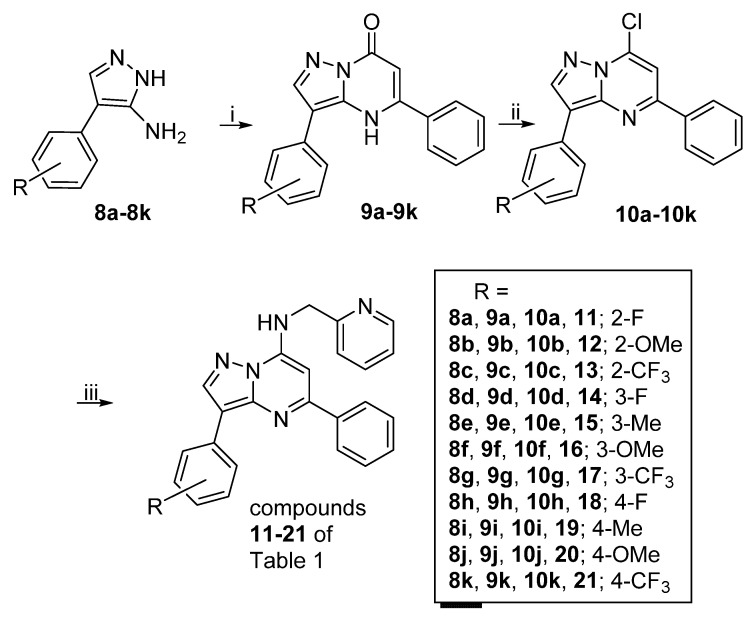
Preparation of compounds **11–21** of Table 1. Reagents and conditions: (i) PhCOCH_2_CO_2_Et, AcOH, 110 °C; (ii) POCl_3_, Me_4_N^+^Cl^−^, reflux; (iii) 2-pyridinemethanamine, DIPEA, *^i^*PrOH, reflux, 16 h.

Compounds **11–21** of Table 1 (Scheme 1) were prepared from commercially substituted 4-phenyl-1*H*-pyrazol-5-amines (**8a–8k**) by condensation with 3-oxo-3-phenylpropanoate to give the 3-phenyl-substituted 3,5-diphenylpyrazolo[1,5-*a*]pyrimidin-7(4*H*)-ones (**9a–9k**). Pyrimidinones (**9a–9k**) were converted to 7-chlorides (**10a–10k**) using POCl_3_ and tetramethylammonium chloride, 7-chlorides (**10a–10k**) were subsequently reacted with 2-pyridinemethanamine to give the compounds **11–21** of Table 1. 

**Scheme 2:**
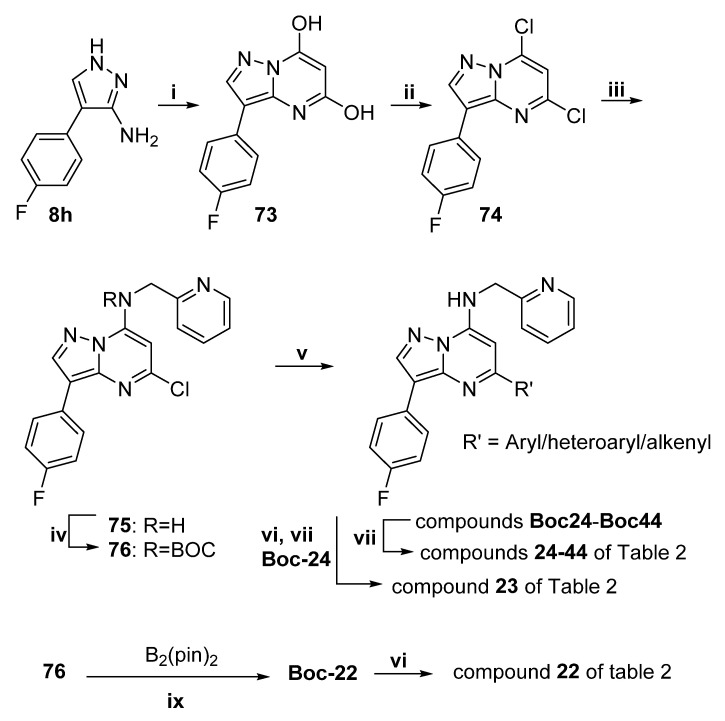
Preparation of compounds **22–44** of Table 2. Reagents and conditions: (i) EtO_2_CCH_2_CO_2_Et, NaOEt, EtOH, reflux; (ii) Me_4_N^+^Cl^−^/POCl_3_, reflux; (iii) 2-pyridinemethanamine, DIPEA, DCM, 0–20 °C; (iv) Boc_2_O, cat. DMAP, Et_3_N, DCM, r.t.; (v) ArB(OH)_2_ or HetB(OH)_2_, Pd(PPh_3_)_4_, Na_2_CO_3_, toluene, reflux; (vi) 10% Pd/C, 50 psi H_2_, MeOH, r.t.; (vii) 1:1 TFA/DCM, reflux; (ix) KOAc, PdCl_2_(dppf), DMSO, 80 °C.

Compounds **22–44** of Table 2 (Scheme 2) were prepared from commercial 4-(4-fluorophenyl)-1*H*-pyrazol-5-amine (**8h**) by reacting it with diethyl malonate/sodium ethoxide to give the diol (**73**), which was converted to the dichloride (**74**). The chloride at the 7 position was selectively displaced with 2-pyridinemethanamine to give **75**, BOC-protection of the amine gave **76**, which subsequently underwent Suzuki coupling with various aryl and heteroaryl boronic acids to give **Boc24–Boc44**. The Suzuki coupling was unsuccessful under a variety of conditions when **75** was used, and this necessitated the use of the Boc-protected derivative **76**.

**Boc24** was hydrogenated using palladium on carbon to give **Boc23**. An attempt to convert **76** to a boronate ester was unsuccessful, only the product (**Boc22**) arising from reduction was isolated.

Deprotection of the Boc-protected intermediates (**Boc22–Boc44**) using trifluoroacetic acid in DCM at reflux gave the compounds **22–44** of Table 2. 

**Scheme 3:**
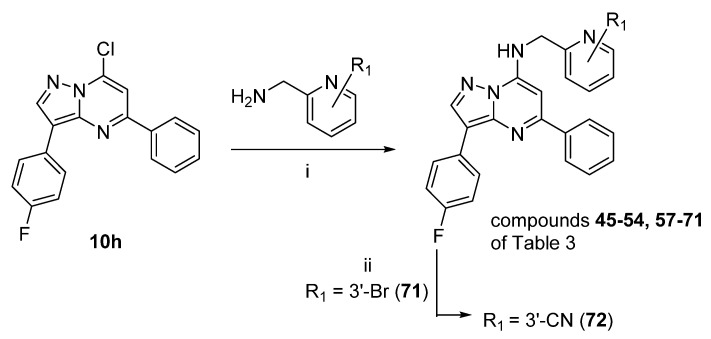
Preparation of compounds **45–54, 57–71** of Table 3. Reagents and conditions: (i) DIPEA, *^i^*PrOH, reflux, 3–15h; (ii) Zn(CN)_2_, Pd(PPh_3_)_4_, DMF, 115 °C.

Compounds **45–71** of Table 3 were prepared as shown in Scheme 3, from **10h** and the appropriate substituted pyridine-2-ylmethanamines. The majority of the pyridine-2-ylmethanamines were available commercially; the synthesis of pyridylmethanamines used in the synthesis of **57** and **58** is described in the supplementary information.

The 3′-bromide (**71**) underwent a palladium-mediated cyanation to give the corresponding 3′-cyano derivative (**72**).

**Scheme 4:**
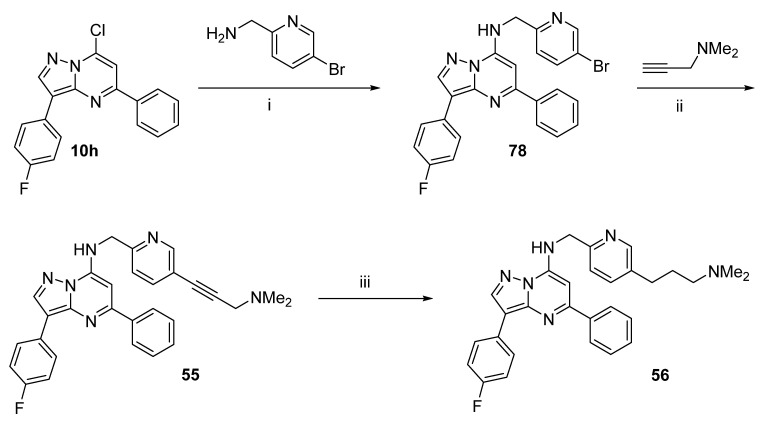
Preparation of compounds **55** and **56** of Table 3. Reagents and conditions: (i) DIPEA, iPrOH, reflux, 19 h; (ii) PdCl_2_(PPh_3_)_2_, CuI, Et_3_N, DMF, 65 °C; (iii) 5% Pd/C, EtOH, H_2_ (60 psi).

Compounds **55** and **56** of Table 3 were synthesised from **10h**, which underwent a displacement reaction with (5-bromopyridin-2-yl)methanamine to give **78** (Scheme 4). Sonogashira coupling of **78** with dimethylprop-2-ynamine gave **55**, and the alkyne was subsequently hydrogenated to give **56**.

## 3. Results and Discussion

The compounds of Table 1, Table 2 and Table 3 were tested for their ability to inhibit the growth of *Mycobacterium tuberculosis* (strain H37Rv) when cultured under either aerobic (MABA) [19] or low-oxygen (LORA) [20] conditions, by determining the minimum inhibitory concentrations (MIC_90_; µg/mL) needed to reduce growth by 90%. The compounds were also assessed for their ability to inhibit the growth of mammalian cells (VERO green monkey kidney cells) by determining IC_50_ values [21]. The majority of the compounds were non-toxic in this assay (IC_50_ of >32 µg/mL).

### Overall Lipophilicity Structure–Activity Relationships

It has been consistently observed across many classes of tuberculosis inhibitors that MIC potency usually increases for more lipophilic compounds; a phenomenon that has been attributed [22,23] to the usually lipophilic cell wall of *Mycobacterium tuberculosis* restricting the passive diffusion of large hydrophilic compounds. While no statistically significant correlation was seen across the whole set of quite diverse compounds in Table 1, Table 2 and Table 3, there was a modest correlation of higher MIC potency with increasing lipophilicity (Equation (1)) for the small but more tightly-defined set of eleven 5-(substituted phenyl) compounds **28–38** of Table 2,

Log(IC_50_MABA) = −0.22(±0.11)clogP + 1.65(±0.62) n = 11 R = 0.55 F = 3.96(1)

Table 1 records MABA and LORA data (MIC_90_, µg/mL) for a series of compounds **11–21** bearing a range of differing substituents off the 3-phenyl ring. The 4-F and 4-OMe substituted compounds were clearly the most potent in both the MABA (>5.5-fold) and LORA (>7.5-fold) assays, possibly by blocking metabolism [24], and the 4-F substitution in this ring was thus employed in all the later compounds in Table 2 and Table 3. The lack of activity for compounds with an *ortho* substituent could be the result of an unfavourable change in torsion angle between the pendant aryl group and the pyrazolopyrimidine core due to increased steric hindrance. However, electronic or steric arguments cannot be used to rationalise the difference in activity between active (**18**, **20**) and inactive (**19**, **21**) *para* substituted compounds.

With the 3-phenyl ring substituent fixed as 4-F, a more extensive SAR study was carried out on the 5-substituent (Table 2). The compounds in Table 2 explore structure–activity relationships for pyrazolopyrimidine 5-substituents, from hydrogen (compound **22**) and simple alkyls or alkenyls (compounds **23–27**), and for a series of additional substituted phenyl (compounds **28–38**) and other heteroaromatic substituents (compounds **39–44**). Compounds **22–27** show that those with no 5-substituent, or with a series of linear saturated and unsaturated alkyl/alkenyl groups, retain good in vitro anti-mycobacterial activities (MABA and LORA MICs) from 0.2 to 1.5 µg/mL. The two 2-(substituted phenyl) analogues (**28**, **29**) suggested (not unexpectedly) that only very small substituents (F) were permitted at this site, and bulky substituents at this position may induce an unfavourable change in torsion angle between the pendant 5-aryl group and the pyrazolopyrimidine core. The set of 4-(substituted phenyl) compounds (**32–38**), despite covering substituents with a wide variation in bulk, lipophilic and electronic properties, showed similar and quite potent MICs (from 0.2–3.8 ug/mL), suggesting significant bulk tolerance at this position. Compounds **39–44** carry aromatic rings other than phenyl at the 5-position, resulting in some compounds of generally lower lipophilicity (clogP below about 4) while retaining activity, with the exception of **40**.

Going forward, it was therefore decided to retain a 3-(4-fluoro)phenyl group and an unsubstituted 5-phenyl group for the subsequent SAR study of the 7-(2-pyridylmethylamine) group structure–activity relationships (Table 3; compounds **45–72**). In this series, small substituents with varied electronic and lipophilicity characteristics were used in each available position. Compounds **45–48** showed that neutral groups with varying physicochemical properties at the 6′-position were not favoured, whereas powerful electron donors resulted in active compounds (**49–51**).

The compounds of Table 3 explore the SAR for substituents on the 7-(2-pyridylmethylamine) benzyl group, while holding the 3- and 5-substituents constant as 4-fluorophenyl and phenyl, respectively. Compounds **45–51** explore substituents on the 6′-position, *ortho* to the pyridine nitrogen. Only compounds **49–51**, with a variety of aliphatic amine substituents, were active, with MICs of around 1 µg/mL. In contrast, a series of compounds (**52–59)** bearing 5′-substituents of different electronic, lipophilic and steric properties all showed modest to good activity (MICs of 1–7 µg/mL). 

A larger group of 4′-substituted analogues (compounds **60–68**) showed clearer structure–activity relationships. Those (**60**, **61**) bearing electron-withdrawing groups were inactive, whereas those with electron-donating groups were active, with the best (**65–67, 69**; averaged MABA/LORA MICs of 0.40 and 0.88 µg/mL, respectively) all bearing the strongest, amine-based, electron donors. Of particular note was the 4′-*N*-morpholine derivative (**66**), which showed excellent inhibitory data (MABA/LORA MICs of 0.06 and 0.98 µg/mL, respectively), in conjunction with low toxicity towards mammalian cells (VERO > 32 µg/mL).

Finally, a small group of 3′-substituted analogues (compounds **69–72**) showed a similar dependence of activity on substituent electronic properties, with electron-donating substituents providing more potent anti-mycobacterial inhibition, while those with halogen or electron-withdrawing substituents at the 3′-position were inactive.

A representative subset of the more active compounds from the cell culture assays were evaluated for their microsomal stability and hERG inhibitory properties, and the results are provided in Table 4.

From the data in Table 4, compounds **18**, **20**, **25**, **26**, **27**, **28**, **34**, **38**, **39**, **41**, **42**, **44** were the least stable, with T½ values of <85 min. An almost equal number of analogues (compounds **30**, **35**, **36**, **52–55**, **62**, **63** and **68**) were considerably more stable (T½ values from 109 to >145 min) and were also much more lipophilic (average clogP 5.88 compared to 4.39). Apart from compounds **28**, **39** and **68**, there was very little inhibition of the hERG potassium ion channel observed by the pyrazolopyrimidines, even at 1 µM. While contributing factors for drugs to express significant hERG inhibition are high logP, high basicity and drug flexibility [25], the above three compounds do not stand out from the others in these properties.

## 4. Material and Methods

### 4.1. General Information

Final products were analysed by reverse-phase HPLC (Alltima C18 5 µm column, 15 × 3.2 mm; Alltech Associated, Inc., Deerfield, IL, USA) using an Agilent HP1100 equipped with a diode-array detector. It was run using mobile phases with 80% CH_3_CN/20% H_2_O (*v*/*v*) in 45 mM NH_4_HCO_2_ at pH 3.5 and 0.5 mL/min. The purity level was determined by monitoring at 330 ± 50 nm and was ≥95% for all final products. NMR spectra were obtained on a Bruker Avance 400 spectrometer at 400 MHz for ^1^H and ^13^C. Low-resolution mass spectra (LRMS), using atmospheric pressure chemical ionisation (APCI), were measured on a ThermoFinnigan Surveyor MSQ mass spectrometer, connected to a Gilson autosampler. High resolution mass spectra (HRMS) were obtained using an Agilent G6530B Q-TOF spectrometer and are reported as M + H. Melting points were determined on an Electrothermal 9100 melting point apparatus. Copies of the ^1^H and ^13^C Spectra for compounds that progressed to advanced testing in Table 4 are available in the Supplementary Materials.

Preparation of compounds **11**–**21** of Table 1.


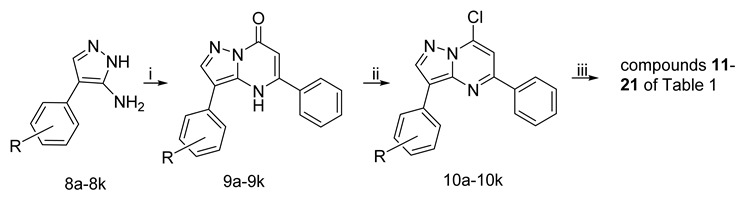


### 4.2. Synthesis of the 3-Aryl-5-Phenylpyrazolo[1,5-a]Pyrimidin-7(4H)-One (**9a**–**9k**) Derivatives

3-(2-Fluorophenyl)-5-phenylpyrazolo[1,5-*a*]pyrimidin-7(4*H*)-one (**9a**) 

A solution of 4-(2-fluorophenyl)-1*H*-pyrazol-5-amine (**8a**, 0.412 g, 2.33 mmol) and ethyl 3-oxo-3-phenylpropanoate (0.625 g, 3.25 mmol) in acetic acid (10 mL) was refluxed at 100 °C for 16 h. The mixture was cooled then diluted with diethyl ether (20 mL), and the resulting solid was filtered, washed with diethyl ether and dried to give **9a** (0.435 g, 61%) as a white solid. ^1^H NMR (DMSO-d_6_) δ 12.26 (bs, 1H), 8.12 (s, 1H), 7.79 (bd, *J* = 6.0 Hz, 2H), 7.63 (t, *J* = 7.4 Hz, 1H), 7.55–7.61 (m, 3H), 7.38–7.45 (m, 1H), 7.27–7.36 (m, 2H), 6.08 (s, 1H). LRMS [M + H] = 306.2.

3-(2-Methoxyphenyl)-5-phenylpyrazolo[1,5-*a*]pyrimidin-7(4*H*)-one (**9b**)

Synthesised from **8b** (2.997 g, 15.8 mmol) using the general procedure to give **9b** (3.075 g, 61%) as a white solid. ^1^H NMR (DMSO-d_6_) δ 11.85 (s, 1H), 8.05 (s, 1H), 7.80 (dd, *J* = 7.8, 2.0 Hz, 1H), 7.53–7.59 (m, 3H), 7.45 (d, *J* = 6.4 Hz, 1H), 7.35 (td, *J* = 7.8, 1.6 Hz, 1H), 7.11 (dd, *J* = 8.3, 0.6 Hz, 1H), 7.03 (td, *J* = 7.4, 1.0 Hz, 1H), 6.03 (s, 1H), 3.90 (s, 3H). LRMS [M + H] = 318.1.

5-Phenyl-3-(2-(trifluoromethyl)phenyl)pyrazolo[1,5-*a*]pyrimidin-7(4*H*)-one (**9c**)

Synthesised from **8c** (0.391 g, 1.72 mmol) using the general procedure to give **9c** (0.335 g, 55%) as a white solid. ^1^H NMR (DMSO-d_6_) δ 7.89 (s, 1H), 7.85 (d, *J* = 7.8 Hz, 1H), 7.73 (t, *J* = 7.2 Hz, 1H), 7.69 (d, *J* = 6.6 Hz, 2H), 7.50–7.66 (m, 5H), 6.01 (s, 1H), one H exchanged. LRMS [M + H] = 356.1.

3-(3-Fluorophenyl)-5-phenylpyrazolo[1,5-*a*]pyrimidin-7(4*H*)-one (**9d**)

Synthesised from **8d** (1.00 g, 5.64 mmol) using the general procedure to give **9d** (1.342 g, 78%) as a white solid. ^1^H NMR (DMSO-d_6_) δ 12.26 (s, 1H), 8.25 (s, 1H), 7.84 (bs, 2H), 7.42–7.61 (m, 6H), 7.10–7.15 (m, 1H), 6.05 (s, 1H). LRMS [M + H] = 306.2.

5-Phenyl-3-(*m*-tolyl)pyrazolo[1,5-*a*]pyrimidin-7(4*H*)-one (**9e**)

Synthesised from **8e** (1.346 g, 7.77 mmol) using the general procedure to give **9e** (1.914 g, 82%) as a white solid. ^1^H NMR (DMSO-d_6_) δ 12.22 (s, 1H), 8.17 (s, 1H), 7.82 (bd, *J* = 5.5 Hz, 2H), 7.54–7.61 (m, 3H), 7.44–7.50 (m, 2H), 7.33 (t, *J* = 7.6 Hz, 1H), 7.13 (t, *J* = 7.5 Hz, 1H), 5.66 (s, 1H), 2.38 (s, 3H). LRMS [M + H] = 302.2.

3-(3-Methoxyphenyl)-5-phenylpyrazolo[1,5-*a*]pyrimidin-7(4*H*)-one (**9f**)

Synthesised from **8f** (1.659 g, 8.77 mmol) using the general procedure to give **9f** (1.729 g, 62%) as a white solid. ^1^H NMR (DMSO-d_6_) δ 12.24 (s, 1H), 8.20 (s, 1H), 7.82 (bd, *J* = 5.6 Hz, 2H), 7.53–7.60 (m, 3H), 7.36 (t, *J* = 8.0 Hz, 1H), 7.22 (bs, 2H), 6.88 (d, *J* = 7.2 Hz, 1H), 6.03 (s, 1H), 3.84 (s, 3H). LRMS [M + H] = 318.1.

5-Phenyl-3-(3-(trifluoromethyl)phenyl)pyrazolo[1,5-*a*]pyrimidin-7(4*H*)-one (**9g**)

Synthesised from **8g** (1.394 g, 6.14 mmol) using the general procedure to give **9g** (1.527 g, 70%) as a white solid. ^1^H NMR (DMSO-d_6_) δ 12.36 (s, 1H), 8.30 (s, 1H), 7.96 (bs, 2H), 7.78 (bs, 2H), 7.45–7.90 (m, 5H), 6.07 (s, 1H). LRMS [M + H] = 356.1.

3-(4-Fluorophenyl)-5-phenylpyrazolo[1,5-*a*]pyrimidin-7(4*H*)-one (**9h**)

Synthesised from **8h** (2.835 g, 16.0 mmol) using the general procedure to give **9h** (4.202 g, 86%) as a cream solid. ^1^H NMR (DMSO-d_6_) δ 12.23 (bs, 1H), 8.17 (bs, 1H), 7.83 (bd, *J* = 4.9 Hz, 2H), 7.69 (bs, 2H), 7.52–7.62 (m, 3H), 7.28 (tt, *J* = 9.0, 2.1 Hz, 2H), 6.03 (bs, 1H). LRMS [M + H] = 306.2.

5-Phenyl-3-(*p*-tolyl)pyrazolo[1,5-*a*]pyrimidin-7(4*H*)-one (**9i**)

Synthesised from **8i** (1.00 g, 5.78 mmol) using the general procedure to give **9i** (1.434 g, 82%) as a tan solid. ^1^H NMR (DMSO-d_6_) δ 12.18 (s, 1H), 8.15 (s, 1H), 7.82 (bd, *J* = 4.2 Hz, 2H), 7.54–7.60 (m, 5H), 7.27 (d, *J* = 7.9 Hz, 2H), 6.02 (s, 1H), 2.34 (s, 3H). LRMS [M + H] = 302.2.

3-(4-Methoxyphenyl)-5-phenylpyrazolo[1,5-*a*]pyrimidin-7(4*H*)-one (**9j**)

Synthesised from **8j** (1.252 g, 6.62 mmol) using the general procedure to give crude **9j**. This was extracted with EtOAc, dried and evaporated to give the crude pyrimidinone, which was used directly in the subsequent step.

5-Phenyl-3-(4-(trifluoromethyl)phenyl)pyrazolo[1,5-*a*]pyrimidin-7(4*H*)-one (**9k**)

Synthesised from **8k** (0.934 g, 4.11 mmol) using the general procedure to give **9k** (0.923 g, 63%) as a white solid. ^1^H NMR (DMSO-d_6_) δ 12.32 (s, 1H), 8.30 (s, 1H), 7.82–7.95 (m, 4H), 7.79 (apd, *J* = 8.1 Hz, 2H), 7.52–7.62 (m, 3H), 6.09 (s, 1H). LRMS [M + H] = 356.1.

Synthesis of the 7-chloro-3-(aryl)-5-phenylpyrazolo[1,5-*a*]pyrimidine derivatives (**10a**–**10k**).

7-Chloro-3-(2-fluorophenyl)-5-phenylpyrazolo[1,5-*a*]pyrimidine (**10a**)

A slurry of 3-(2-fluorophenyl)-5-phenylpyrazolo[1,5-a]pyrimidin-7(4H)-one (**9a**, 0.438 g, 1.43 mmol) and tetramethylammonium chloride (0.235 g, 2.15 mmol) in POCl_3_ (10 mL) was refluxed for 0.5 h. Excess POCl_3_ was removed in *vacuo* and the residue was slurried with DCM (50 mL) and then poured onto ice. The aqueous layer was extracted with DCM (2 × 25 mL), the organic fractions were combined, dried (MgSO_4_) and evaporated. Chromatography on neutral alumina using DCM as an eluent gave **10a** (0.424 g, 92%) as a yellow solid. ^1^H NMR (CDCl_3_) δ 8.71 (d, *J* = 3.4 Hz, 1H), 8.64 (td, *J* = 6.4, 3.7 Hz, 1H), 8.14–8.18 (m, 2H), 7.53–7.59 (m, 3H), 7.52 (s, 1H), 7.28–7.34 (m, 2H), 7.17–7.23 (m, 1H). LRMS [M + H] = 324.1, 326.1.

7-Chloro-3-(2-methoxyphenyl)-5-phenylpyrazolo[1,5-*a*]pyrimidine (**10b**)

Synthesised from **9b** (1.00 g, 3.15 mmol) using the general procedure to give **10b** (1.043 g, 99%) as a yellow solid. ^1^H NMR (CDCl_3_) δ 8.82 (s, 1H), 8.47 (dd, *J* = 7.7, 1.7 Hz, 1H), 8.14–8.17 (m, 2H), 7.51–7.56 (m, 3H), 7.47 (s, 1H), 7.32 (ddd, *J* = 9.2, 7.4, 1.8 Hz, 1H), 7.14 (td, *J* = 7.6, 1.1 Hz, 1H), 7.05 (dd, *J* = 8.3, 0.9 Hz, 1H), 3.97 (s, 3H). LRMS [M + H] = 336.1, 338.1.

7-Chloro-5-phenyl-3-(2-(trifluoromethyl)phenyl)pyrazolo[1,5-*a*]pyrimidine (**10c**)

Synthesised from **9c** (0.312 g, 0.878 mmol) using the general procedure to give **10c** (0.323 g, 98%) as a yellow solid. ^1^H NMR (CDCl_3_) δ 8.37 (d, *J* = 0.8 Hz, 1H), 8.04–8.09 (m, 2H), 7.83 (d, *J* = 8.0 Hz, 1H), 7.80 (d, *J* = 7.8 Hz, 1H), 7.66 (t, *J* = 7.1 Hz, 1H), 7.47–7.53 (m, 5H). LRMS [M + H] = 374.1, 376.1.

7-Chloro-3-(3-fluorophenyl)-5-phenylpyrazolo[1,5-*a*]pyrimidine (**10d**)

Synthesised from **9d** (1.00 g, 3.28 mmol) using the general procedure to give **10d** (1.01 g, 95%) as a yellow solid. ^1^H NMR (CDCl_3_) δ 8.54 (s, 1H), 8.15–8.20 (m, 2H), 7.98 (ddd, *J* = 10.6, 2.5, 1.6 Hz, 1H), 7.90 (dt, *J* = 7.8, 1.4 Hz, 1H), 7.53–7.60 (m, 3H), 7.51 (s, 1H), 7.41–7.47 (m, 1H), 7.00 (tdd, *J* = 8.5, 2.6, 0.9 Hz, 1H). LRMS [M + H] = 324.1, 326.1.

7-Chloro-5-phenyl-3-(*m*-tolyl)pyrazolo[1,5-*a*]pyrimidine (**10e**)

Synthesised from **9e** (1.292 g, 4.29 mmol) using the general procedure to give **10e** (1.275 g, 93%) as a yellow solid. ^1^H NMR (CDCl_3_) δ 8.54 (s, 1H), 8.15–8.20 (m, 2H), 8.01 (bd, *J* = 7.8 Hz, 1H), 7.94 (bs, 1H) 7.51–7.59 (m, 3H), 7.49 (s, 1H), 7.39 (t, *J* = 7.7 Hz, 1H), 7.14 (bd, *J* = 7.5 Hz, 1H), 2.47 (s, 3H). LRMS [M + H] = 320.2, 322.2.

7-Chloro-3-(3-methoxyphenyl)-5-phenylpyrazolo[1,5-*a*]pyrimidine (**10f**)

Synthesised from **9f** (1.00 g, 3.15 mmol) using the general procedure to give **10f** (1.05 g, 99%) as a yellow solid. ^1^H NMR (CDCl_3_) δ 8.54 (s, 1H), 8.14–8.19 (m, 2H), 7.85 (dd, *J* = 2.5, 1.6 Hz, 1H), 7.71 (ddd, *J* = 8.6, 2.4, 1.0 Hz, 1H), 7.51–7.58 (m, 3H), 7.48 (s, 1H), 7.40 (t, *J* = 8.0 Hz, 1H), 6.87 (ddd, *J* = 8.3, 2.6, 0.8 Hz, 1H), 3.93 (s, 3H). LRMS [M + H] = 336.1, 338.1.

7-Chloro-5-phenyl-3-(3-(trifluoromethyl)phenyl)pyrazolo[1,5-*a*]pyrimidine (**10g**)

Synthesised from **9g** (1.00 g, 2.81 mmol) using the general procedure to give **10g** (1.028 g, 98%) as a yellow solid. ^1^H NMR (CDCl_3_) δ 8.57 (s, 1H), 8.54 (s, 1H), 8.31 (d, *J* = 7.6 Hz, 1H), 8.14–8.19 (m, 2H), 7.52–7.62 (m, 6H). LRMS [M + H] = 374.1, 376.1.

7-Chloro-3-(4-fluorophenyl)-5-phenylpyrazolo[1,5-*a*]pyrimidine (**10h**)

Synthesised from **9h** (4.187 g, 13.7 mmol) using the general procedure to give **10h** (4.398 g, 99%) as a yellow solid. ^1^H NMR (CDCl_3_) δ 8.51 (s, 1H), 8.11–8.18 (m, 4H), 7.52–7.59 (m, 3H), 7.49 (s, 1H), 7.19 (ap t, *J* = 8.8 Hz, 2H). LRMS [M + H] = 324.1, 326.1.

7-Chloro-5-phenyl-3-(*p*-tolyl)pyrazolo[1,5-*a*]pyrimidine (**10i**)

Synthesised from **9i** (1.024 g, 3.40 mmol) using the general procedure to give **10i** (1.035 g, 95%) as a yellow solid. ^1^H NMR (CDCl_3_) δ 8.52 (s, 1H), 8.18 (ap dd, *J* = 8.0, 1.6 Hz, 2H), 8.05 (d, *J* = 8.2 Hz, 2H), 7.51–7.58 (m, 3H), 7.47 (s, 1H), 7.31 (d, *J* = 7.9 Hz, 2H), 2.42 (s, 3H). LRMS [M + H] = 320.2, 322.2.

7-Chloro-3-(4-methoxyphenyl)-5-phenylpyrazolo[1,5-*a*]pyrimidine (**10j**)

Synthesised using the crude intermediate **9j** using the general procedure to give **10j** (0.863 g, 40%, two steps) as a yellow solid. ^1^H NMR (CDCl_3_) δ 8.48 (s, 1H), 8.16 (dd, *J* = 8.0, 2.1 Hz, 2H), 8.09 (d, *J* = 8.9 Hz, 2H), 7.52–7.57 (m, 3H), 7.46 (s, 1H), 7.05 (d, *J* = 8.9 Hz, 2H), 3.88 (s, 3H). LRMS [M + H] = 336.1, 338.1.

7-Chloro-5-phenyl-3-(4-(trifluoromethyl)phenyl)pyrazolo[1,5-*a*]pyrimidine (**10k**)

Synthesised from **9k** (0.810 g, 2.28 mmol) using the general procedure to give **10k** (0.836 g, 98%) as a yellow solid. ^1^H NMR (CDCl_3_) δ 8.58 (s, 1H), 8.29 (d, *J* = 8.1 Hz, 2H), 8.14–8.19 (m, 2H), 7.74 (d, *J* = 8.2 Hz, 2H), 7.54–7.60 (m, 3H), 7.53 (s, 1H). LRMS [M + H] = 374.1, 376.1.

### 4.3. Pyrazolo[1,5-a]Pyrimidine-7-Amines (Compounds **11**–**21**)

3-(2-Fluorophenyl)-5-phenyl-*N*-(pyridin-2-ylmethyl)pyrazolo[1,5-*a*]pyrimidin-7-amine (**11**)

A solution of **10a** (0.194 g, 0.599 mmol), pyridin-2-ylmethanamine (77 mg, 0.71 mmol) and diisopropylethylamine (0.15 mL, 0.90 mmol) in isopropanol (20 mL) was refluxed for 16 h. The solvent was removed in *vacuo* and the residue was partitioned between water, EtOAc and saturated NaHCO_3_ (until basic). The aqueous layer was extracted with EtOAc and the organic fractions were dried and evaporated to give the crude product, which was recrystallised from DCM/heptane by evaporation of DCM to give **11** (0.206 g, 87%) as pale yellow microcrystals. Purity (HPLC) 99.5%; mp 176–178 °C. ^1^H NMR (CDCl_3_) δ 8.84 (td, *J* = 7.9, 1.7 Hz, 1H), 8.69 (ddd, *J* = 4.9, 1.6, 0.9 Hz, 1H), 8.60 (d, *J* = 3.7 Hz, 1H), 8.13 (ap dd, *J* = 8.3, 1.6 Hz, 2H), 7.73 (td, *J* = 7.7, 1.8 Hz, 1H), 7.60 (t, *J* = 5.3 Hz, 1H), 7.44–7.54 (m, 3H), 7.39 (d, *J* = 7.8 Hz, 1H), 7.25–7.32 (m, 2H), 7.13–7.23 (m, 2H), 6.51 (s, 1H), 4.84 (d, *J* = 5.4 Hz, 2H). HRMS calcd. for C_24_H_19_FN_5_ (M + H^+^) *m/z* 396.1624, found 396.1619.

3-(2-Methoxyphenyl)-5-phenyl-*N*-(pyridin-2-ylmethyl)pyrazolo[1,5-*a*]pyrimidin-7-amine (**12**)

Synthesised from **10b** (0.213 g, 0.600 mmol) using the general procedure to give **12** (0.194 g, 79%) as pale yellow microcrystals. Purity (HPLC) 99.5%; mp 169–171 °C. ^1^H NMR (CDCl_3_) δ 8.73 (s, 1H), 8.67–8.71 (m, 2H), 8.12 (ap dd, *J* = 8.3, 1.7 Hz, 2H), 7.72 (td, *J* = 7.7, 1.8 Hz, 1H), 7.42–7.54 (m, 4H), 7.39 (d, *J* = 7.8 Hz, 1H), 7.22–7.29 (m, 2H), 7.14 (td, *J* = 7.6, 1.2 Hz, 1H), 7.02 (dd, *J* = 8.2, 1.0 Hz, 1H), 6.48 (s, 1H), 4.84 (d, *J* = 5.4 Hz, 2H), 3.95 (s, 3H). HRMS calcd. for C_25_H_22_N_5_O (M + H^+^) *m/z* 408.1824, found 408.1816.

5-Phenyl-*N*-(pyridin-2-ylmethyl)-3-(2-(trifluoromethyl)phenyl)pyrazolo[1,5-*a*]pyrimidin-7-amine (**13**)

Synthesised from **10c** (0.112 g, 0.300 mmol) using the general procedure to give **13** (0.107 g, 80%) as cream microcrystals. Purity (HPLC) 98.5%; mp 166–168 °C. ^1^H NMR (CDCl_3_) δ 8.69 (d, *J* = 4.3 Hz, 1H), 8.26 (d, *J* = 1.0 Hz, 1H), 8.05 (ap dd, *J* = 7.5, 1.0 Hz, 2H), 7.99 (d, *J* = 7.4 Hz, 1H), 7.79 (d, *J* = 7.6 Hz, 1H), 7.74 (td, *J* = 7.7, 1.8 Hz, 1H), 7.63 (t, *J* = 7.6 Hz, 1H), 7.60 (t, *J* = 5.2 Hz, 1H), 7.39–7.47 (m, 5H), 7.29 (dd, *J* = 7.4, 5.5 Hz, 1H), 4.84 (d, *J* = 5.3 Hz, 2H). HRMS calcd. for C_25_H_19_F_3_N_5_ (M + H^+^) *m/z* 446.1593, found 446.1584.

3-(3-Fluorophenyl)-5-phenyl-*N*-(pyridin-2-ylmethyl)pyrazolo[1,5-*a*]pyrimidin-7-amine (**14**)

Synthesised from **10d** (0.194 g, 0.597 mmol) using the general procedure to give **14** (0.163 g, 69%) as yellow microcrystals. Purity (HPLC) 99.9%; mp 170–172 °C. ^1^H NMR (CDCl_3_) δ 8.69 (d, *J* = 4.3 Hz, 1H), 8.40 (s, 1H), 8.15 (ap dd, *J* = 7.9, 1.7 Hz, 2H), 8.05 (ddd, *J* = 11.0, 2.5, 1.6 Hz, 1H), 7.95 (dd, *J* = 7.8, 1.0 Hz, 1H), 7.73 (td, *J* = 7.7, 1.8 Hz, 1H), 7.59 (t, *J* = 5.1 Hz, 1H), 7.45–7.55 (m, 3H), 7.37–7.42 (m, 2H), 7.29 (dd, *J* = 7.5, 5.5 Hz, 1H), 6.92 (tdd, *J* = 6.4, 2.6, 0.8 Hz, 1H), 6.51 (s, 1H), 4.83 (d, *J* = 5.3 Hz, 2H). HRMS calcd. for C_24_H_19_FN_5_ (M + H^+^) *m/z* 396.1624, found 396.1632.

5-Phenyl-*N*-(pyridin-2-ylmethyl)-3-(*m*-tolyl)pyrazolo[1,5-*a*]pyrimidin-7-amine (**15**)

Synthesised from **10e** (0.192 g, 0.600 mmol) using the general procedure to give **15** (0.209 g, 89%) as yellow microcrystals. Purity (HPLC) 99.0%; mp 172–174 °C. ^1^H NMR (CDCl_3_) δ 8.68 (dt, *J* = 2.4, 0.9 Hz, 1H), 8.40 (s, 1H), 8.15 (ap d, *J* = 6.7 Hz, 2H), 8.06 (d, *J* = 7.8 Hz, 1H), 8.00 (s, 1H), 7.73 (td, *J* = 7.7, 1.8 Hz, 1H), 7.44–7.56 (m, 4H), 7.39 (d, *J* = 8.5 Hz, 1H), 7.35 (d, *J* = 7.7 Hz, 1H), 7.28 (dd, *J* = 7.4, 5.7 Hz, 1H), 7.06 (d, *J* = 7.5 Hz, 1H), 6.48 (s, 1H), 4.83 (d, *J* = 5.4 Hz, 2H), 2.45 (s, 3H). HRMS calcd. for C_25_H_22_N_5_ (M + H^+^) *m/z* 392.1870, found 392.1859.

3-(3-Methoxyphenyl)-5-phenyl-*N*-(pyridin-2-ylmethyl)pyrazolo[1,5-*a*]pyrimidin-7-amine (**16**)

Synthesised from **10f** (0.201 g, 0.599 mmol) using the general procedure to give **16** (0.221 g, 91%) as yellow microcrystals. Purity (HPLC) 99.0%; mp 122–124 °C. ^1^H NMR (CDCl_3_) δ 8.71 (bdd, *J* = 4.8, 0.6 Hz, 1H), 8.43 (s, 1H), 8.18 (ap dd, *J* = 7.9, 1.7 Hz, 2H), 7.99 (dd, *J* = 2.4, 1.6 Hz, 1H), 7.72–7.79 (m, 2H), 7.57 (bt, *J* = 5.3 Hz, 1H), 7.46–7.54 (m, 3H), 7.40 (d, *J* = 7.8 Hz, 1H), 7.39 (d, *J* = 8.0 Hz, 1H), 7.30 (dd, *J* = 7.4, 5.4 Hz, 1H), 6.83 (ddd, *J* = 8.2, 2.6, 0.8 Hz, 1H), 6.51 (s, 1H), 4.85 (d, *J* = 5.3 Hz, 2H), 3.96 (s, 3H). HRMS calcd. for C_25_H_22_N_5_O (M + H^+^) *m/z* 408.1817, found 408.1824.

5-Phenyl-*N*-(pyridin-2-ylmethyl)-3-(3-(trifluoromethyl)phenyl)pyrazolo[1,5-*a*]pyrimidin-7-amine (**17**)

Synthesised from **10g** (0.224 g, 0.599 mmol) using the general procedure to give **17** (0.220 g, 82%) as pale yellow microcrystals. Purity (HPLC) 99.4%; mp 156–158 °C. ^1^H NMR (CDCl_3_) δ 8.69 (ddd, *J* = 4.9, 1.5, 0.9 Hz, 1H), 8.57 (s, 1H), 8.43 (s, 1H), 8.40 (d, *J* = 7.8 Hz, 1H), 8.18 (ap dd, *J* = 8.3, 1.7 Hz, 1H), 7.73 (td, *J* = 7.7, 1.7 Hz, 1H), 7.61 (t, *J* = 5.1 Hz, 1H), 7.45–7.58 (m, 5H), 7.39 (d, *J* = 7.8 Hz, 1H), 7.29 (dd, *J* = 7.4, 5.0 Hz, 1H), 6.52 (s, 1H), 4.83 (d, *J* = 5.2 Hz, 2H). HRMS calcd. for C_25_H_19_F_3_N_5_ (M + H^+^) *m/z* 446.1587, found 446.1593.

3-(4-Fluorophenyl)-5-phenyl-*N*-(pyridin-2-ylmethyl)pyrazolo[1,5-*a*]pyrimidin-7-amine (**18**)

Synthesised from **10h** (0.200 g, 0.618 mmol) using the general procedure to give **18** (0.224 g, 92%) as yellow microcrystals. Purity (HPLC) 99.9%; mp 191–193 °C. ^1^H NMR (CDCl_3_) δ 8.68 (ddd, *J* = 5.6, 1.6, 0.9 Hz, 1H), 8.36 (s, 1H), 8.18 (ap dd, *J* = 8.9, 5.5 Hz, 2H), 8.13 (ap dd, *J* = 8.3, 5.0 Hz, 2H), 7.73 (td, *J* = 7.7, 1.8 Hz, 1H), 7.56 (t, *J* = 5.1 Hz, 1H), 7.44–7.54 (m, 3H), 7.38 (d, *J* = 7.8 Hz, 1H), 7.28 (dd, *J* = 7.5, 5.7 Hz, 1H), 7.15 (ap t, *J* = 8.8 Hz, 2H), 6.48 (s, 1H), 4.82 (d, *J* = 5.3 Hz, 2H). ^13^C NMR (CDCl_3_) δ 162.55, 160.12, 157.55, 155.09, 149.78, 146.95, 145.49, 141.81, 138.89, 137.25, 130.04, 129.34, 129.31, 128.88, 127.60, 127.57, 127.53, 123.12, 121.61, 115.77, 115.56, 109.03, 83.26, 46.99. HRMS calcd. for C_14_H_19_FN_5_ (M + H^+^) *m/z* 396.1619, found 396.1612.

5-Phenyl-*N*-(pyridin-2-ylmethyl)-3-(*p*-tolyl)pyrazolo[1,5-*a*]pyrimidin-7-amine (**19**)

Synthesised from **10i** (0.192 g, 0.600 mmol) using the general procedure to give **19** (0.191 g, 81%) as yellow microcrystals. Purity (HPLC) 99.6%; mp 172–174 °C. ^1^H NMR (CDCl_3_) δ 8.68 (ddd, *J* = 4.9, 1.7, 0.9 Hz, 1H), 8.38 (s, 1H), 8.14 (p dd, *J* = 8.3, 1.6 Hz, 2H), 8.10 (ap d, *J* = 8.2 Hz, 2H), 7.71 (td, *J* = 7.7, 1.8 Hz, 1H), 7.43–7.53 (m, 4H), 7.38 (d, *J* = 7.8 Hz, 1H), 7.25–7.29 (m, 3H), 6.46 (s, 1H), 4.82 (d, *J* = 5.4 Hz, 2H), 2.39 (s, 3H). HRMS calcd. for C_25_H_22_N_5_ (M + H^+^) *m/z* 392.1875, found 392.1884.

3-(4-Methoxyphenyl)-5-phenyl-*N*-(pyridin-2-ylmethyl)pyrazolo[1,5-a]pyrimidin-7-amine (**20**)

Synthesised from **10j** (0.200 g, 0.596 mmol) using the general procedure to give **20** (0.189 g, 78%) as yellow microcrystals. Purity (HPLC) 99.3%; mp 187–189 °C. ^1^H NMR (CDCl_3_) δ 8.67 (ddd, *J* = 5.8, 1.6, 0.7 Hz, 1H), 8.35 (s, 1H), 8.14 (ap d, *J* = 9.0 Hz, 4H), 7.73 (td, *J* = 7.7, 1.8 Hz, 1H), 7.44–7.53 (m, 4H), 7.39 (d, *J* = 7.9 Hz, 1H), 7.28 (dd, *J* = 7.3, 5.6 Hz, 1H), 7.03 (dt, *J* = 8.9, 2.1 Hz, 1H), 6.46 (s, 1H), 4.83 (d, *J* = 5.4 Hz, 2H), 3.87 (s, 3H). ^13^C NMR (CDCl_3_) δ 157.97, 157.12, 155.27, 149.77, 146.91, 145.26, 141.64, 139.02, 137.22, 129.0, 128.83, 127.56, 127.33, 125.92, 123.06, 121.57, 114.38, 109.80, 82.98, 55.56, 47.05. HRMS calcd. for C_25_H_22_N_5_O (M + H^+^) *m/z* 408.1819, found 408.1811.

5-Phenyl-*N*-(pyridin-2-ylmethyl)-3-(4-(trifluoromethyl)phenyl)pyrazolo[1,5-*a*]pyrimidin-7-amine (**21**)

Synthesised from **10k** (0.224 g, 0.600 mmol) using the general procedure to give **21** (0.224 g, 84%) as yellow microcrystals. Purity (HPLC) 99.1%; mp 188–190 °C. ^1^H NMR (CDCl_3_) δ 8.69 (dd, *J* = 4.8, 0.6 Hz, 1H), 8.44 (s, 1H), 8.34 (d, *J* = 8.1 Hz, 2H), 8.14 (ap dd, *J* = 8.2, 1.7 Hz, 2H), 7.74 (td, *J* = 7.7, 1.8 Hz, 1H), 7.69 (d, *J* = 8.2 Hz, 2H), 7.63 (t, *J* = 5.1 Hz, 1H), 7.47–7.54 (m, 3H), 7.38 (d, *J* = 7.8 Hz, 1H), 7.29 (dd, *J* = 7.1, 5.4 Hz, 1H), 6.52 (s, 1H), 4.83 (d, *J* = 5.2 Hz, 2H). HRMS calcd. for C_25_H_19_F_3_N_5_ (M + H^+^) *m/z* 446.1593, found 446.1594.

Preparation of compounds **22**–**44** of Table 2

5,7-Dichloro-3-(4-fluorophenyl)pyrazolo[1,5-*a*]pyrimidine (**74**)

Sodium (5.1 g, 0.111 mol) was slowly added to absolute ethanol (400 mL) to generate sodium ethoxide solution, and diethyl malonate (16.8 mL, 0.111 mol) was then added, followed by **8h** (19.63 g, 0.111 mol). The mixture was refluxed for 16 h, then cooled, the solvent was removed in *vacuo* and the residue was dissolved in water (400 mL). The solution was stirred rapidly and acidified with concentrated HCl to pH 2. The off-white precipitate was filtered and dried to give crude 3-(4-fluorophenyl)pyrazolo[1,5-a]pyrimidine-5,7-diol (**73**) which was used directly without further purification. ^1^H NMR (DMSO-d_6_) δ 11.59 (bs, 1H), 7.89 (bs, 1H), 7.52 (bs, 1H), 7.19–7.38 (m, 4H), 4.87 (bs, 1H). LRMS [M + H] = 227.1.

A mixture of crude **73** and tetramethylammonium chloride (18.2 g, 0.167 mol) in POCl_3_ (60 mL) was refluxed for 0.5 h. The excess POCl_3_ was removed in *vacuo* and the residue was slurried in DCM (200 mL) and then poured onto ice. The mixture was partitioned between DCM and water and the aqueous was extracted with DCM (2 × 100 mL). The organic fractions were dried and evaporated, and chromatography (DCM, neutral alumina) gave 5,7-dichloro-3-(4-fluorophenyl)pyrazolo[1,5-a]pyrimidine (**74**) (12.748 g, 41%) as a yellow solid. ^1^H NMR (CDCl_3_) δ 8.48 (s, 1H), 7.96 (ap dd, *J* = 8.9, 5.3 Hz, 2H), 7.16 (ap t, *J* = 8.8 Hz, 2H), 7.02 (s, 1H). LRMS [M + H] = 282.1, 284.1.

5-Chloro-3-(4-fluorophenyl)-*N*-(pyridin-2-ylmethyl)pyrazolo[1,5-*a*]pyrimidin-7-amine (**75**)

A solution of **74** (9.31 g, 33.0 mmol) in DCM (240 mL) at 0 °C was treated with diisopropylethylamine (6.90 mL, 39.6 mmol) and then pyridin-2-ylmethanamine (3.74 mL, 36.3 mmol). The solution was stirred at room temperature for 18 h, then partitioned between DCM (200 mL), water and saturated NaHCO_3_ (to basic); the aqueous was extracted with DCM (2 × 100 mL); the organic fractions were combined, dried and evaporated. Chromatography on silica with 3:1 hexanes:EtOAc eluted a coloured impurity, while 1:1 hexanes:EtOAc gave **75** (11.06 g, 95%) as a white solid. ^1^H NMR (CDCl_3_) δ 8.68 (dd, *J* = 4.8, 0.6 Hz, 1H), 8.31 (s, 1H), 7.93 (ap dd, *J* = 8.9, 5.4 Hz, 2H), 7.79 (bs, 1H), 7.75 (td, *J* = 7.7, 1.7 Hz, 1H), 7.34 (d, *J* = 7.9 Hz, 1H), 7.30 (bdd, *J* = 7.4, 5.3 Hz, 1H), 7.12 (ap t, *J* = 8.8 Hz, 2H), 6.05 (s, 1H), 4.71 (d, *J* = 5.1 Hz, 2H). LRMS [M + H] = 354.1, 356.1.

*tert*-Butyl (5-chloro-3-(4-fluorophenyl)pyrazolo[1,5-*a*]pyrimidin-7-yl)(pyridin-2-ylmethyl)carbamate (**76**)

Triethylamine (2.85 mL, 20.4 mmol) and Boc_2_O (4.085 g, 18.7 mmol) were added sequentially to a solution of **75** (6.018 g, 17.0 mmol) and DMAP (0.208 g, 1.70 mmol) in DCM (120 mL) at room temperature. The solution was stirred at room temperature for 16 h, then portioned between DCM, water and sat. aq. NaHCO_3_ (to basic). The aqueous layer was extracted with DCM (2 x 100 mL), and the organic fractions were combined, dried and evaporated. Chromatography on silica (25–33% EtOAc:hexanes) gave **76** (6.388 g, 83%) as a colourless foam. ^1^H NMR (CDCl_3_) δ 8.53 (ddd, *J* = 4.9, 1.7, 0.9 Hz, 1H), 8.39 (s, 1H), 7.97 (ap dd, *J* = 8.9, 5.3 Hz, 2H), 7.67 (td, *J* = 7.7, 1.8 Hz, 1H), 7.43 (d, *J* = 7.9 Hz, 1H), 7.20 (ddd, *J* = 7.5, 4.9, 1.0 Hz, 1H), 7.14 (ap t, *J* = 8.8 Hz, 2H), 6.98 (s, 1H), 5.12 (s, 2H), 1.36 (s, 9H). LRMS [M + H] = 354.1, 356.1.

### 4.4. General Suzuki Procedure

A mixture of **76** (0.100 g, 0.22 mmol), boronic acid (0.88 mmol, 4 eq.) and Na_2_CO_3_ (0.140 g, 1.32 mmol) in toluene (5.5 mL) and water (1.5 mL) was purged with nitrogen in a sealable tube. Pd(PPh_3_)_4_ (0.051 g, 0.044 mmol) was added, and the mixture was purged with nitrogen, sealed and then heated to reflux under nitrogen for 4 h. The mixture was partitioned between EtOAc and water, and the organic fraction was dried (MgSO_4_) and evaporated onto silica gel. Column chromatography on silica gel gave the Boc-protected products.

### 4.5. General Deprotection Procedure

A solution of the Boc-protected product (0.12 mmol) in TFA (10 mL) and DCM (10 mL) was refluxed for 3 h. The solvent was evaporated, and the residue was partitioned between EtOAc, water and saturated aqueous NaHCO_3_ (until basic). The organic fraction was dried (MgSO_4_) and evaporated onto silica gel. Column chromatography on silica gel gave the product, which was recrystallised from DCM/heptane by evaporation of DCM to give compounds **22–44**.

3-(4-Fluorophenyl)-*N*-(pyridin-2-ylmethyl)pyrazolo[1,5-*a*]pyrimidin-7-amine (**22**)

A mixture of **76** (0.500 g, 1.10 mmol), bis(pinacolato)diboron (0.310 g, 1.21 mmol) and KOAc (0.650 g, 6.62 mmol) in DMSO (8 mL, anhydrous) was purged with nitrogen in a sealable tube. PdCl_2_(dppf) (45 mg, 0.055 mmol) was added, and the mixture was heated to 80 °C under nitrogen for 2 h. The mixture was partitioned between EtOAc and water, and the organic fraction was dried and evaporated. Chromatography on silica (33%–50% EtOAc:hexanes) gave **Boc-22** (0.126 g, 27%) as a yellow solid. ^1^H NMR (CDCl_3_) δ 8.52 (ddd, *J =* 4.8, 1.7, 0.9 Hz, 1H), 8.49 (d, *J =* 4.5 Hz, 1H), 8.41 (s, 1H), 8.01 (ap dd, *J =* 8.9, 5.4 Hz, 2H), 7.65 (td, *J =* 7.7, 1.8 Hz, 1H), 7.46 (d, *J =* 7.8 Hz, 1H), 7.09–7.19 (m, 3H), 6.93 (d, *J =* 4.4 Hz, 1H), 5.14 (s, 2H), 1.36 (s, 9H). LRMS [M + H] = 420.2.

Deprotection of **Boc-22** (0.122 g, 0.29 mmol) using the general deprotection procedure gave **22** (0.075 g, 81%) as yellow microcrystals. Purity (HPLC) 99.9%; mp 171–173 °C. ^1^H NMR (CDCl_3_) δ 8.67 (bdd, *J* = 3.5, 0.9 Hz, 1H), 8.33 (s, 1H), 8.32 d, *J* = 5.1 Hz, 1H), 8.01 (ap dd, *J* = 8.8, 5.4 Hz, 2H), 7.73 (td, *J* = 7.7, 1.8 Hz, 1H), 7.60 (bt, *J* = 4.8 Hz, 1H), 7.35 (d, *J* = 7.8 Hz, 1H), 7.28 (dd, *J* = 7.4, 5.7 Hz, 1H), 7.13 (ap t, *J* = 8.8 Hz, 2H), 6.03 (d, *J* = 5.1 Hz, 1H), 4.74 (d, *J* = 5.4 Hz, 2H). HRMS calcd. for C_18_H_15_FN_5_ (M + H^+^) *m/z* 320.1306, found 320.1307.

5-Ethyl-3-(4-fluorophenyl)-*N*-(pyridin-2-ylmethyl)pyrazolo[1,5-*a*]pyrimidin-7-amine (**23**)

A solution of **Boc-24** (0.100 g, 0.225 mmol) in MeOH (30 mL) was purged with nitrogen in a hydrogenation bottle. 10% Pd/C (10 mg) was added, and the mixture was hydrogenated at 50 psi for 18 h. The mixture was filtered and evaporated to give crude **Boc-23**. Deprotection of crude **Boc-23** using the general deprotection procedure gave **23** (0.030 g, 38%) as cream crystals. Purity (HPLC) 97.4%; mp 137–138 °C. ^1^H NMR (CDCl_3_) δ 8.67 (ddd, *J* = 4.9, 1.6, 0.9 Hz, 1H), 8.29 (s, 1H), 8.08 (ap dd, *J* = 8.9, 5.4 Hz, 2H), 7.72 (td, *J* = 7.7, 1.8 Hz, 1H), 7.41 (bt, *J* = 4.9 Hz, 1H), 7.35 (d, *J* = 7.9 Hz, 1H), 7.27 (m, 1H), 7.11 (ap t, *J* = 8.9 Hz, 2H), 6.58 (s, 1H), 4.72 (d, *J* = 5.4 Hz, 2H), 2.81 (q, *J* = 7.6 Hz, 2H), 1.36 (t, *J* = 7.6 Hz, 3H). HRMS calcd. for C_20_H_19_FN_5_ (M + H^+^) *m/z* 348.1619, found 348.1620.

3-(4-Fluorophenyl)-*N*-(pyridin-2-ylmethyl)-5-vinylpyrazolo[1,5-*a*]pyrimidin-7-amine (**24**)

A biphasic solution of **76** (1.00 g, 2.20 mmol), potassium vinyltrifluoroborate (1.175 g, 8.77 mmol) and Na_2_CO_3_ (1.40 g, 13.2 mmol) in toluene (50 mL) and water (15 mL) was purged with nitrogen in a sealable tube. Pd(PPh_3_)_4_ (0.51 g, 0.44 mmol) was added, and the mixture was heated to reflux under nitrogen for 3 h. The mixture was partitioned between EtOAc and water, and the organic fraction was dried and evaporated. Chromatography on silica (2:1 hexanes:EtOAc) gave **Boc-24** (0.922 g, 94%) as an orange foam. ^1^H NMR (CDCl_3_) δ 8.53 (bd, *J* = 4.2 Hz, 1H), 8.38 (s, 1H), 8.07 (ap dd, *J* = 8.8, 5.4 Hz, 2H), 7.66 (td, *J* = 7.7, 1.8 Hz, 2H), 7.49 (d, *J* = 7.9 Hz, 1H), 7.10–7.18 (m, 2H), 7.05 (s, 1H), 6.86 (dd, *J* = 17.6, 10.8 Hz, 1H), 6.24 (d, *J* = 17.6 Hz, 1H), 5.69 (d, *J* = 10.8 Hz, 1H), 5.13 (s, 2H), 1.36 (s, 9H). LRMS [M + H] = 446.2.

Deprotection of **Boc-24** (0.100 g, 0.225 mmol) using the general deprotection procedure gave **24** (0.026 g, 34%) as yellow microcrystals. Purity (HPLC) 97.8%; mp 131–133 °C. ^1^H NMR (CDCl_3_) δ 8.68 (dd, *J* = 3.3, 0.9 Hz, 1H), 8.31 (s, 1H), 8.10 (ap dd, *J* = 8.9, 5.4 Hz, 2H), 7.73 (td, *J* = 7.7, 1.8 Hz, 1H), 7.50 (bt, *J* = 5.0 Hz, 1H), 7.36 (d, *J* = 7.9 Hz, 1H), 7.28 (dd, *J* = 7.4, 5.8 Hz, 1H), 7.12 (ap t, *J* = 8.8 Hz, 2H), 6.80 (dd, *J* = 17.5, 10.7 Hz, 1H), 6.30 (dd, *J* = 17.5, 1.0 Hz, 1H), 6.12 (s, 1H), 5.61 (dd, *J* = 10.7, 1.0 Hz, 1H), 4.76 (d, *J* = 5.3 Hz, 2H). HRMS calcd. for C_20_H_17_FN_5_ (M + H^+^) *m/z* 346.1463, found 346.1459.

5-Cyclopropyl-3-(4-fluorophenyl)-*N*-(pyridin-2-ylmethyl)pyrazolo[1,5-*a*]pyrimidin-7-amine (**25**)

Reaction of **76** (0.100 g, 0.22 mmol) and cyclopropylboronic acid (0.076 g, 0.88 mmol) using the general Suzuki procedure gave **Boc-25** (0.090 g, 89%) as a yellow solid. ^1^H NMR (CDCl_3_) δ 8.53 (ddd, *J* = 4.8, 1.7, 0.9 Hz, 1H), 8.33 (s, 1H), 8.02 (ap dd, *J* = 8.9, 5.4 Hz, 2H), 7.66 (td, *J* = 7.7, 1.8 Hz, 1H), 7.49 (d, *J* = 7.8 Hz, 1H), 7.18 (ddd, *J* = 7.5, 4.9, 1.0 Hz, 1H), 7.11 (ap t, *J* = 8.8 Hz, 2H), 6.78 (s, 1H), 5.11 (s, 2H), 2.03–2.10 (m, 1H), 1.36 (s, 9H), 1.19–1.22 (m, 2H), 1.08–1.13 (m, 2H). LRMS [M + H] = 460.3.

Deprotection of **Boc-25** (0.085 g, 0.19 mmol) using the general deprotection procedure gave **25** (0.054 g, 81%) as cream needles. Purity (HPLC) 100%; mp 149–151 °C. ^1^H NMR (CDCl_3_) δ 8.66 (ddd, *J* = 4.9, 1.6, 0.9 Hz, 1H), 8.27 (s, 1H), 8.05 (ap dd, *J* = 8.9, 5.4 Hz, 2H), 7.72 (dt, *J* = 7.7, 1.8 Hz, 1H), 7.35 (d, *J* = 7.9 Hz, 2H), 7.28 (dd, *J* = 7.4, 5.7 Hz, 1H), 7.10 (ap t, *J* = 8.9 Hz, 2H), 5.99 (s, 1H), 4.73 (d, *J* = 5.4 Hz, 2H), 1.96–2.04 (m, 1H), 1.18–1.23 (m, 2H), 1.00–1.05 (m, 2H). ^13^C NMR (CDCl_3_) δ 164.80, 162.29, 159.87, 155.34, 149.74, 146.08, 145.56, 141.35, 137.19, 129.56, 129.53, 127.20, 127.13, 123.02, 121.51, 115.61, 115.40, 107.22, 85.00, 46.96, 18.02, 10.74. HRMS calcd. for C_21_H_19_FN_5_ (M + H^+^) *m/z* 360.1624, found 360.1628.

5-(Cyclopent-1-en-1-yl)-3-(4-fluorophenyl)-*N*-(pyridin-2-ylmethyl)pyrazolo[1,5-*a*]pyrimidin-7-amine (**26**)

Reaction of **76** (0.100g, 0.22 mmol) and cyclopent-1-en-1-ylboronic acid (0.099 g, 0.88 mmol) using the general Suzuki procedure gave **Boc-26** (0.084 g, 79%) as an orange solid. ^1^H NMR (CDCl_3_) δ 8.53 (ddd, *J* = 4.8, 1.6, 0.8 Hz, 1H), 8.36 (s, 1H), 8.11 (ap dd, *J* = 9.0, 5.4 Hz, 2H), 7.65 (td, *J* = 7.7, 1.8 Hz, 1H), 7.50 (d, *J* = 7.9 Hz, 1H), 7.18 (ddd, *J* = 7.4, 4.9, 1.0 Hz, 1H), 7.13 (ap t, *J* = 8.9 Hz, 2H), 7.03 (s, 1H), 6.61–6.64 (m, 1H), 5.12 (s, 2H), 2.89–2.94 (m, 2H), 2.61–2.66 (m, 2H), 2.02–2.11 (m, 2H), 1.36 (s, 9H). LRMS [M + H] = 486.3.

Deprotection of **Boc-26** (0.025 g, 0.052 mmol) using the general deprotection procedure gave **26** (0.014 g, 71%) as yellow microcrystals. Purity (HPLC) 98.8%; mp 177–179 °C. ^1^H NMR (CDCl_3_) δ 8.66 (ddd, *J* = 4.9, 1.6, 0.9 Hz, 1H), 8.31 (s, 1H), 8.13 (ap dd, *J* = 8.9, 5.4 Hz, 2H), 7.72 (td, *J* = 7.7, 1.8 Hz, 1H), 7.39 (t, *J* = 5.2 Hz, 1H), 7.36 (d, *J* = 7.9 Hz, 1H), 7.24–7.29 (m, 1H), 7.11 (ap t, *J* = 8.9 Hz, 2H), 6.64–6.68 (m, 1H), 6.17 (s, 1H), 4.75 (d, *J* = 5.3 Hz, 2H), 2.87–2.93 (m, 2H), 2.58–2.64 (m, 2H), 2.03–2.10 (m, 2H). ^13^C NMR (CDCl_3_) δ 162.35, 159.93, 155.39, 155.24, 149.67, 146.24, 145.23, 144.07, 141.31, 137.12, 133.82, 129.4, 129.37, 127.30, 127.22, 122.96, 127.22, 122.96, 121.50, 155.59, 115.38, 108.47, 83.14, 46.91, 33.90, 32.67, 23.45. HRMS calcd. for C_23_H_21_FN_5_ (M + H^+^) *m/z* 386.1776, found 386.1777.

5-(Cyclohex-1-en-1-yl)-3-(4-fluorophenyl)-*N*-(pyridin-2-ylmethyl)pyrazolo[1,5-*a*]pyrimidin-7-amine (**27**)

Reaction of **76** (0.100g, 0.22 mmol) and cyclohex-1-en-1-ylboronic acid (0.111 g, 0.88 mmol) using the general Suzuki procedure gave **Boc-27** (0.076 g, 69%) as an orange solid. ^1^H NMR (CDCl_3_) δ 8.53 (ddd, *J* = 4.8, 1.6, 0.8 Hz, 1H), 8.35 (s, 1H), 8.10 (ap dd, *J* = 8.9, 5.4 Hz, 2H), 7.65 (td, *J* = 7.7, 1.8 Hz, 1H), 7.50 (d, *J* = 7.9 Hz, 1H), 7.17 (ddd, *J* = 7.6, 4.9, 1.0 Hz, 1H), 7.14 (ap t, *J* = 8.8 Hz, 2H), 7.10 (s, 1H), 6.72–6.77 (m, 1H), 5.12 (s, 2H), 2.62–2.67 (m, 2H), 2.27–2.33 (m, 2H), 1.78–1.83 (m, 2H), 1.66–1.73 (m, 2H), 1.36 (s, 9H). LRMS [M + H] = 500.3.

Deprotection of **Boc-27** (0.030 g, 0.060 mmol) using the general deprotection procedure gave **27** (0.022 g, 92%) as yellow microcrystals. Purity (HPLC) 97.2%; mp 199–201 °C. ^1^H NMR (CDCl_3_) δ 8.67 (ddd, *J* = 4.9, 1.6, 0.9 Hz, 1H), 8.30 (s, 1H), 8.12 (ap dd, *J* = 9.0, 5.5 Hz, 2H), 7.72 (td, *J* = 7.7, 1.8 Hz, 1H), 7.33–7.39 (m, 2H), 7.27 (dd, *J* = 7.6, 5.5 Hz, 1H), 7.12 (ap t, *J* = 8.9 Hz, 2H), 6.74–6.80 (m, 1H), 6.16 (s, 1H), 4.75 (d, *J* = 5.3 Hz, 2H), 2.61–2.64 (m, 2H), 2.25–2.33 (m, 2H), 1.79–1.85 (m, 2H), 1.67–1.73 (m, 2H). ^13^C NMR (CDCl_3_) δ 162.42, 160.00, 159.55, 155.39, 149.77, 146.47, 145.16, 141.44, 137.21, 137.13, 131.04, 129.53, 129.50, 127.40, 127.32, 123.04, 121.56, 115.69, 115.48, 108.46, 82.17, 47.01, 26.35, 25.85, 22.98, 22.29. HRMS calcd. for C_24_H_23_FN_5_ (M + H^+^) *m/z* 400.1932, found 400.1933.

5-(2-Fluorophenyl)-3-(4-fluorophenyl)-*N*-(pyridin-2-ylmethyl)pyrazolo[1,5-*a*]pyrimidin-7-amine (**28**)

Reaction of **76** (0.100 g, 0.22 mmol) and (2-fluorophenyl)boronic acid (0.123 g, 0.88 mmol) using the general Suzuki conditions gave **Boc-28** (0.060 g, 53%) as a yellow solid. ^1^H NMR (CDCl_3_) δ 8.51 (bd, *J* = 4.2 Hz, 1H), 8.45 (s, 1H), 8.20 (td, *J* = 7.8, 1.8 Hz, 1H), 8.12 (ap dd, *J* = 8.8, 5.4 Hz, 2H), 7.66 (td, *J* = 7.7, 1.8 Hz, 1H), 7.43–7.52 (m, 3H), 7.33 (td, *J* = 7.8, 1.0 Hz, 1H), 7.13–7.21 (m, 4H), 5.20 (s, 2H), 1.39 (s, 9H). LRMS [M + H] = 514.2.

Deprotection of **Boc-28** (0.048 g, 0.94 mmol) using the general deprotection procedure gave **28** (0.045 g, 95%) as orange microcrystals. Purity (HPLC) 97.4%; mp 181–184 °C. ^1^H NMR (CDCl_3_) δ 8.69 (dd, *J* = 4.8, 0.6 Hz, 1H), 8.37 (s, 1H), 8.22 (td, *J* = 7.8, 1.8 Hz, 1H), 8.14 (ap dd, *J* = 8.9, 5.4 Hz, 2H), 7.73 (td, *J* = 7.7 Hz, 1.8 Hz, 1H), 7.61 (t, *J* = 5.0 Hz, 1H), 7.40–7.46 (m, 1H), 7.38 (d, *J* = 7.8 Hz, 1H), 7.32 (td, *J* = 7.7, 1.2 Hz, 1H), 7.28 (dd, *J* = 7.5, 5.7 Hz, 1H), 7.11–7.20 (m, 3H), 6.62 (s, 1H), 4.80 (d, *J* = 5.2 Hz, 2H). ^13^C NMR (CDCl_3_) δ 162.57, 162.32, 160.14, 159.83, 154.92, 153.71, 153.69, 149.77, 146.52, 145.33, 141.76, 137.18, 131.56, 131.53, 131.39, 131.31, 129.26, 129.23, 127.76, 127.63, 127.55, 127.19, 127.08, 124.79, 124.75, 123.08, 121.70, 116.63, 116.39, 115.79, 115.58, 109.13, 87.10, 86.99, 46.85. HRMS calcd. for C_28_H_27_FN_5_ (M + H^+^) *m/z* 452.2245, found 452.2244.

3-(4-Fluorophenyl)-5-(2-methoxyphenyl)-*N*-(pyridin-2-ylmethyl)pyrazolo[1,5-*a*]pyrimidin-7-amine (**29**)

Reaction of **76** (0.100g, 0.22 mmol) and 2-methoxyphenylboronic acid (0.134 g, 0.88 mmol) using the general Suzuki conditions gave **Boc-29** (0.090 g, 78%) as a yellow solid. ^1^H NMR (CDCl_3_) δ 8.51 (ddd, *J* = 4.8, 1.6, 0.8 Hz, 1H), 8.40 (s, 1H), 8.12 (ap dd, *J* = 8.9, 5.3, Hz, 2H), 8.06 (dd, *J* = 7.7, 1.8 Hz, 1H), 7.65 (td, *J* = 7.7, 1.8 Hz, 1H), 7.56 (s, 1H), 7.53 (d, *J* = 7.8 Hz, 1H), 7.43 (ddd, *J* = 7.4, 4.9, 1.0 Hz, 1H), 7.09–7.18 (m, 4H), 6.99 (d, *J* = 8.3 Hz, 1H), 5.17 (s, 2H), 3.81 (s, 3H), 1.37 (s, 9H). LRMS [M + H] = 526.3.

Deprotection of **Boc-29** (0.086 g, 0.16 mmol) using the general deprotection procedure gave **29** (0.055 g, 79%) as cream microcrystals. Purity (HPLC) 99.4%; mp 150–153 °C. ^1^H NMR (CDCl_3_) δ 8.67 (ddd, *J* = 4.9, 1.6, 0.7 Hz, 1H), 8.35 (s, 1H), 8.15 (ap dd, *J* = 8.9, 5.3 Hz, 2H), 8.02 (dd, *J* = 7.6, 1.8 Hz, 1H), 7.72 (td, *J* = 7.7, 1.8 Hz, 1H), 7.37–7.44 (m, 3H), 7.28 (ddd, *J* = 8.2, 2.6, 0.8 Hz, 1H), 7.08–7.15 (m, 3H), 7.00 (dd, *J* = 8.3, 0.7 Hz, 1H), 6.69 (s, 1H), 4.79 (d, *J* = 5.5 Hz, 2H), 3.82 (s, 3H). HRMS calcd. for C_25_H_21_FN_5_O (M + H^+^) *m/z* 426.1725, found 426.1723.

3-(4-Fluorophenyl)-*N*-(pyridin-2-ylmethyl)-5-(*m*-tolyl)pyrazolo[1,5-*a*]pyrimidin-7-amine (**30**)

Reaction of **76** (0.100 g, 0.22 mmol) and *m*-tolylboronic acid (0.120 g, 0.88 mmol) using the general Suzuki conditions gave **Boc-30** (0.069 g, 62%) as a yellow solid. ^1^H NMR (CDCl_3_) δ 8.53 (ddd, *J* = 4.9, 1.6, 0.9 Hz, 1H), 8.42 (s, 1H), 8.15 (ap dd, *J* = 8.9, 5.4 Hz, 2H), 7.89–7.92 (m, 2H), 7.66 (td, *J* = 7.7, 1.8 Hz, 1H), 7.51 (d, *J* = 7.8 Hz, 1H), 7.41 (t, *J* = 7.7 Hz, 1H), 7.39 (s, 1H), 7.31 (d, *J* = 7.7 Hz, 1H), 7.14–7.20 (m, 3H), 5.19 (s, 2H), 2.47 (s, 3H), 1.38 (s, 9H). LRMS [M + H] = 510.2.

Deprotection of **Boc-30** (0.060 g, 0.12 mmol) using the general deprotection procedure gave **30** (0.042 g, 87%) as a cream solid. Purity (HPLC) 99.6%; mp 151–153 °C. ^1^H NMR (CDCl_3_) δ 8.69 (ddd, *J* = 4.9, 1.6, 0.9 Hz, 1H), 8.35 (s, 1H), 8.17 (ap dd, *J* = 9.0, 5.4 Hz, 2H), 7.91–7.93 (m, 2H), 7.73 (td, *J* = 7.7, 1.8 Hz, 1H), 7.55 (t, *J* = 5.1 Hz, 1H), 7.38–7.42 (m, 2H), 7.27–7.30 (m, 2H), 7.15 (ap t, *J* = 8.8 Hz, 2H), 6.47 (s, 1H), 4.83 (d, *J* = 5.3 Hz, 2H), 2.47 (s, 3H). ^13^C NMR (CDCl_3_) δ 162.54, 160.11, 157.83, 155.12, 149.77, 146.89, 145.50, 141.80, 138.92, 138.49, 137.24, 130.83, 129.37, 129.34, 128.80, 128.19, 127.62, 127.55, 124.79, 123.10, 121.61, 115.77, 115.56, 108.99, 83.41, 46.98, 21.84. HRMS calcd. for C_25_H_21_FN_5_ (M + H^+^) *m/z* 410.1776, found 410.1775.

3-(4-Fluorophenyl)-5-(3-methoxyphenyl)-*N*-(pyridin-2-ylmethyl)pyrazolo[1,5-*a*]pyrimidin-7-amine (**31**)

Reaction of **76** (0.100 g, 0.22 mmol) and (3-methoxyphenyl)boronic acid (0.134 g, 0.88 mmol) using the general Suzuki conditions gave **Boc-31** (0.109 g, 94%) as a yellow solid. ^1^H NMR (CDCl_3_) δ 8.54 (ddd, *J* = 4.9, 1.7, 0.9 Hz, 1H), 8.42 (s, 1H), 8.15 (ap dd, *J* = 8.9, 5.4 Hz, 2H), 7.70 (t, *J* = 1.7 Hz, 1H), 7.66 (td, *J* = 7.6, 1.8 Hz, 2H), 7.50 (d, *J* = 7.8 Hz, 1H), 7.43 (t, *J* = 8.0 Hz, 1H), 7.40 (s, 1H), 7.14–7.20 (m, 3H), 7.04 (ddd, *J* = 8.2, 2.6, 0.8 Hz, 1H), 5.18 (s, 2H), 3.92 (s, 3H), 1.38 (s, 9H). LRMS [M + H] = 526.3.

Deprotection of **Boc-31** (0.105 g, 0.20 mmol) using the general deprotection procedure gave **31** (0.068 g, 80%) as pale yellow microcrystals. Purity (HPLC) 99.6%; mp 147–149 °C. ^1^H NMR (CDCl_3_) δ 8.69 (ddd, *J* = 4.9, 1.6, 0.7 Hz, 1H), 8.36 (s, 1H), 8.18 (ap dd, *J* = 8.9 5.3 Hz, 2H), 7.71–7.76 (m, 2H), 7.68 (ddd, *J* = 7.7, 1.6, 1.0 Hz, 1H), 7.56 (t, *J* = 5.2 Hz, 1H), 7.42 (t, *J* = 8.0 Hz, 1H), 7.39 (d, *J* = 7.8 Hz, 1H), 7.28 (ddd, *J* = 7.5, 5.0, 1.0 Hz, 1H), 7.15 (ap t, *J* = 8.8 Hz, 2H), 7.02 (ddd, *J* = 8.2, 2.6, 0.8 Hz, 1H), 6.46 (s, 1H), 4.82 (d, *J* = 5.3 Hz, 2H), 3.92 (s, 3H). HRMS calcd. for C_25_H_21_FN_5_O (M + H^+^) *m/z* 426.1725, found 426.1725.

3,5-Bis(4-fluorophenyl)-*N*-(pyridin-2-ylmethyl)pyrazolo[1,5-*a*]pyrimidin-7-amine (**32**)

Reaction of **76** (0.100g, 0.22 mmol) and (4-fluorophenyl)boronic acid (0.123 g, 0.88 mmol) using the general Suzuki procedure gave **Boc-32** (0.105 g, 93%) as a yellow solid. ^1^H NMR (CDCl_3_) δ 8.54 (ddd, *J* = 4.8, 1.6, 0.8 Hz, 1H), 8.42 (s, 1H), 8.09–8.15 (m, 4H), 7.67 (td, *J* = 7.7, 1.8 Hz, 1H), 7.50 (d, *J* = 7.9 Hz, 1H), 7.40 (s, 1H), 7.15–7.23 (m, 5H), 5.17 (s, 2H), 1.38 (s, 9H). LRMS [M + H] = 514.1.

Deprotection of **Boc-32** (0.097 g, 0.19 mmol) using the general deprotection procedure gave **32** (0.067 g, 86%) as pale yellow microcrystals. Purity (HPLC) 99.6%; mp 181–183 °C. ^1^H NMR (CDCl_3_) δ 8.69 (ddd, *J* = 4.9, 1.6, 0.9 Hz, 1H), 8.35 (s, 1H), 8.09–8.18 (m, 4H), 7.74 (td, *J* = 7.7, 1.8 Hz, 1H), 7.58 (t, *J* = 5.1 Hz, 1H), 7.38 (d, *J* = 7.8 Hz, 1H), 7.29 (dd, *J* = 7.5, 5.8 Hz, 1H), 7.12–7.22 (m, 4H), 6.42 (s, 1H), 4.82 (d, *J* = 5.3 Hz, 2H). HRMS calcd. for C_24_H_18_F_2_N_5_ (M + H^+^) *m/z* 414.1530, found 414.1535.

3-(4-Fluorophenyl)-*N*-(pyridin-2-ylmethyl)-5-(*p*-tolyl)pyrazolo[1,5-*a*]pyrimidin-7-amine (**33**)

Reaction of **76** (0.100 g, 0.22 mmol) and *p*-tolylboronic acid (0.120 g, 0.88 mmol) using the general Suzuki procedure gave **Boc-33** (0.094 g, 84%) as an orange solid. ^1^H NMR (CDCl_3_) δ 8.53 (ddd, *J* = 4.8, 1.6, 0.8 Hz, 1H), 8.41 (s, 1H), 8.14 (ap dd, *J* = 8.9, 5.4 Hz, 2H), 8.01 (d, *J* = 8.2 Hz, 2H), 7.66 (td, *J* = 7.7, 1.8 Hz, 1H), 7.50 (d, *J* = 7.9 Hz, 1H), 7.38 (s, 1H), 7.32 (d, *J* = 8.0 Hz, 2H), 7.14–7.20 (m, 3H), 5.18 (s, 2H), 2.45 (s, 3H), 1.38 (s, 9H). LRMS [M + H] = 510.2. 

Deprotection of **Boc-33** (0.080 g, 0.16 mmol) using the general deprotection procedure gave **33** (0.052 g, 81%) as orange microcrystals. Purity (HPLC) 99.5%; mp 180–182 °C. ^1^H NMR (CDCl_3_) δ 8.69 (ddd, *J* = 4.9, 1.6, 0.8 Hz, 1H), 8.34 (s, 1H), 8.17 (ap dd, *J* = 8.9, 5.2 Hz, 2H), 8.02 (d, *J* = 8.2 Hz, 2H), 7.72 (td, *J* = 7.7, 1.8 Hz, 1H), 7.52 (t, *J* = 5.2 Hz, 1H), 7.38 (d, *J* = 7.8 Hz, 1H), 7.31 (d, *J* = 7.9 Hz, 2H), 7.28 (obscured, 1H), 7.15 (ap d, *J* = 8.8 Hz, 2H), 6.46 (s, 1H), 4.82 (d, *J* = 5.3 Hz, 2H), 2.43 (s, 3H). HRMS calcd. for C_25_H_21_FN_5_ (M + H^+^) *m/z* 410.1776, found 410.1775.

3-(4-Fluorophenyl)-5-(4-methoxyphenyl)-*N*-(pyridin-2-ylmethyl)pyrazolo[1,5-*a*]pyrimidin-7-amine (**34**)

Reaction of **76** (0.100g, 0.22 mmol) and (4-methoxyphenyl)boronic acid (0.134 g, 0.88 mmol) using the general Suzuki procedure gave **Boc-34** (0.092 g, 80%) as a yellow solid. ^1^H NMR (CDCl_3_) δ 8.54 (ddd, *J* = 4.8, 1.7, 0.9 Hz, 1H), 8.39 (s, 1H), 8.14 (ap dd, *J* = 8.9, 5.4 Hz, 2H), 8.08 (ap d, *J* = 8.9 Hz, 2H), 7.66 (td, *J* = 7.7, 1.8 Hz, 1H), 7.51 (d, *J* = 8.5 Hz, 1H), 7.37 (s, 1H), 7.14–7.19 (m, 3H), 7.03 (ap d, *J* = 8.9 Hz, 2H), 5.17 (s, 2H), 3.90 (s, 3H), 1.37 (s, 9H). LRMS [M + H] = 526.3.

Deprotection of **Boc-34** (0.088 g, 0.17 mmol) using the general deprotection procedure gave **34** (0.058 g, 81%) as cream microcrystals. Purity (HPLC) 99.8%; mp 179–181 °C. ^1^H NMR (CDCl_3_) δ 8.69 (ddd, *J* = 4.9, 1.6, 0.9 Hz, 1H), 8.33 (s, 1H), 8.17 (ap dd, *J* = 9.0, 5.4 Hz, 2H), 8.10 (ap d, *J* = 8.9 Hz, 2H), 7.73 (td, *J* = 7.7, 1.8 Hz, 1H), 7.50 (t, *J* = 5.1 Hz, 1H), 7.38 (d, *J* = 7.8 Hz, 1H), 7.28 (dd, *J* = 7.4, 5.7 Hz, 1H), 7.15 (ap t, *J* = 8.8 Hz, 2H), 7.03 (ap d, *J* = 8.9 Hz, 2H), 6.42 (s, 1H), 4.82 (d, *J* = 5.3 Hz, 2H), 3.89 (s, 3H). ^13^C NMR (CDCl_3_) δ 162.47, 161.39, 160.05, 157.15, 155.21, 149.76, 146.84, 145.51, 141.70, 137.23, 131.36, 129.47, 129.44, 128.96, 127.51, 127.44, 123.08, 121.59, 115.73, 115.51, 114.21, 108.65, 82.64, 55.62, 47.01. HRMS calcd. for C_25_H_21_FN_5_O (M + H^+^) *m/z* 426.1730, found 426.1733.

3-(4-Fluorophenyl)-5-(4-isopropylphenyl)-*N*-(pyridin-2-ylmethyl)pyrazolo[1,5-*a*]pyrimidin-7-amine (**35**)

Reaction of **76** (0.100 g, 0.22 mmol) and (4-isopropylphenyl)boronic acid (0.145 g, 0.88 mmol) using the general Suzuki procedure gave **Boc-35** (0.101 g, 86%) as a yellow solid. ^1^H NMR (CDCl_3_) δ 8.54 (ddd, *J* = 4.8, 1.7, 0.9 Hz, 1H), 8.41 (s, 1H), 8.15 (ap dd, *J* = 8.9, 5.4 Hz, 2H), 8.03 (d, *J* = 8.4 Hz, 2H), 7.66 (td, *J* = 7.7, 1.8 Hz, 1H), 7.50 (d, *J* = 7.9 Hz, 1H), 7.36–7.40 (m, 3H), 7.13–7.20 (m, 3H), 5.18 (s, 2H), 2.99 (sept, *J* = 6.9 Hz, 1H), 1.38 (s, 9H), 1.30 (d, *J* = 6.9 Hz, 6H). LRMS [M + H] = 538.2.

Deprotection of **Boc-35** (0.096 g, 0.18 mmol) using the general deprotection procedure gave **35** (0.078 g, 99%) as pale yellow microcrystals. Purity (HPLC) 99.5%; mp 88–90 °C. ^1^H NMR (CDCl_3_) δ 8.68 (dd, *J* = 4.9, 0.6 Hz, 1H), 8.35 (s, 1H), 8.18 (ap dd, *J* = 8.9, 5.5 Hz, 2H), 8.05 (d, *J* = 8.3 Hz, 2H), 7.72 (td, *J* = 7.7, 1.8 Hz, 1H), 7.51 (t, *J* = 5.3 Hz, 1H), 7.35–7.40 (m, 3H), 7.28 (dd, *J* = 6.8, 5.0 Hz, 1H), 7.14 (ap t, *J* = 8.8 Hz, 2H), 6.45 (s, 1H), 4.83 (d, *J* = 5.4 Hz, 2H), 2.99 (sept, *J* = 7.0 Hz, 1H), 1.31 (d, *J* = 7.0 Hz, 6H). ^13^C NMR (CDCl_3_) δ 162.50, 160.07, 157.66, 155.22, 151.17, 149.78, 146.89, 145.54, 141.71, 137.23, 136.54, 129.42, 129.39, 127.61, 127.56, 127.48, 127.00, 123.09, 121.58, 115.73, 115.51, 108.83, 83.16, 47.04, 34.24, 32.09, 29.23, 24.12, 22.90, 14.33. HRMS calcd. for C_27_H_25_FN_5_ (M + H^+^) *m/z* 438.2094, found 438.2103.

5-(4-(*Tert*-butyl)phenyl)-3-(4-fluorophenyl)*-N-*(pyridin-2-ylmethyl)pyrazolo[1,5-*a*]pyrimidin-7-amine (**36**)

Reaction of **76** (0.100 g, 0.22 mmol) and (4-*tert*-butylphenyl)boronic acid (0.157 g, 0.88 mmol) using the general Suzuki procedure gave **Boc-36** (0.100 g, 82%) as an orange solid. ^1^H NMR (CDCl_3_) δ 8.54 (ddd, *J* = 4.8, 1.7, 0.9 Hz, 1H), 8.41 (s, 1H), 8.16 (ap dd, *J* = 8.9, 5.4 Hz, 2H), 8.04 (ap d, *J* = 8.6 Hz, 2H), 7.66 (td, *J* = 7.7, 1.8 Hz, 1H), 7.55 (ap d, *J* = 8.6 Hz, 2H), 7.49 (d, *J* = 7.9 Hz, 1H), 7.38 (s, 1H), 7.13–7.19 (m, 3H), 5.18 (s, 2H), 1.38 (s, 18H). LRMS [M + H] = 552.3.

Deprotection of **Boc-36** (0.070 g, 0.17 mmol) using the general deprotection procedure gave **36** (0.070 g, 90%) as orange microcrystals. Purity (HPLC) 99.5%; mp 150–152 °C. ^1^H NMR (CDCl_3_) δ 8.69 (ddd, *J* = 4.9, 1.6, 0.9 Hz, 1H), 8.35 (s, 1H), 8.19 (ap dd, *J* = 8.9, 5.5 Hz, 2H), 8.06 (ap d, *J* = 8.6 Hz, 2H), 7.72 (td, *J* = 7.7, 1.8 Hz, 1H), 7.53 (ap d, *J* = 8.6 Hz, 2H), 7.51 (t, *J* = 5.3 Hz, 1H), 7.38 (d, *J* = 7.8 Hz, 1H), 7.28 (ddd, *J* = 7.5, 4.7, 0.8 Hz, 1H), 7.14 (ap t (*J* = 8.8 Hz, 2H), 6.46 (s, 1H), 4.82 (d, *J* = 5.3 Hz, 2H), 1.38 (s, 9H). ^13^C NMR (CDCl_3_) δ 162.50, 160.07, 157.59, 155.24, 153.40, 149.80, 146.90, 145.56, 141.71, 137.24, 136.13, 129.42, 129.39, 127.55, 127.48, 127.33, 125.86, 123.09, 121.57, 115.73, 115.52, 108.84, 83.18, 47.06, 35.01, 31.49. HRMS calcd. for C_28_H_27_FN_5_ (M + H^+^) *m/z* 452.2245, found 452.2244.

3-(4-Fluorophenyl)-*N*-(pyridin-2-ylmethyl)-5-(4-(trifluoromethyl)phenyl)pyrazolo[1,5-*a*]pyrimidin-7-amine (**37**)

Reaction of **76** (0.100g, 0.22 mmol) and (4-(trifluoromethyl)phenyl)boronic acid (0.063 g, 0.33 mmol) using the general Suzuki procedure gave **Boc-37** (0.092 g, 74%) as a yellow solid. ^1^H NMR (CDCl_3_) δ 8.55 (ddd, *J* = 4.9, 1.6, 0.8 Hz, 1H), 8.45 (s, 1H), 8.23 (d, *J* = 8.1 Hz, 1H), 8.13 (ap dd, *J* = 8.9, 5.4 Hz, 2H), 7.78 (d, *J* = 8.2 Hz, 2H), 7.68 (td, *J* = 7.7, 1.8 Hz, 1H), 7.50 (d, *J* = 7.8 Hz, 1H), 7.49 (s, 1H), 7.15–7.22 (m, 3H), 5.19 (s, 2H), 1.38 (s, 9H). LRMS [M + H] = 564.2.

Deprotection of **Boc-37** (0.082 g, 0.15 mmol) using the general deprotection procedure gave **37** (0.066 g, 98%) as a yellow solid. Purity (HPLC) 98.6%; mp 170–171 °C. ^1^H NMR (CDCl_3_) δ 8.70 (ddd, *J* = 4.9, 1.6, 0.9 Hz, 1H), 8.38 (s, 1H), 8.23 (d, *J* = 8.1 Hz, 2H), 8.15 (ap dd, *J* = 8.9, 5.2 Hz, 2H), 7.71–7.79 (m, 3H), 7.67 (t, *J* = 5.2 Hz, 1H), 7.39 (d, *J* = 7.8 Hz, 1H), 7.30 (dd, *J* = 7.5, 4.8 Hz, 1H), 7.16 (ap t, *J* = 8.7 Hz, 2H), 6.49 (s, 1H), 4.83 (d, *J* = 5.2 Hz, 2H). HRMS calcd. for C_25_H_18_F_4_N_5_ (M + H^+^) *m/z* 464.1498, found 464.1498.

3-(4-Fluorophenyl)-*N*-(pyridin-2-ylmethyl)-5-(4-(trifluoromethoxy)phenyl)pyrazolo[1,5-*a*]pyrimidin-7-amine (**38**)

Reaction of **76** (0.100g, 0.22 mmol) and (4-(trifluoromethoxy)phenyl)boronic acid (0.181 g, 0.88 mmol) using the general Suzuki procedure gave **Boc-38** (0.107 g, 84%) as a yellow solid. ^1^H NMR (CDCl_3_) δ 8.55 (ddd, *J* = 4.8, 1.7, 0.8 Hz, 1H), 8.43 (s, 1H), 8.09–8.18 (m, 4H), 7.67 (*J* = 7.7, 1.8 Hz, 1H), 7.49 (d, *J* = 7.8 Hz, 1H), 7.43 (s, 1H), 7.36 (d, *J* = 8.0 Hz, 2H), 7.14–7.22 (m, 3H), 5.18 (s, 2H), 1.38 (s, 9H). LRMS [M + H] = 580.2.

Deprotection of **Boc-38** (0.093 g, 0.16 mmol) using the general deprotection procedure gave **38** (0.063 g, 82%) as cream microcrystals. Purity (HPLC) 96.2%; mp 181–183 °C. ^1^H NMR (CDCl_3_) δ 8.69 (ddd, *J* = 4.9, 1.6, 0.9 Hz, 1H), 8.36 (s, 1H), 8.12–8.18 (m, 4H), 7.74 (td, *J* = 7.7, 1.8 Hz, 1H), 7.62 (t, *J* = 5.2 Hz, 1H), 7.38 (d, *J* = 7.8 Hz, 1H), 7.34 (dd, *J* = 8.9, 0.8 Hz, 2H), 7.29 (dd, *J* = 7.4, 5.8 Hz, 1H), 7.15 (ap t, *J* = 8.8 Hz, 2H), 6.44 (s, 1H), 4.82 (d, *J* = 5.3 Hz, 2H). ^13^C NMR (CDCl_3_) δ 162.62, 160.19, 156.06, 154.91, 150.64, 150.62, 149.81, 147.03, 145.34, 141.98, 137.49, 137.29, 129.17, 129.11, 127.63, 127.56, 124.57, 123.19, 121.97, 121.65, 121.11, 119.41, 116.86, 115.82, 115.61, 109.25, 83.03, 46.98. HRMS calcd. for C_25_H_18_F_4_N_5_O (M + H^+^) *m/z* 480.1442, found 480.1443.

3-(4-Fluorophenyl)-*N*-(pyridin-2-ylmethyl)-5-(pyridin-3-yl)pyrazolo[1,5-*a*]pyrimidin-7-amine (**39**)

Reaction of **76** (0.100 g, 0.22 mmol) and pyridin-3-ylboronic acid (0.108 g, 0.88 mmol) using the general Suzuki procedure gave **Boc-39** (0.060 g, 55%) as a yellow solid. ^1^H NMR (CDCl_3_) δ 9.32 (dd, *J* = 2.2, 0.6 Hz, 1H), 8.73 (dd, *J* = 4.8, 1.7 Hz, 1H), 8.55 (ddd, *J* = 4.8, 1.6, 0.8 Hz, 1H), 8.45 (s, 1H), 8.44 (ddd, *J* = 8.0, 2.2, 1.8 Hz, 1H), 8.14 (ap dd, *J* = 8.9, 5.4 Hz, 2H), 7.68 (td, *J* = 7.7, 1.8 Hz, 1H), 7.44–7.51 (m, 3H), 7.15–7.22 (m, 3H), 5.18 (s, 2H), 1.38 (s, 9H). LRMS [M + H] = 497.2.

Deprotection of **Boc-39** (0.058 g, 0.12 mmol) using the general deprotection procedure gave **39** (0.038 g, 82%) as yellow microcrystals. Purity (HPLC) 99.9%; mp 198–201 °C. ^1^H NMR (CDCl_3_) δ 9.32 (dd, *J* = 2.2, 0.6 Hz, 1H), 8.69–8.71 (m, 2H), 8.47 (dt, *J* = 8.0, 1.8 Hz, 1H), 8.37 (s, 1H), 8.15 (ap dd, *J* = 8.9, 5.4 Hz, 2H), 7.75 (td, *J* = 7.7, 1.8 Hz, 1H), 7.71 (t, *J* = 5.7 Hz, 1H), 7.46 (ddd, *J* = 8.0, 4.8, 0.7 Hz, 1H), 7.40 (d, *J* = 7.8 Hz, 1H), 7.30 (dd, *J* = 6.6, 4.9 Hz, 1H), 7.16 (ap t, *J* = 8.8 Hz, 2H), 6.48 (s, 1H), 4.83 (d, *J* = 5.2 Hz, 2H). ^13^C NMR (CDCl_3_) δ 162.65, 160.22, 154.79, 154.72, 150.77, 149.81, 148.83, 147.11, 145.36, 142.02, 137.29, 135.01, 134.43, 129.04, 129.01, 127.64, 127.57, 123.77, 123.22, 121.71, 115.85, 115.64, 109.40, 82.89, 46.94. HRMS calcd. for C_23_H_18_FN_6_ (M + H^+^) *m/z* 396.1577, found 396.1579.

3-(4-Fluorophenyl)-*N*-(pyridin-2-ylmethyl)-5-(pyridin-4-yl)pyrazolo[1,5-*a*]pyrimidin-7-amine (**40**)

Reaction of **76** (0.100 g, 0.22 mmol) and pyridin-4-ylboronic acid (0.108 g, 0.88 mmol) using the general Suzuki procedure gave **Boc-40** (0.066 g, 60%) as a yellow solid. ^1^H NMR (CDCl_3_) δ 8.81 (dd, *J* = 4.6, 1.7 Hz, 2H), 8.55 (bd, *J* = 4.1 Hz, 1H), 8.47 (s, 1H), 8.13 (ap dd, *J* = 8.9, 5.4 Hz, 2H), 7.98 (dd, *J* = 4.5, 1.6 Hz, 2H), 7.68 (td, *J* = 7.7, 1.8 Hz, 1H), 7.53 (s, 1H), 7.49 (d, *J* = 7.8 Hz, 1H), 7.17–7.23 (m, 3H), 5.19 (s, 2H), 1.38 (s, 9H). LRMS [M + H] = 497.3.

Deprotection of **Boc-40** (0.075 g, 0.15 mmol) using the general deprotection procedure gave **40** (0.045 g, 75%) as orange microcrystals. Purity (HPLC) 99.9%; mp 250–253 °C. ^1^H NMR (CDCl_3_) δ 8.77 (dd, *J* = 4.5, 1.6 Hz, 2H), 8.70 (ddd, *J* = 4.9, 1.5, 0.6 Hz, 1H), 8.39 (s, 1H), 8.15 (ap dd, *J* = 8.9, 5.4 Hz, 2H), 8.00 (dd, *J* = 4.5, 1.6 Hz, 2H), 7.71–7.77 (m, 2H), 7.39 (d, *J* = 7.8 Hz, 1H), 7.30 (dd, *J* = 7.1, 5.5 Hz, 1H), 7.17 (ap t, *J* = 8.8 Hz, 2H), 6.51 (s, 1H), 4.84 (d, *J* = 5.2 Hz, 2H). HRMS calcd. for C_23_H_18_FN_6_ (M + H^+^) *m/z* 397.1577, found 397.1586.

3-(4-Fluorophenyl)-*N*-(pyridin-2-ylmethyl)-5-(thiophen-2-yl)pyrazolo[1,5-*a*]pyrimidin-7-amine (**41**)

Reaction of **76** (0.100 g, 0.22 mmol) and thiophen-2-ylboronic acid (0.452 g, 3.53 mmol; added in 0.113 g and 0.339 g portions) using the general Suzuki procedure gave **Boc-41** (0.039 g, 35%) as a yellow solid. ^1^H NMR (CDCl_3_) δ 8.55 (ddd, *J* = 4.8, 1.6, 0.9 Hz, 1H), 8.38 (s, 1H), 8.12 (ap dd, *J* = 9.0, 5.4 Hz, 2H), 7.67 (td, *J* = 7.7, 1.8 Hz, 1H), 7.63 (dd, *J* = 3.7, 1.1 Hz, 1H), 7.52 (dd, *J* = 5.3, 1.1 Hz, 1H), 7.50 d (*J* = 7.9 Hz, 1H), 7.28 (s, 1H), 7.13–7.19 (m, 4H), 5.16 (s, 2H), 1.37 (s, 9H). LRMS [M + H] = 502.2.

Deprotection of **Boc-41** (0.042 g, 0.084 mmol) using the general deprotection procedure gave **41** (0.032 g, 95%) as a yellow solid. Purity (HPLC) 97.9%; mp 181–183 °C. ^1^H NMR (CDCl_3_) δ 8.69 (dd, *J* = 4.8, 0.6 Hz, 1H), 8.32 (s, 1H), 8.15 (ap dd, *J* = 9.0, 5.4 Hz, 2H), 7.72 (td, *J* = 7.7, 1.8 Hz, 1H), 7.66 (dd, *J* = 3.7, 1.1 Hz, 1H), 7.55 (t, *J* = 5.1 Hz, 1H), 7.47 (dd, *J* = 5.0, 1.1 Hz, 1H), 7.38 (d, *J* = 7.8 Hz, 1H), 7.28 (dd, *J* = 7.2, 5.6, 1H), 7.11–7.18 (m, 3H), 6.37 (s, 1H), 4.80 (d, *J* = 5.3 Hz, 2H). ^13^C NMR (CDCl_3_) δ 162.54, 160.11, 154.96, 152.42, 149.75, 146.76, 145.04, 144.80, 141.73, 137.27, 129.22, 129.19, 129.14, 128.15, 127.48, 127.40, 126.32, 123.14, 121.65, 115.75, 115.54, 108.72, 81.86, 77.43, 46.95, 45.78. HRMS calcd. for C_22_H_17_FN_5_S (M + H^+^) *m/z* 402.1183, found 402.1189.

3-(4-Fluorophenyl)-*N*-(pyridin-2-ylmethyl)-5-(thiophen-3-yl)pyrazolo[1,5-*a*]pyrimidin-7-amine (**42**)

Reaction of **76** (0.100 g, 0.22 mmol) and thiophen-3-ylboronic acid (0.113 g, 0.88 mmol) using the general Suzuki procedure gave **Boc-42** (0.078 g, 71%) as a yellow solid. ^1^H NMR (CDCl_3_) δ 8.55 (dd, *J* = 4.8, 1.7, 0.9 Hz, 1H), 8.38 (s, 1H), 8.10 ( ap dd, *J* = 8.9, 5.4 Hz, 2H), 8.07 (dd, *J* = 1.4, 0.8 Hz, 1H), 7.67 (td, *J* = 7.7, 1.8 Hz, 1H), 7.53 (t, *J* = 1.7 Hz, 1H), 7.49 (d, *J* = 7.9 Hz, 1H), 7.12–7.20 (m, 3H), 7.09 (s, 1H), 7.02 (dd, *J* = 1.8, 0.8 Hz, 1H), 5.15 (s, 2H), 1.37 (s, 9H). LRMS [M + H] = 502.2.

Deprotection of **Boc-42** (0.073 g, 0.15 mmol) using the general deprotection procedure gave **42** (0.050 g, 86%) as cream microcrystals. Purity (HPLC) 99.9%; mp 155–157 °C. ^1^H NMR (CDCl_3_) δ 8.69 (ddd, *J* = 4.9, 1.6, 0.9 Hz, 1H), 8.33 (s, 1H), 8.15 (ap dd, *J* = 8.9, 5.4 Hz, 2H), 8.00 (dd, *J* = 3.0, 1.3 Hz, 1H), 7.79 (dd, *J* = 5.0, 1.2 Hz, 1H), 7.73 (td, *J* = 7.7, 1.8 Hz, 1H), 7.53 (t, *J* = 5.2 Hz, 1H), 7.41 (dd, *J* = 5.0, 3.0 Hz, 1H), 7.38 (d, *J* = 7.8 Hz, 1H), 7.28 (dd, *J* = 7.4, 5.8 Hz, 1H), 7.14 (ap t, *J* = 8.8 Hz, 2H), 6.35 (s, 1H), 4.80 (d, *J* = 5.3 Hz, 2H). ^13^C NMR (CDCl_3_) δ 159.98, 154.98, 153.26, 149.66, 145.27, 141.97, 141.64, 137.14, 129.24, 129.21, 127.43, 126.90, 126.28, 125.23, 123.01, 121.51, 115.63, 115.41, 108.67, 83.17, 46.89. HRMS calcd. for C_22_H_17_FN_5_S (M + H^+^) *m/z* 402.1183, found 402.1183.

3-(4-Fluorophenyl)-5-(furan-2-yl)-*N*-(pyridin-2-ylmethyl)pyrazolo[1,5-*a*]pyrimidin-7-amine (**43**)

Reaction of **76** (0.100g, 0.22 mmol) and furan-2-ylboronic acid (0.210 g, 1.77 mmol) using the general Suzuki procedure gave **Boc-43** (0.045 g, 42%) as an orange solid. ^1^H NMR (CDCl_3_) δ ^1^H NMR (CDCl_3_) δ 8.54 (ddd, *J* = 4.9, 1.7, 0.7 Hz, 1H), 8.38 (s, 1H), 8.10 (ap dd, *J* = 8.9, 5.4 Hz, 2H), 8.07 (dd, *J* = 1.4, 0.8 Hz, 1H), 7.68 (td, *J* = 7.7, 1.8 Hz, 1H), 7.53 (t, *J* = 1.6 Hz, 1H), 7.50 (d, *J* = 7.9 Hz, 1H), 7.12–7.20 (m, 3H), 7.09 (s, 1H), 7.02 (dd, *J* = 1.9, 0.8 Hz, 1H), 5.15 (s, 2H), 1.37 (s, 9H). LRMS [M + H] = 486.2.

Deprotection of **Boc-43** (0.038 g, 0.078 mmol) using the general deprotection procedure gave **43** (0.013 g, 43%) as a tan solid. Purity (HPLC) 99.9%; 8.68 (ddd, *J* = 4.9, 1.6, 0.9 Hz, 1H), 8.33 (s, 1H), 8.12 (ap dd, *J* = 9.0, 5.5 Hz, 2H), 7.73 (td, *J* = 7.7, 1.8 Hz, 1H), 7.60 (bt, *J* = 5.0 Hz, 1H), 7.57 (dd, *J* = 1.7, 0.8 Hz, 1H), 7.38 (d, *J* = 7.8 Hz, 1H), 7.27–7.30 (m, 2H), 7.14 (ap t, *J* = 8.8 Hz, 2H), 6.58 (dd, *J* = 3.4, 1.8 Hz, 1H), 6.51 (s, 1H), 4.81 (d, *J* = 5.2 Hz, 2H). HRMS calcd. for C_22_H_17_FN_5_O (M + H^+^) *m/z* 386.1412, found 386.1412.

3-(4-Fluorophenyl)-5-(furan-3-yl)-*N*-(pyridin-2-ylmethyl)pyrazolo[1,5-*a*]pyrimidin-7-amine (**44**)

Reaction of **76** (0.300 g, 0.66 mmol) and furan-3-ylboronic acid (0.297 g, 2.66 mmol) using the general Suzuki procedure gave crude **Boc-44** (0.075 g, 23%), which was used directly in the subsequent step. LRMS [M + H] = 486.2.

Deprotection of **Boc-44** (0.069 g, 0.14 mmol) using the general deprotection procedure gave **44** (0.046 g, 84%) as a yellow solid. Purity (HPLC) 98.5%; mp 151–153 °C. ^1^H NMR (CDCl_3_) δ 8.69 (ddd, *J* = 4.9, 1.6, 0.9 Hz, 1H), 8.32 (s, 1H), 8.13 (ap dd, *J* = 9.0, 5.4 Hz, 2H), 8.09 (dd, *J* = 1.4, 0.8 Hz, 1H), 7.73 (td, *J* = 7.7, 1.8 Hz, 1H), 7.53 (t, *J* = 5.1 Hz, 1H), 7.52 (dd, *J* = 3.4, 1.7 Hz, 1H), 7.38 (d, *J* = 7.8 Hz, 1H), 7.28 (dd, *J* = 7.4, 5.7 Hz, 1H), 7.13 (ap t, *J* = 8.8 Hz, 2H), 7.01 (dd, *J* = 1.8, 0.8 Hz, 1H), 6.18 (s, 1H), 5.30 (d, *J* = 4.8 Hz, 2H). ^13^C NMR (CDCl_3_) δ 162.54, 160.11, 155.07, 151.98, 149.79, 146.83, 145.44, 144.08, 142.60, 141.72, 137.29, 129.31, 129.28, 127.55, 127.47, 127.28, 123.16, 121.63, 115.74, 115.53, 109.34, 108.59, 83.12, 47.00. HRMS calcd. for C_22_H_17_FN_5_O (M + H^+^) *m/z* 386.1412, found 386.1410.

Preparation of compounds **45**–**72** of Table 3:


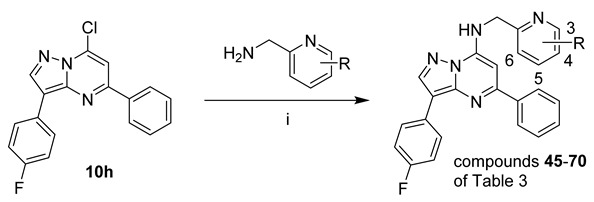


Reagents and conditions: (i) DIPEA, iPrOH, reflux, 3–15h.

3-(4-Fluorophenyl)*-N-*((6-fluoropyridin-2-yl)methyl)-5-phenylpyrazolo[1,5-*a*]pyrimidin-7-amine (**45**) 

A mixture of **10h** (0.046 g, 0.14 mmol), 6-fluoropyridine-2-methanamine hydrochloride (0.0277 mg, 0.170 mmol) and diisopropylethylamine (0.12 mL, 0.71 mmol) was heated to 110 °C for 16 h. The reaction mixture was partitioned between water and ethyl acetate. The aqueous fraction was extracted twice with ethyl acetate, and the organic fraction was combined, dried (MgSO_4_) and concentrated in *vacuo*. Column chromatography on silica gel using a gradient of 5:1 hexanes:ethyl acetate to 3:1 hexanes:ethyl acetate gave compound **45** (0.054 g, 92%) as a yellow solid. Purity (HPLC) 99.1%; mp 196–198 °C. ^1^H NMR (CDCl_3_) δ 8.34 (s, 1H), 8.20–8.13 (m, 2H), 8.12–8.08 (m, 2H), 7.82 (q, *J* = 7.9 Hz, 1H), 7.52–7.46 (m, 3H), 7.29 (dd, *J* = 7.4, 1.7 Hz, 1H), 7.21–7.11 (m, 3H), 6.92 (dd, *J* = 8.2, 2.2 Hz, 1H), 6.45 (s, 1H), 4.80 (d, *J* = 6.0 Hz, 2H). HRMS calcd. for C_24_H_18_F_2_N_5_ (M + H^+^) *m/z* 414.1525, found 414.1516.

*N*-((6-Bromopyridin-2-yl)methyl)-3-(4-fluorophenyl)-5-phenylpyrazolo[1,5-*a*]pyrimidin-7-amine (**46**) 

Similar reaction of **10h** (0.0392 g, 0.121 mmol) with (6-bromopyridin-2-yl)methanamine (0.0272 g, 0.145 mmol) gave compound **46** (0.047 g, 82%). Purity (HPLC) 94.9%. mp 160–161 °C. ^1^H NMR (CDCl_3_), δ 8.34 (s, 1H), 8.21–8.14 (m, 2H), 8.13–8.08 (m, 2H), 7.60–7.54 (m, 1H), 7.54–7.46 (m, 4H), 7.36 (d, *J* = 7.5 Hz, 1H), 7.20–7.13 (m, 3H), 6.48 (s, 1H), 4.83 (d, *J* = 6.0 Hz, 2H). HRMS calcd. for C_24_H_17_BrFN_5_: 473.0651, found 473.0679.

3-(4-Fluorophenyl)*-N-*((6-methylpyridin-2-yl)methyl)-5-phenylpyrazolo[1,5-*a*]pyrimidin-7-amine (**47**) 

Similar reaction of **10h** (0.0592 g, 0.183 mmol) with (6-methylpyridin-2-yl)methanamine (0.0268 g, 0.219 mmol) gave compound **47** (0.048 g, 64%). Purity (HPLC) 99.1%; mp 177–180 °C. ^1^H NMR (CDCl_3_), δ 8.35 (s, 1H), 8.20–8.11 (m, 4H), 7.60 (t, *J* = 7.7 Hz, 1H), 7.53–7.44 (m, 4H), 7.19–7.10 (m, 4H), 6.50 (s, 1H), 4.78 (d, *J* = 5.4 Hz, 2H). 2.64 (s, 3H). HRMS calcd. for C_25_H_20_FN_5_: 409.1703, found 409.1718.

3-(4-Fluorophenyl)*-N-*((6-methoxypyridin-2-yl)methyl)-5-phenylpyrazolo[1,5-*a*]pyrimidin-7-amine (**48**) 

Similar reaction of **10h** (0.0440 g, 0.136 mmol) with (6-methoxypyridin-2-yl)methanamine (0.0265 g, 0.163 mmol) gave compound **48** (0.052 g, 90%). Purity (HPLC) 99.6%; mp 163–165 °C. ^1^H NMR (CDCl_3_), δ 8.37 (s, 1H), 8.19–8.10 (m, 4H), 7.60–7.56 (m, 1H), 7.53–7.40 (m, 4H), 7.17–7.11 (m, 2H), 6.92 (d, *J* = 7.2 Hz, 1H), 6.69 (d, *J* = 8.2 Hz, 1H), 6.51 (s, 1H), 4.71 (d, *J* = 5.5 Hz, 2H). 4.00 (s, 3H). HRMS calcd. for [M + H]^+^ = C_25_H_21_FN_5_O: 426.1725, found 426.1711.

*N*-((6-(Dimethylamino)pyridin-2-yl)methyl)-3-(4-fluorophenyl)-5-phenylpyrazolo[1,5-*a*]pyrimidin-7-amine (**49**)

Similar reaction of **10h** (0.21 g, 0.64 mmol) with 6-(aminomethyl)-*N*,*N*-dimethylpyridin-2-amine dihydrochloride (0.17 g, 0.77 mmol) gave compound **49** (0.21 g, 75%). Purity (HPLC) 98.0%; mp 151–153 °C. ^1^H NMR (CDCl_3_), δ 8.41 (dd, *J* = 4.5, 1.4 Hz, 1H), 8.36 (s, 1H), 8.21–8.16 (m, 4H), 8.08 (br t, *J* = 4.6 Hz, 1H), 7.56–7.48 (m, 4H), 7.27 (m, 1H), 7.15 (t, *J* = 8.9 Hz, 2H), 6.58 (s, 1H), 4.79 (d, *J* = 4.7 Hz, 2H), 2.80 (s, 6H). HRMS calcd. for C_26_H_24_FN_6_ (M + H^+^) *m/z* 439.2041, found 439.2045.

3-(4-Fluorophenyl)-5-phenyl*-N-*((6-(piperidin-1-yl)pyridin-2-yl)methyl)pyrazolo[1,5-*a*]pyrimidin-7-amine (**50**)

Similar reaction of **10h** (0.20 g, 0.62 mmol) with (6-(piperidin-1-yl)pyridin-2-yl)methanamine dihydrochloride (0.19 g, 0.74 mmol) gave compound **50** (0.25 g, 83%). Purity (HPLC) 98.0%; mp 185–187 °C. ^1^H NMR (CDCl_3_) δ 8.39 (dd, *J* = 4.7, 1.4 Hz, 1H), 8.35 (s, 1H), 8.20–8.15 (m, 4H), 8.05 (br t, *J* = 4.9 Hz, 1H), 7.56–7.48 (m, 4H), 7.27 (m, 1H), 7.14 (t, *J* = 8.9 Hz, 2H), 6.56 (s, 1H), 4.80 (d, *J* = 5.0 Hz, 2H), 2.91 (br t, *J =* 5.2 Hz, 4H), 1.85 (m, 4H), 1.66 (m, 2H). HRMS calcd. for C_29_H_28_FN_6_ (M + H^+^) *m/z* 479.2354, found 479.2361.

3-(4-Fluorophenyl)*-N-*((6-(4-methylpiperidin-1-yl)pyridin-2-yl)methyl)-5-phenylpyrazolo[1,5-*a*]pyrimidin-7-amine (**51**)

Similar reaction of **10h** (0.20 g, 0.62 mmol) with (6-(4-methylpiperazin-1-yl)pyridin-2-yl)methanamine dihydrochloride (0.21 g, 0.74 mmol) gave compound **51** (0.14 g, 45%). Purity (HPLC) 98.0%; mp 183–185 °C. ^1^H NMR (CDCl_3_) δ 8.43 (dd, *J =* 4.7, 1.4 Hz, 1H), 8.34 (s, 1H), 8.20–8.15 (m, 4H), 8.04 (br t, *J =* 4.9 Hz, 1H), 7.59–7.48 (m, 4H), 7.29 (m, 1H), 7.15 (t, *J* = 8.8 Hz, 2H), 6.55 (s, 1H), 4.80 (d, *J* = 5.0 Hz, 2H), 3.07 (t, *J* = 4.9 Hz, 4H), 2.78 (m, 4H), 2.47 (s, 3H). HRMS calcd. for C_29_H_29_FN_7_ (M + H^+^) *m/z* 494.2463, found 494.2463.

3-(4-Fluorophenyl)*-N-*((5-fluoropyridin-2-yl)methyl)-5-phenylpyrazolo[1,5-*a*]pyrimidin-7-amine (**52**)

Similar reaction of **10h** (0.0475 g, 0.147 mmol) with (5-fluoropyridin-2-yl)methanamine (0.0222 g, 0.176 mmol) gave compound **52** (0.050 g, 82%). Purity (HPLC) 99.5%; mp 118–120 °C. ^1^H NMR (CDCl_3_), δ 8.54 (d, *J* = 2.7 Hz, 1H), 8.34 (s, 1H), 8.20–8.09 (m, 4H), 7.52–7.38 (m, 6H), 7.19–7.11 (m, 2H), 6.48 (s, 1H), 4.82 (d, *J* = 5.4 Hz, 2H). ^13^C NMR (CDCl_3_) δ 162.59, 160.48, 160.17, 157.93, 157.60, 151.17, 151.13, 146.87, 145.47, 141.85, 138.83, 138.17, 137.93, 130.12, 129.26, 129.22, 128.92, 127.64, 127.57, 124.33, 124.14, 122.55, 122.50, 115.81, 115.59, 109.17, 83.27, 16.57, 16.56, 0.21. HRMS calcd. for C_24_H_17_F_2_N_5_: 413.1452, found 413.1464.

*N*-((5-Chloropyridin-2-yl)methyl)-3-(4-fluorophenyl)-5-phenylpyrazolo[1,5-*a*]pyrimidin-7-amine (**53**) 

Similar reaction of **10h** (0.0475 g, 0.147 mmol) with (4-chloropyridin-2-yl)methanamine (0.0222 g, 0.176 mmol) gave compound **53** (0.053 g, 86%). Purity (HPLC) 99.5%; mp 139–141 °C. ^1^H NMR (CDCl_3_) δ 8.63 (d, *J* = 2.0 Hz, 1H), 8.37 (s, 1H), 8.20–8.09 (m, 4H), 7.72 (d, *J* = 8.4, 2.5 Hz, 1H), 7.53–7.47 (m, 3H), 7.41 (t, *J* = 5.5 Hz, 1H), 7.36 (d, *J* = 8.3 Hz, 1H), 7.19–7.13 (m, 2H), 6.46 (s, 1H), 4.82 (d, *J* = 5.5 Hz, 2H). ^13^C NMR (CDCl_3_) δ 162.60, 160.17, 157.58, 153.47, 148.73, 146.83, 145.45, 141.86, 138.79, 137.07, 131.61, 130.13, 129.23, 129.20, 128.92, 127.64, 127.57, 122.32, 115.81, 115.59, 109.18, 83.25, 46.60. HRMS calcd. for C_24_H_17_ClFN_5_: 429.1157, found 429.1158.

3-(4-Fluorophenyl)*-N-*((5-methoxypyridin-2-yl)methyl)-5-phenylpyrazolo[1,5-*a*]pyrimidin-7-amine (**54**) 

Similar reaction of **10h** (0.0625 g, 0.193 mmol) with (4-methoxypyridin-2-yl)methanamine (0.0320 mg, 0.232 mmol) gave compound **54** (0.028 g, 34%). Purity (HPLC) 98.8%; mp 212–214 °C. ^1^H NMR (CDCl_3_) δ 8.36 (d, *J* = 2.8 Hz, 1H), 8.34 (s, 1H), 8.20–8.11 (m, 4H), 7.54–7.48 (m, 3H), 7.40 (t, *J* = 5.4Hz, 1H), 7.31 (d, *J* = 8.6 Hz, 1H), 7.24–7.21 (m, 1H), 7.18–7.12 (m, 2H), 6.49 (s, 1H), 4.77 (d, *J* = 5.4 Hz, 2H). ^13^C NMR (CDCl_3_) δ 162.54, 160.11, 157.54, 155.44, 147.10, 146.98, 145.49, 141.75, 138.92, 137.15, 130.03, 129.36, 129.33, 128.88, 127.58, 127.51, 121.99, 121.93, 115.77, 115.56, 108.99, 83.29, 55.93, 46.60. HRMS calcd. for C_25_H_20_FN_5_O: 425.1652, found 425.1657.

*N*-((5-(3-(Dimethylamino)prop-1-yn-1-yl)pyridin-2-yl)methyl)-3-(4-fluorophenyl)-5-phenylpyrazolo[1,5-*a*]pyrimidin-7-amine (**55**) and *N*-((5-(3-(dimethylamino)propyl)pyridin-2-yl)methyl)-3-(4-fluorophenyl)-5-phenylpyrazolo[1,5-*a*]pyrimidin-7-amine (**56**)


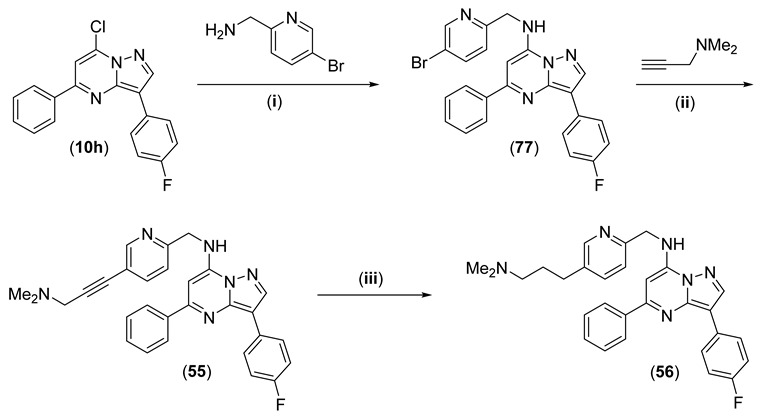


From **10h** (0.803 g, 2.48 mmol) and (5-bromopyridin-2-yl)methanamine (0.635 g, 3.40 mmol), **77** (1.165 g, 99%) was synthesised using the general procedure, 19 h reflux. ^1^H NMR (CDCl_3_) δ 8.74 (d, *J* = 1.9 Hz, 1H), 8.35 (s, 1H), 8.17 (ap dd, *J* = 8.9, 5.4 Hz, 2H), 8.11 (ap dd, *J* = 5.9, 1.4 Hz, 2H), 7.85 (dd, *J* = 8.3, 2.4 Hz, 1H), 7.45–7.55 (m, 3H), 7.40 (t, *J* = 5.4 Hz, 1H), 7.30 (d, *J* = 8.3 Hz, 1H), 7.15 (ap t, *J* = 8.8 Hz, 2H), 6.45 (s, 1H), 4.79 (d, *J* = 5.5 Hz, 2H). LRMS [M + H] = 475.2.

A mixture of **77** (0.200 g, 0.422 mmol), PdCl_2_(PPh_3_)_2_ (0.030 g, 0.043 mmol) and CuI (4 mg, 0.021 mmol) in anhydrous DMF (1 mL) and triethylamine (1 mL) was purged with nitrogen in a sealable tube. *N*,*N*-Dimethylprop-2-yn-1-amine (0.23 mL, 2.1 mmol) was added, and the mixture was sealed then heated to 65 °C under nitrogen for 15 h. The mixture was partitioned between EtOAc and water, the organic fractions were dried (MgSO_4_) and evaporated on to silica gel. Column chromatography using a gradient of EtOAc to 95:5 EtOAc:MeOH with 0.75% aq. ammonia gave **55** (0.192 g, 95%) as yellow microcrystals. Purity (HPLC) 98.3%; mp 182–184 °C. ^1^H NMR (CDCl_3_) δ 8.72 (d, *J* = 1.4 Hz, 1H), 8.35 (s, 1H), 8.17 (ap dd, *J* = 8.9, 5.4 Hz, 2H), 8.12 (ap dd, *J* = 0.3, 1.7 Hz, 2H), 7.75 (dd, *J* = 8.1, 2.1 Hz, 1H), 7.44–7.54 (m, 4H), 7.33 (d, *J* = 8.1 Hz, 1H), 7.15 (ap t, *J* = 8.8 Hz, 2H), 6.46 (s, 1H), 4.82 (d, *J* = 5.4 Hz, 2H), 3.49 (s, 2H), 2.38 (s, 6H). ^13^C NMR (CDCl_3_) δ 162.50, 160.07, 157.50, 154.07, 152.26, 146.81, 145.39, 141.77, 139.78, 138.75, 130.12, 129.19, 129.16, 128.83, 127.55, 127.50, 127.48, 120.82, 119.71, 115.71, 115.50, 109.06, 88.75, 83.20, 81.75, 48.66, 46.88, 44.42. HRMS calcd. for C_29_H_26_FN_6_ (M + H^+^) *m/z* 477.2197, found 477.2196.

A solution of **55** (0.079 g, 0.166 mmol) in EtOH (20 mL) was purged with nitrogen in a hydrogenation bottle, 5% Pd/C (39 mg) was added, and the mixture was hydrogenated at 60 psi for 18 h. The mixture was filtered through celite and evaporated. Column chromatography using a gradient of EtOAc to 95:5 EtOAc:MeOH with 0.75% aq. ammonia gave **56** (0.020 g, 25%) as pale yellow microcrystals. Purity (HPLC) 96.0%; mp 151–153 °C. ^1^H NMR (CDCl_3_) δ 8.52 (d, *J* = 1.8 Hz, 1H), 8.35 (s, 1H), 8.18 (ap dd, *J* = 8.9, 5.5 Hz, 2H), 8.13 (ap dd, *J* = 8.0, 1.3 Hz, 2H), 7.45–7.58 (m, 5H), 7.30 (d, *J* = 7.8 Hz, 1H), 7.15 (ap t, *J* = 8.8 Hz, 2H), 6.49 (s, 1H), 4.79 (d, *J* = 5.3 Hz, 2H), 2.68 (t, *J* = 7.6 Hz, 2H), 2.30 (t, *J* = 7.1 Hz, 2H), 2.23 (s, 6H), 1.76–1.84 (m, 2H). HRMS calcd. for C_29_H_30_FN_6_ (M + H^+^) *m/z* 481.2516, found 481.2511.

*N*-((5-(2-(Dimethylamino)ethoxy)pyridin-2-yl)methyl)-3-(4-fluorophenyl)-5-phenylpyrazolo[1,5-*a*]pyrimidin-7-amine (**57**)


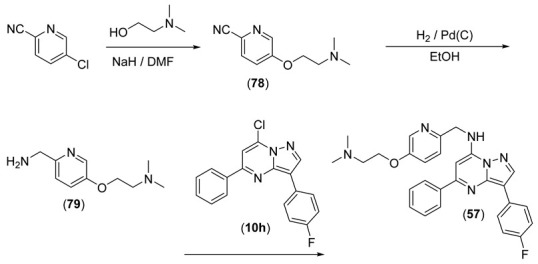


To a slurry of NaH (60% *w*/*w*, 1.04 g, 26.0 mmol) in anhydrous DMF (8 mL), 2-(dimethylamino)ethan-1-ol (2.18 mL, 19.4 mmol) was added, and this mixture was stirred for 15 min and then 5-chloropicolinonitrile (2.00 g, 14.4 mmol) and anhydrous DMF (8 mL) were added. The mixture was stirred for 2 h, then partitioned between EtOAc and water, and the organic fractions were dried and evaporated on to silica gel. Column chromatography using a gradient of 5–7.5% MeOH:EtOAc gave **78** (1.333 g, 48%) as a faint pinkish solid. ^1^H NMR (CDCl_3_) δ 8.40 (d, *J* = 2.7 Hz, 1H), 7.64 (dd, *J* = 8.6, 0.2 Hz, 1H), 7.27 (dd, *J* = 8.6, 2.9 Hz, 1H), 4.16 (t, *J* = 5.6 Hz, 2H), 2.77 (t, *J* = 5.6 Hz, 2H), 2.34 (s, 6H). LRMS [M + H] = 192.2.

A solution of **78** (0.848 g, 4.43 mmol) in MeOH (20 mL) was purged with nitrogen in a hydrogenation bottle, 5% Pd/C (85 mg) was added, and the mixture was hydrogenated at 50 psi for 16 h. The mixture was filtered through celite and evaporated to give crude **79,** which was used without further purification. ^1^H NMR (CDCl_3_) δ 8.27 (d, *J* = 2.6 Hz, 1H), 7.26 (d, *J* = 8.7 Hz, 1H), 7.18 (dd, *J* = 8.7, 2.9 Hz, 1H), 4.08–4.11 (m, 2H), 3.88 (s, 2H), 2.72–2.75 (m, 2H), 2.34 (s, 6H), 2H exchanged. LRMS [M + H] = 196.2.

Using the general procedure, **57** (0.107 g, 28%) was synthesised as yellow microcrystals from **10h** (0.260 g, 0.803 mmol) and **79** (0.283 g, 1.45 mmol). Purity (HPLC) 99.1%; mp 130–132 °C. ^1^H NMR (CDCl_3_) δ 8.38 (d, *J* = 2.4 Hz, 1H), 8.34 (s, 1H), 8.18 (ap, dd, *J* = 8.9, 5.4 Hz, 2H), 8.13 (ap dd, *J* = 7.9, 1.2 Hz, 2H), 7.44–7.55 (m, 3H), 7.40 (t, *J* = 5.2 Hz, 1H), 7.24–7.32 (m, 2H), 7.13 (ap t, *J* = 8.8 Hz, 2H), 6.49 (s, 1H), 4.76 (d, *J* = 5.4 Hz, 2H), 4.12 (t, *J* = 5.6 Hz, 2H), 2.76 (t, *J* = 5.6 Hz, 2H), 2.35 (s, 6H). HRMS calcd. for C_28_H_27_FN_6_NaO (M + Na^+^) *m/z* 505.2123, found 505.2121.

*N*-((4-(2-(Dimethylamino)ethoxy)pyridin-2-yl)methyl)-3-(4-fluorophenyl)-5-phenylpyrazolo[1,5-*a*]pyrimidin-7-amine (**58**)


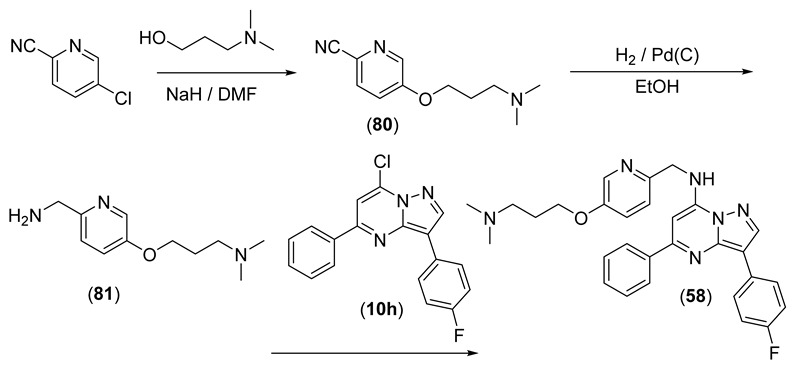


Reaction of 5-chloropicolinonitrile (2.00 g, 1.44 mmol) and 3-(dimethylamino)propan-1-ol (2.6 mL, 2.2 mmol) gave **80** (2.249 g, 76%) as a pinkish solid. ^1^H NMR (CDCl_3_) δ 8.37 (d, *J* = 2.6 Hz, 1H), 7.63 (dd, *J* = 8.6, 0.4 Hz, 1H), 7.24 (dd, *J* = 8.6, 2.9 Hz, 1H), 4.13 (t, *J* = 6.4 Hz, 2H), 2.45 (t, *J* = 7.0 Hz, 2H), 2.25 (s, 6H), 1.96–2.03 (m, 2H). LRMS [M + H] = 206.2.

Hydrogenation of **80** (2.028 g, 9.88 mmol) gave **81** (1.94 g, 94%), which was used directly without further purification. ^1^H NMR (CDCl_3_) δ 8.24 (d, *J* = 2.6 Hz, 1H), 7.26 (d, *J* = 8.5 Hz, 1H), 7.16 (d, *J* = 8.5, 2.9 Hz, 1H), 4.03–4.13 (m, 2H), 3.88 (s, 2H), 2.45 (t, *J* = 7.0 Hz, 2H), 2.25 (s, 6H), 1.94–2.00 (m, 2H). LRMS [M + H] = 210.2

Using the general procedure, **58** (0.099 g, 32%) was synthesised as yellow microcrystals from **10h** (0.200 g, 0.618 mmol) and **81** (0.259 g, 1.24 mmol). Purity (HPLC) 96.0%; mp 151–153 °C. ^1^H NMR (CDCl_3_) δ 8.34 (d, *J* = 3.2 Hz, 1H), 8.34 (s, 1H), 8.18 (ap dd, *J* = 8.9, 5.4 Hz, 2H), 8.13 (ap dd, *J* = 7.9, 2.6 Hz, 2H), 7.45–7.55 (m, 3H), 7.39 (t, *J* = 5.3 Hz, 1H), 7.29 (d, *J* = 8.6 Hz, 1H), 7.23 (dd, *J* = 8.6, 2.8 Hz, 1H), 7.15 (ap t, *J* = 8.8 Hz, 2H), 6.49 (s, 1H), 4.76 (d, *J* = 5.4 Hz, 2H), 4.09 (t, *J* = 6.3 Hz, 2H), 2.52 (t, *J* = 7.1 Hz, 2H), 2.30 (s, 6H), 1.98–2.05 (m, 2H), 2H exchanged. HRMS calcd. for C_29_H_30_FN_6_O (M + H^+^) *m/z* 497.2465, found 497.2470.

3-(4-Fluorophenyl)-5-phenyl*-N-*((5-(piperidin-1-yl)pyridin-2-yl)methyl)pyrazolo[1,5-*a*]pyrimidin-7-amine (**59**) 

Similar reaction of **10h** (0.0196 g, 0.0605 mmol) with (5-(piperidin-1-yl)pyridin-2-yl)methanamine (0.014 g, 0.073 mmol) gave compound **59** (0.016 g, 55%). HPLC = 98.4%. mp 179–182 °C. ^1^H NMR (CDCl_3_, 400 MHz) δ 8.33 (s, 2H), 8.19–8.12 (m, 4H), 7.53–7.47 (m, 3H), 7.37 (t, *J* = 5.5 Hz, 1H), 7.22–7.20 (m, 2H), 7.18–7.11 (m, 2H), 6.51 (s, 1H), 4.72 (d, *J* = 5.3 Hz, 2H), 3.21 (t, *J* = 5.4 Hz, 4H), 1.75–1.69 (m, 4H), 1.63–1.58 (m, 2H). HRMS calcd. for C_29_H_27_FN_6_: 478.2281, found 478.2297.

*N*-((4-Chloropyridin-2-yl)methyl)-3-(4-fluorophenyl)-5-phenylpyrazolo[1,5-*a*]pyrimidin-7-amine (**60**) 

Similar reaction of **10h** (0.0425 g, 0.131 mmol) with (4-chloropyridin-2-yl)methanamine (0.0282, 0.158 mmol) gave compound **60** (0.045 g, 80%). Purity (HPLC = 99.1%); mp 220–222 °C. ^1^H NMR (CDCl_3_, 400 MHz) δ 8.58 (d, *J* = 5.3 Hz, 1H), 8.36 (s, 1H), 8.20–8.15 (m, 2H), 8.14–8.10 (m, 2H), 7.55–7.44 (m, 4H), 7.41 (d, *J* = 1.4 Hz, 1H), 7.30 (dd, *J* = 5.4, 1.9 Hz, 1H), 7.18–7.12 (m, 2H), 6.46 (s, 1H), 4.81 (d, *J* = 5.5 Hz, 2H). HRMS calcd. for C_24_H_17_ClFN_5_: 429.1157, found 429.1168.

2-(((3-(4-Fluorophenyl)-5-phenylpyrazolo[1,5-*a*]pyrimidin-7-yl)amino)methyl)isonicotinonitrile (**61**) 

Similar reaction of **10h** (0.0663 g, 0.205 mmol) with 2-(aminomethyl)isonicotinonitrile (0.0417 g, 0.246 mmol) gave compound **61** (0.032 g, 38%). Purity (HPLC = 96.0%); mp 233–236 °C. ^1^H NMR (CDCl_3_, 400 MHz) δ 8.89 (dd, *J* = 5.0, 0.8 Hz, 1H), 8.37 (s, 1H), 8.20–8.14 (m, 2H), 8.13–8.09 (m, 2H), 7.62 (s, 1H), 7.53–7.42 (m, 5H), 7.20–7.11 (m, 2H), 6.44 (s, 1H), 4.90 (d, *J* = 5.5 Hz, 2H). HRMS calcd. for C_25_H_17_FN_6_: 420.1499, found 420.1508.

3-(4-Fluorophenyl)*-N-*((4-methylpyridin-2-yl)methyl)-5-phenylpyrazolo[1,5-*a*]pyrimidin-7-amine (**62**) 

Similar reaction of **10h** (0.236 g, 0.730 mmol) with (4-methylpyridin-2-yl)methanamine (0.107 g, 0.876 mmol) gave **62** (0.260 g, 87%). Purity (HPLC) 99.1%; mp 157–159 °C. ^1^H NMR (CDCl_3_) δ 8.52 (d, *J* = 5.0 Hz, 1H), 8.35 (s, 1H), 8.20–8.10 (m, 4H), 7.53–7.46 (m, 4H), 7.20–7.11 (m, 3H), 7.09 (d, *J* = 5.0 Hz, 1H), 6.48 (s, 1H), 4.78 (d, *J* = 5.3 Hz, 2H), 2.37 (s, 3H). ^13^C NMR (CDCl_3_) δ 162.55, 160.12, 157.57, 149.47, 148.60, 146.99, 145.50, 141.80, 138.92, 130.04, 129.36, 129.33, 128.89, 127.59, 127.53, 124.18, 122.36, 115.78, 115.56, 109.01, 83.26, 46.99, 21.32. HRMS calcd. for C_23_H_19_FN_8_: 409.1703, found 409.1723.

3-(4-Fluorophenyl)*-N-*((4-methoxypyridin-2-yl)methyl)-5-phenylpyrazolo[1,5-*a*]pyrimidin-7-amine (**63**) 

Similar reaction of **10h** (0.144 g, 0.445 mmol) with 4-methoxypyridin-2-yl)methanamine (0.0933 g, 0.534 mmol) gave **63** (0.166 g, 88%). Purity (HPLC) 98.8%; mp 205–208 °C. ^1^H NMR (CDCl_3_, 400 MHz) δ 8.50 (d, *J* = 5.8 Hz, 1H), 8.35 (s, 1H), 8.20–8.10 (m, 4H), 7.52–7.45 (m, 4H), 7.18–7.11 (m, 2H), 6.89 (d, *J* = 2.4 Hz, 1H), 6.80 (dd, *J* = 5.8, 2.5 Hz, 1H), 6.48 (s, 1H), 4.78 (d, *J* = 5.4 Hz, 2H), 3.85 (s, 3H). ^13^C NMR (CDCl_3_) δ 166.78, 162.54, 160.12, 157.55, 156.89, 151.03, 146.96, 145.49, 141.80, 138.89, 133.28, 132.36, 132.26, 132.16, 132.13, 130.04, 129.34, 129.31, 129.08, 128.88, 128.77, 128.65, 127.60, 127.58, 127.53, 115.77, 115.56, 109.21, 109.02, 107.67, 83.26, 55.50, 47.12. HRMS calcd. for C_25_H_20_FN_5_O: 425.1652, found 425.1659.

3-(4-Fluorophenyl)-5-phenyl*-N-*((4-(pyrrolidin-1-yl)pyridin-2-yl)methyl)pyrazolo[1,5-*a*]pyrimidin-7-amine (**64**) 

Similar reaction of **10h** (0.20 g, 0.62 mmol) with (4-(pyrrolidin-1-yl)pyridin-2-yl)methanamine hydrochloride (0.16 g, 0.74 mmol) gave **64** (0.10 g, 35%). Purity (HPLC) 96.5%; mp 221–223 °C. ^1^H NMR (CDCl_3_) δ 8.34 (s, 1H), 8.23 (d, *J =* 6.0 Hz, 1H), 8.21–8.11 (m, 4H), 7.54–7.43 (m, 4H), 7.14 (br t, *J =* 7.0 Hz, 2H), 6.49 (s, 1H), 6.42 (d, *J =* 2.5 Hz, 1H), 6.34 (dd, *J =* 6.0, 2.5 Hz, 1H), 4.68 (d, *J =* 5.1 Hz, 2H), 3.29 (m, 4H), 2.01 (m, 4H). HRMS calcd. for C_28_H_26_FN_6_ (M + H^+^) *m/z* 465.2197, found 465.2206.

3-(4-Fluorophenyl)*-N-*((4-(4-methylpiperazin-1-yl)pyridin-2-yl)methyl)-5-phenylpyrazolo[1,5-*a*]pyrimidin-7-amine (**65**)

Similar reaction of **10h** (0.20 g, 0.62 mmol) with (4-(4-methylpiperazin-1-yl)pyridin-2-yl)methanamine (0.21 g, 0.74 mmol) gave **65** (0.18 g, 59%). Purity (HPLC) 100%; mp 203–205 °C. ^1^H NMR (CDCl_3_) δ 8.35 (s, 1H), 8.32 (d, *J* = 6.0 Hz, 1H), 8.20–8.11 (m, 4H), 7.53–7.44 (m, 4H), 7.15 (br t, *J* = 7.0 Hz, 2H), 6.73 (d, *J* = 2.5 Hz, 1H), 6.64 (dd, *J* = 6.0, 2.5 Hz, 1H), 6.49 (s, 1H), 4.70 (d, *J* = 5.0 Hz, 2H), 3.36 (t, *J* = 5.1 Hz, 4H), 2.51 (t, *J* = 5.1 Hz, 4H), 2.33 (s, 3H). HRMS calcd. for C_29_H_29_FN_7_ (M + H^+^) *m/z* 494.2463, found 494.2466.

3-(4-Fluorophenyl)*-N-*((4-morpholinopyridin-2-yl)methyl)-5-phenylpyrazolo[1,5-*a*]pyrimidin-7-amine (**66**) 

Similar reaction of **10h** (0.20 g, 0.62 mmol) with (4-morpholinopyridin-2-yl)methanamine (0.20 g, 0.74 mmol) gave **66** (0.19 g, 64%). Purity (HPLC) 99%; mp 208 °C. ^1^H NMR (CDCl_3_) δ 8.35 (s, 1H), 8.35 (d, *J* = 6.0 Hz, 1H), 8.20–8.11 (m, 4H), 7.53–7.44 (m, 4H), 7.16 (br t, *J* = 7.0 Hz, 2H), 6.73 (d, *J* = 2.5 Hz, 1H), 6.64 (dd, *J* = 6.0, 2.5 Hz, 1H), 6.48 (s, 1H), 4.71 (d, *J* = 5.0 Hz, 2H), 3.82 (t, *J* = 4.9 Hz, 4H), 3.28 (t, *J* = 5.1 Hz, 4H). HRMS calcd. for C_28_H_26_FN_6_O (M + H^+^) *m/z* 481.2147, found 481.2153.

*N*1-(2-(((3-(4-Fluorophenyl)-5-phenylpyrazolo[1,5-*a*]pyrimidin-7-yl)amino)methyl)pyridin-4-yl)-*N*1,*N*2,*N*2-trimethylethane-1,2-diamine (**67**)

Similar reaction of **10h** (0.20 g, 0.62 mmol) with *N1*-(2-(aminomethyl)pyridin-4-yl)-*N1*,*N2*,*N2*-trimethylethane-1,2-diamine dihydrochloride (0.21 g, 0.74 mmol) gave **67** (0.21 g, 70%). Purity (HPLC) 96.5%; mp 158–160 °C. ^1^H NMR (CDCl_3_) δ 8.34 (s, 1H), 8.26 (d, *J* = 6.0 Hz, 1H), 8.20–8.11 (m, 4H), 7.53–7.44 (m, 4H), 7.16 (br t, *J* = 6.9 Hz, 2H), 6.55 (d, *J* = 2.5 Hz, 1H), 6.49 (s, 1H), 6.47 (dd, *J* = 6.0, 2.5 Hz, 1H), 4.69 (d, *J* = 5.3 Hz, 2H), 3.46 (t, *J* = 7.3 Hz, 2H), 2.99 (s, 3H), 2.40 (t, *J* = 7.3 Hz, 2H), 2.21 (s, 6H). HRMS calcd. for C_29_H_31_FN_7_ (M + H^+^) *m/z* 496.2631, found 496.2628.

3-(4-Fluorophenyl)-5-phenyl*-N-*((4-(piperidin-1-yl)pyridin-2-yl)methyl)pyrazolo[1,5-*a*]pyrimidin-7-amine (**68**) 

Similar reaction of **10h** (0.0542 mg, 0.167 mmol) with (4-(piperidin-1-yl)pyridin-2-yl)methanamine (0.064 g, 0.34 mmol) gave **68** (0.023 g, 29%). Purity (HPLC) 98.4%; mp 189–191 °C. ^1^H NMR (CDCl_3_) δ 8.34 (s, 1H), 8.28 (d, *J* = 6.1 Hz, 1H), 8.20–8.12 (m, 4H), 7.53–7.44 (m, 4H), 7.18–7.11 (m, 2H), 6.71 (d, *J* = 2.4 Hz, 1H), 6.62 (dd, *J* = 6.1, 2.6 Hz, 1H), 6.49 (s, 1H), 4.68 (d, *J* = 5.2 Hz, 2H), 3.36–3.31 (m, 4H), 1.66–1.60 (m, 6H). ^13^C NMR (CDCl_3_) δ 162.51, 160.08, 157.54, 155.88, 155.64, 149.98, 147.10, 145.52, 141.73, 138.97, 129.99, 129.80, 129.42, 129.39, 129.05, 128.86, 127.79, 127.59, 127.56, 127.49, 124.93, 120.64, 120.59, 115.76, 115.54, 108.87, 107.78, 105.43, 83.32, 47.53, 47.46, 25.28, 24.45. HRMS calcd. for C_29_H_27_FN_6_: 478.2281, found 478.2300.

*N*-((3-(Dimethylamino)pyridin-2-yl)methyl)-3-(4-fluorophenyl)-5-phenylpyrazolo[1,5-*a*]pyrimidin-7-amine (**69**) 

Similar reaction of **10h** (0.0696 g, 0.215 mmol) with 2-(aminomethyl)-*N*,*N*-dimethylpyridin-3-amine (0.039 g, 0.258 mmol) gave **69** (0.058 g, 62%). Purity (HPLC) 98.2%; mp 167–170 °C. ^1^H NMR (CDCl_3_) δ 8.42 (dd, *J* = 4.7, 1.4 Hz, 1H), 8.36 (s, 1H), 8.22–8.16 (m, 4H), 8.08 (t, *J* = 4.6 Hz, 1H), 7.56–7.48 (m, 4H), 7.29–7.27 (m, 1H), 7.16–7.12 (m, 2H), 6.59 (s, 1H), 4.81 (d, *J* = 4.7 Hz, 2H), 2.81 (s, 6H). HRMS calcd. for C_26_H_23_FN_6_: 438.1968, found 438.1985.

3-(4-Fluorophenyl)*-N-*((3-fluoropyridin-2-yl)methyl)-5-phenylpyrazolo[1,5-*a*]pyrimidin-7-amine (**70**) 

Similar reaction of **10h** (0.0815 g, 0.252 mmol) with (3-fluoropyridin-2-yl)methanamine (0.0491 g, 0.302 mmol) gave **70** (0.080 g, 77%). Purity (HPLC) 99.0%; mp 165–168 °C. ^1^H NMR (CDCl_3_,) δ 8.53–8.50 (m, 1H), 8.38 (s, 1H), 8.21–8.15 (m, 4H), 7.75 (t, *J* = 4.8 Hz, 1H), 7.57–7.47 (m, 4H), 7.35 (qui, *J* = 4.4 Hz, 1H), 7.18–7.12 (m, 2H), 6.62 (s, 1H), 4.87 (dd, *J* = 5.0, 1.5 Hz, 2H). HRMS calcd. for C_24_H_17_F_2_N_5_: 413.1452, found 413.1452.

*N*-((3-Bromopyridin-2-yl)methyl)-3-(4-fluorophenyl)-5-phenylpyrazolo[1,5-*a*]pyrimidin-7-amine (**71**) 

Similar reaction of **10h** (0.0652, 0.201 mmol) with (3-bromopyridin-2-yl)methanamine (0.054 g, 0.24 mmol) gave **71** (0.048 g, 50%). Purity (HPLC) 98.9%; mp 189–191 °C. ^1^H NMR (CDCl_3_) δ 8.68 (dd, *J* = 4.7, 1.4 Hz, 1H), 8.38 (s, 1H), 8.23–8.16 (m, 4H), 8.07 (t, *J* = 4.4 Hz, 1H), 7.96 (dd, *J* = 8.0, 1.4 Hz, 1H), 7.58–7.47 (m, 3H), 7.25–7.21 (m, 1H), 7.19–7.12 (m, 2H), 6.60 (s, 1H), 4.82 (d, *J* = 4.6 Hz, 2H). HRMS calcd. for C_24_H_17_BrFN_5_: 473.0651, found 473.0660.

2-(((3-(4-Fluorophenyl)-5-phenylpyrazolo[1,5-*a*]pyrimidin-7-yl)amino)methyl)nicotinonitrile (**72**) 

In DMF (2 mL), **71** (0.051 g, 0.108 mmol), zinc cyanide (0.038 g, 0.323 mmol) and Pd(PPh_3_)_4_ (0.031 g, 0.027 mmol) were purged with N_2_ for 10 min. The reaction was sealed in a sealed tube and heated at 115 °C for 4 h. The reaction mixture was added to water, extracted with EtOAc and was dried over anhydrous sodium sulfate. The solvent was removed to give the crude product, which was purified by silica column chromatography using hexanes:EA (2:1) as eluent to give **72** (0.09 g, 20%). Purity (HPLC) 98.8%; mp 241–244 °C. ^1^H NMR (DMSO) δ 8.79 (dd, *J* = 4.9, 1.6 Hz, 1H), 8.69 (s, 1H), 8.52 (t, *J* = 6.1 Hz, 1H), 8.38 (dd, *J* = 7.8, 1.6 Hz, 1H), 8.33–8.28 (m, 2H), 8.24–8.20 (m, 2H), 7.57–7.50 (m, 4H), 7.32–7.25 (m, 2H), 6.94 (s, 1H), 5.18 (d, *J* = 6.0 Hz, 2H). HRMS calcd. for C_25_H_17_FN_6_: 420.1499, found 420.1528.

### 4.6. MABA and LORA 

Minimum inhibitory concentration assays (MABA and LORA). These assays against *M.tb* (strain H37Rv) were performed according to the reported procedures [19,20]. The MIC was defined as the lowest compound concentration effecting a growth inhibition of at least 90% relative to the growth of drug-free controls. Results are the mean of two or three independent determinations, unless otherwise noted.

### 4.7. VERO Assay

In order to assess safety and selectivity in humans, all compounds were also screened for mammalian cell toxicity in VERO (green monkey kidney cell) cultures following the reported procedure [21].

### 4.8. Microsomal Stability

Assays were performed by WuXi AppTec (Shanghai) Co., Ltd. The test compounds (Table 4) (at 1 μM) were incubated at 37 °C with liver microsomes from human or CD-1 mice in the presence of a NADPH regenerating system and phosphate buffer (100 mM, pH 7.4) at 0.5 mg/mL microsomal protein (the positive controls were testosterone, propafenone, and diclofenac). Samples were removed at time intervals of 0, 5, 10, 20, 30, and 60 min and immediately mixed with cold CH_3_CN (containing 0.1 μg/mL of tolbutamide as an internal standard) and then centrifuged prior to analysis by LCMS/MS.

### 4.9. hERG Assay

Selected compounds were evaluated for hERG channel blockade (Table 4) by WuXi AppTec (Shanghai) Co., Ltd. This was carried out using cloned hERG potassium channels according to their standard manual patch clamp method. Two concentrations (0.3 and 1.0 µM) were tested (at room temperature), and at least three replicates were obtained for each. Amitryptyline was used as a standard.

## 5. Conclusions

We have shown here that the phenylpyrazolo[1,5-*a*]pyrimidin-7-amines are a readily accessible class of effective inhibitors of *M.tb* in culture. We have deduced broad structure–activity relationships that can serve as a guide for improvement of this class of compounds. The most effective analogues of pyrazolo[1,5-*a*]pyrimidin-7-amines were with the 3-(4-fluoro)phenyl group, the unsubstituted 5-phenyl group and a variety of substituted 7-(2-pyridylmethylamine) derivatives were tolerated. The most promising compounds were **52**, **53**, **54**, **55**, **62** and **63,** with excellent inhibitory data against *M.tb* and displaying low toxicity towards mammalian cells. These compounds exhibited good microsomal stability and very little inhibition of the hERG potassium ion channel. These findings show the potential of novel pyrazolo[1,5-*a*]pyrimidin-7-amines for further development into drug candidates for the treatment of tuberculosis.

## Figures and Tables

**Table 1:** Structures and biological data for 3-(phenyl-5-(substituted-phenyl)pyrazolo[1,5-*a*]pyrimidine-7-amines (compounds **11–21**).

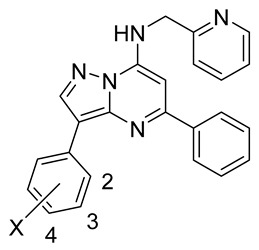					
No	X	MIC_90_^a^ (µg/mL)		IC_50_^b^ (µg/mL)	clogP ^c^
		MABA	LORA	VERO	
**11**	2-F	>32	>32	>32	5.07
**12**	2-OMe	>32	>32	>32	4.29
**13**	2-CF_3_	>32	>32	>32	5.81
**14**	3-F	>32	>32	>32	5.07
**15**	3-Me	>32	30	>32	5.07
**16**	3-OMe	7.6	10	>32	4.29
**17**	3-CF_3_	>32	>32	>32	5.81
**18**	4-F	3.8	0.40	>32	5.07
**19**	4-Me	>32	>32	>32	5.13
**20**	4-OMe	3.6	0.51	>32	4.29
**21**	4-CF_3_	>32	>32	>32	5.81

Table 1 ^a:^MIC_90_ (µg/mL); minimum inhibitory concentration for 90% inhibition of growth of M.tb strain H37Rv, determined under aerobic/replicating (MABA) [19] or non-replicating (LORA) [20] conditions, determined at the Institute for Tuberculosis Research, University of Illinois at Chicago; ^b^ IC50 values (µg/mL) for the inhibition of growth of VERO green monkey kidney epithelial cells [21]. ^c^ clogP values calculated by ChemDraw Ultra v21.0.0.28 (CambridgeSoft).

**Table 2:** Structures and biological data for 5-substituted 3-(4-fluorophenyl)*-N-*(pyridin-2-ylmethyl)pyrazolo[1,5-*a*]pyrimidin-7-amines (compounds **22–44**).

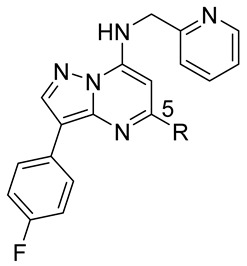					
No	R	MIC_90_^a^ (µg/mL)		IC_50_^b^ (µg/mL)	
		MABA	LORA	VERO	clogP ^c^
**22**	H	0.89	0.39	>32	2.97
**23**	Et	1.40	0.45	14.1	4.00
**24**	CH=CH_2_	1.48	0.47	17.2	3.70
**25**	c-propyl	1.54	0.20	23.4	3.94
**26**	c-pentenyl	0.89	0.23	>32	4.73
**27**	c-hexenyl	0.62	0.44	>32	5.29
**28**	2-FPh	1.79	0.22	>32	5.21
**29**	2-OMePh	13.3	9.0	>32	4.52
**30**	3-MePh	1.6	0.9	>32	5.57
**31**	3-OMePh	3.9	1.7	>32	5.08
**32**	4-FPh	3.0	0.22	>32	5.21
**33**	4-MePh	3.7	5.0	>32	5.57
**34**	4-OMePh	1.5	0.25	>32	5.08
**35**	4-^i^PrPh	1.8	1.0	>32	6.50
**36**	4-^t^BuPh	1.7	1.9	>32	6.90
**37**	4-CF_3_Ph	3.8	3.6	>32	5.95
**38**	4-OCF_3_Ph	1.9	1.1	>32	6.20
**39**	3-pyridyl	2.3	0.30	>32	3.64
**40**	4-pyridyl	>32	28	>32	3.64
**41**	2-thienyl	0.99	0.36	>32	4.96
**42**	3-thienyl	1.4	0.22	>32	4.74
**43**	2-furyl	0.96	<0.13	>32	4.46
**44**	3-furyl	0.96	0.16	>32	4.35

Footnotes for Table 2 As for Table 1.

**Table 3:** Structures and biological data for 7-(2-pyridylmethylamine) ring variations (compounds **45–72**).

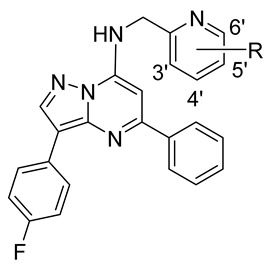					
No	R	MIC_90_ (µg/mL) ^a^		IC_50_^b^ (µg/mL)	clogP ^b^
		MABA	LORA	VERO	
**45**	6′-F	>32	>32	>32	5.30
**46**	6′-Br	>32	>32	>32	6.02
**47**	6′-Me	>32	>32	>32	5.57
**48**	6′-OMe	>32	>32	>32	5.89
**49**	6′-NMe_2_	1.39	0.40	>32	5.78
**50**	6′-Npiperidine	0.25	1.75	>32	6.46
**51**	6′-NMepiperazine	0.94	0.85	11.8	5.64
**52**	5′-F	3.7	3.4	>32	6.30
**53**	5′-Cl	4.9	7.2	>32	5.87
**54**	5′-OMe	3.6	1.3	>32	5.49
**55**	5′-C≡CCH_2_NMe_2_	1.9	5.5	>32	4.65
**56**	5′-(CH_2_)_3_NMe_2_	5.8	6.1	20.3	5.42
**57**	5′-O(CH_2_)_2_NMe_2_	3.6	1.9	24.0	5.59
**58**	5′-O(CH_2_)_3_NMe_2_	4.7	4.1	>32	5.94
**59**	5′-Npiperidine	0.96	0.85	>32	6.46
**60**	4′-Cl	>32	>32	>32	5.87
**61**	4′-CN	>32	>32	>32	4.70
**62**	4′-Me	1.8	0.5	>32	5.57
**63**	4′-OMe	1.9	0.5	>32	5.49
**64**	4′-Npyrrolidine	0.47	0.94	16.0	5.90
**65**	4′-NMepiperazine	0.09	0.73	10.7	5.64
**66**	4′-Nmorpholine	0.06	0.98	>32	5.08
**67**	4′-N(Me)(CH_2_)_2_NMe_2_	1.7	1.5	9.9	6.02
**68**	4′-Npiperidine	0.96	0.85	>32	6.45
**69**	3′-NMe_2_	1.5	0.49	>32	5.78
**70**	3′-F	>32	>32	>32	5.30
**71**	3′-Br	>32	>32	>32	6.02
**72**	3′-CN	>32	>32	>32	4.84

^a:^MIC_90_ (µg/mL); minimum inhibitory concentration for 90% inhibition of growth of M.tb strain H37Rv, determined under aerobic/replicating (MABA) [19] or non-replicating (LORA) [20] conditions, determined at the Institute for Tuberculosis Research, University of Illinois at Chicago; ^b^ IC50 values (µg/mL) for the inhibition of growth of VERO green monkey kidney epithelial cells [21].

**Table 4:** Microsomal stability and hERG inhibitory properties for the selected compounds.

No	HLM ^a^	MLM ^b^	HLM ^a^	HLM ^a^	MLM ^b^	MLM ^b^	hERG ^c^
	T½ (min)	T½ (min)	Cl*_int_*(mic) (µL/min/mg)	CL*_int_*(liver) (mL/min/kg)	Cl*_int_*(mic) (µL/min/mg)	CL*_int_*(liver) (mL/min/kg)	% inhib. at 0.3/1.0 µM
**18**	105	56	13	12	25	99	0.3/5
**20**	62	60	22	20	23	92	0/0
**25**	18	13	76	68	107	424	8/19
**26**	39	39	35	32	35	140	ND
**27**	75	49	19	17	30	120	ND
**28**	69	49	20	18	29	113	17/36
**30**	>145	>145	<9.6	<8.6	<9.6	<38	3/11
**34**	77	63	18	16	22	88	0/1
**35**	>145	>145	<9.6	<8.6	<9.6	<38	1/1
**36**	>145	>145	<9.6	<8.6	<9.6	<38	0/0
**38**	86	>145	16	15	<9.6	<38	2/2
**39**	22	12	63	57	112	445	16/46
**41**	17	34	83	75	41	163	0/0
**42**	29	18	47	43	75	298	9/24
**44**	19	10	74	66	144	570	4/14
**52**	108.5	109	13	11	12.7	50.3	0.8/11
**53**	>145	>145	<9.6	<8.6	<9.6	<38	0.7/0
**54**	>145	>145	<9.6	<8.6	<9.6	<38	ND
**55**	>145	45.4	<9.6	<8.6	31	121	ND
**62**	>145	>145	<9.6	<8.6	<9.6	<38	ND
**63**	>145	>145	<9.6	<8.6	<9.6	<38	2.3/4.6
**68**	112	60	12	11	23	92	19/36

Footnotes for Table 4: ^a^ HLM: human liver microsomes. ^b^ MLM: mouse liver microsomes. ^c^ hERG: %inhibition of the human rapidly activating delayed rectifier potassium channel protein encoded by the ether-a-go-go-related gene (hERG) at 0.3 and 1.0 µM drug concentrations. ND: not done.

## Data Availability

Samples of compounds in Table 4 are available from the authors.

## Supplementary Materials

The following supporting information can be downloaded at: https://www.mdpi.com/article/10.3390/ph15091125/s1. ^1^H and ^13^C Spectra for compounds that progressed to advanced testing.
