# Supplementary material for: Synthesis and Structure–Activity Relationships for the Anti-Mycobacterial Activity of 3-Phenyl-N-(Pyridin-2-ylmethyl)Pyrazolo[1,5-a]Pyrimidin-7-Amines
Source: Pharmaceuticals (Basel). 2022 Sep 8;15(9):1125. doi: 10.3390/ph15091125 (PMC9506485; doi:10.3390/ph15091125)
Supplement: Supplementary file 1 [file pharmaceuticals-15-01125-s001.zip › pharmaceuticals-1875515-supplementary.pdf]

Supplementary Material

Synthesis and structure-activity relationships for the anti-mycobacterial activity of 3-phenyl-*N*-(pyridin-2-ylmethyl)pyrazolo[1,5-*a*]pyrimidin-7-amines

Hamish S. Sutherland [a, b], Peter J. Choi [a, b*], Guo-Liang Lu [a, b], Anna C. Giddens [a], Amy S.T. Tong [a], Scott G. Franzblau [c], Christopher B. Cooper [d], Brian D. Palmer [a, b], William A. Denny [a, b]

[a] Auckland Cancer Society Research Centre, School of Medical Sciences, University of Auckland, Private Bag 92019, Auckland, 1142, New Zealand
[b] Maurice Wilkins Centre, University of Auckland, Auckland, 1142, New Zealand
[c] Institute for Tuberculosis Research, College of Pharmacy, University of Illinois at Chicago, 833 South Wood Street, Chicago, IL, 60612, USA
[d] Global Alliance for TB Drug Development, 40 Wall St, New York, 10005, USA

* Corresponding author. Auckland Cancer Society Research Centre, School of Medical Sciences, University of Auckland, New Zealand.
E-mail address: p.choi@auckland.ac.nz

CONTENTS

General Procedure and experimental 2

^{1}H Spectra for compounds that progressed to advanced testing 3-24

General information

Final products were analysed by reverse-phase HPLC (Alltima C18 5 µm column, 15 × 3.2 mm; Alltech Associated, Inc., Deerfield, IL) using an Agilent HP1100 equipped with a diode-array detector. It was run using mobile phases with 80% CH_3CN/20% H_2O (v/v) in 45 mM NH_4HCO_2 at pH 3.5 and 0.5 mL/min. Purity level was determined by monitoring at 330 ± 50 nm and was ≥95% for all final products. NMR spectra were obtained on a Bruker Avance 400 spectrometer at 400 MHz for ^{1}H and ^{13}C. Low-resolution mass spectra (LRMS), using atmospheric pressure chemical ionization (APCI) were measured on a ThermoFinnigan Surveyor MSQ mass spectrometer, connected to a Gilson autosampler. High resolution mass spectra (HRMS) were obtained using an Agilent G6530B Q-TOF spectrometer and are reported as M+H. Melting points were determined on an Electrothermal 9100 melting point apparatus.

^{1}H NMR and ^{13}C NMR Spectra for compounds that progressed to advanced testing

Compound **18**

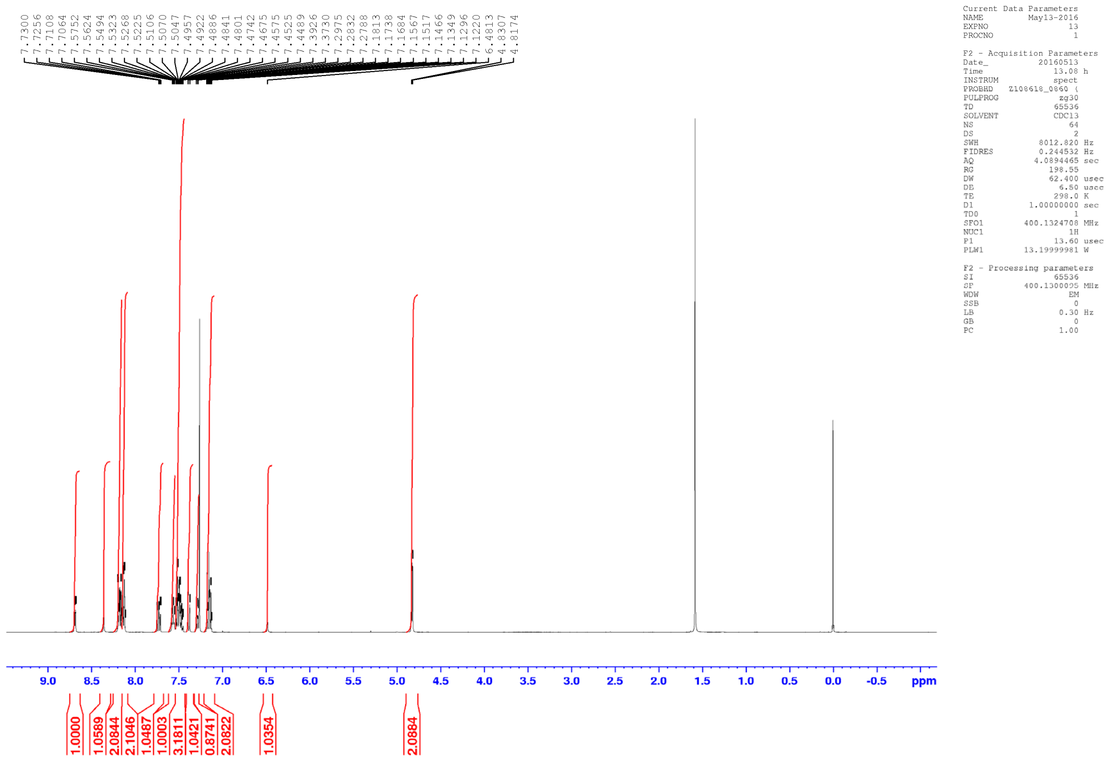

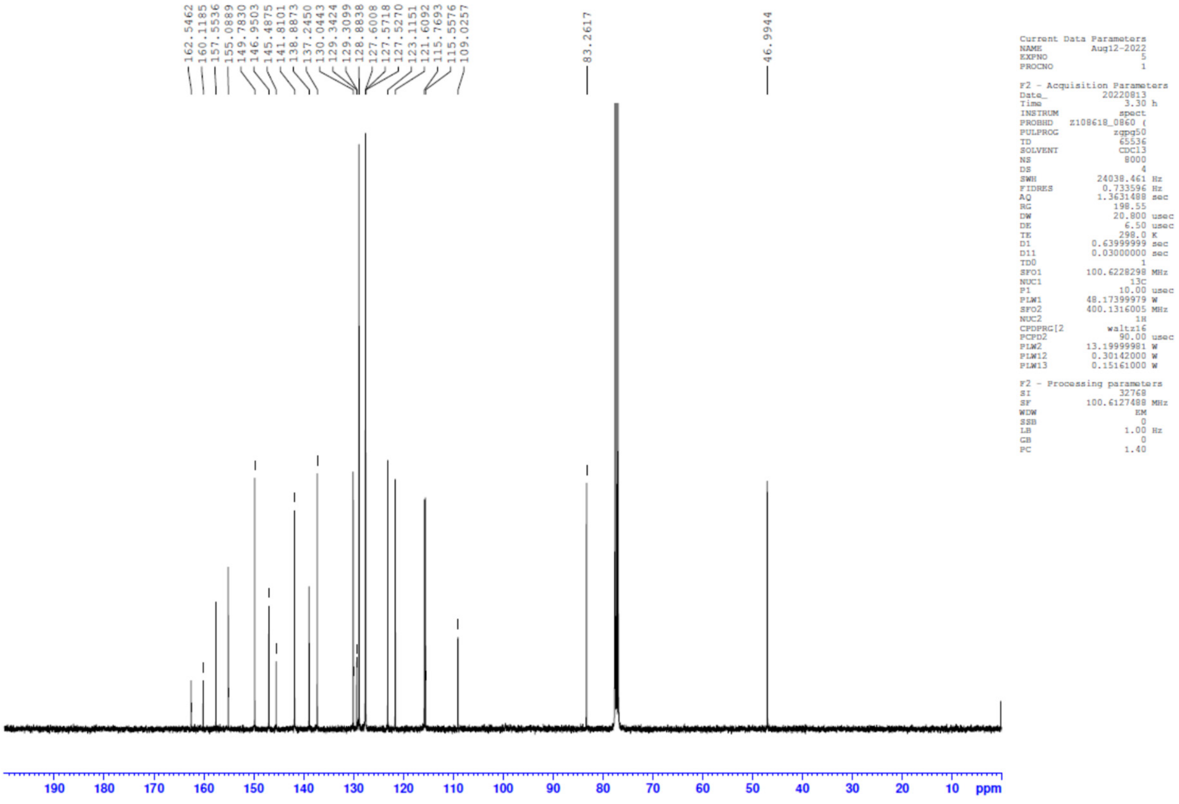

Compound **20**

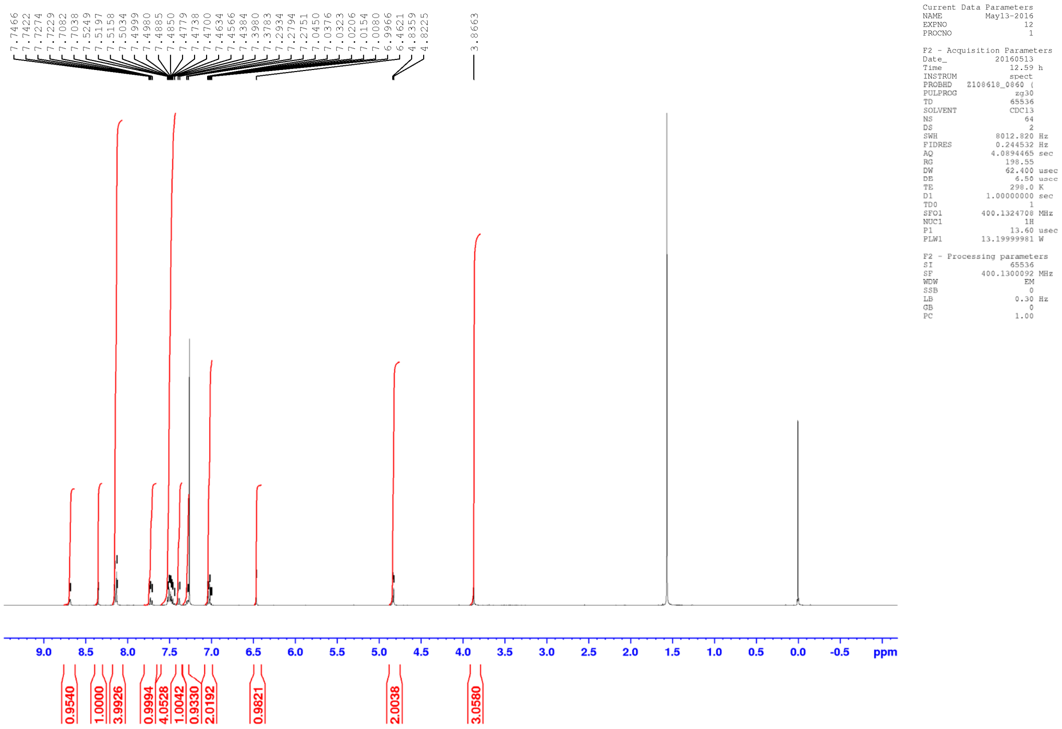

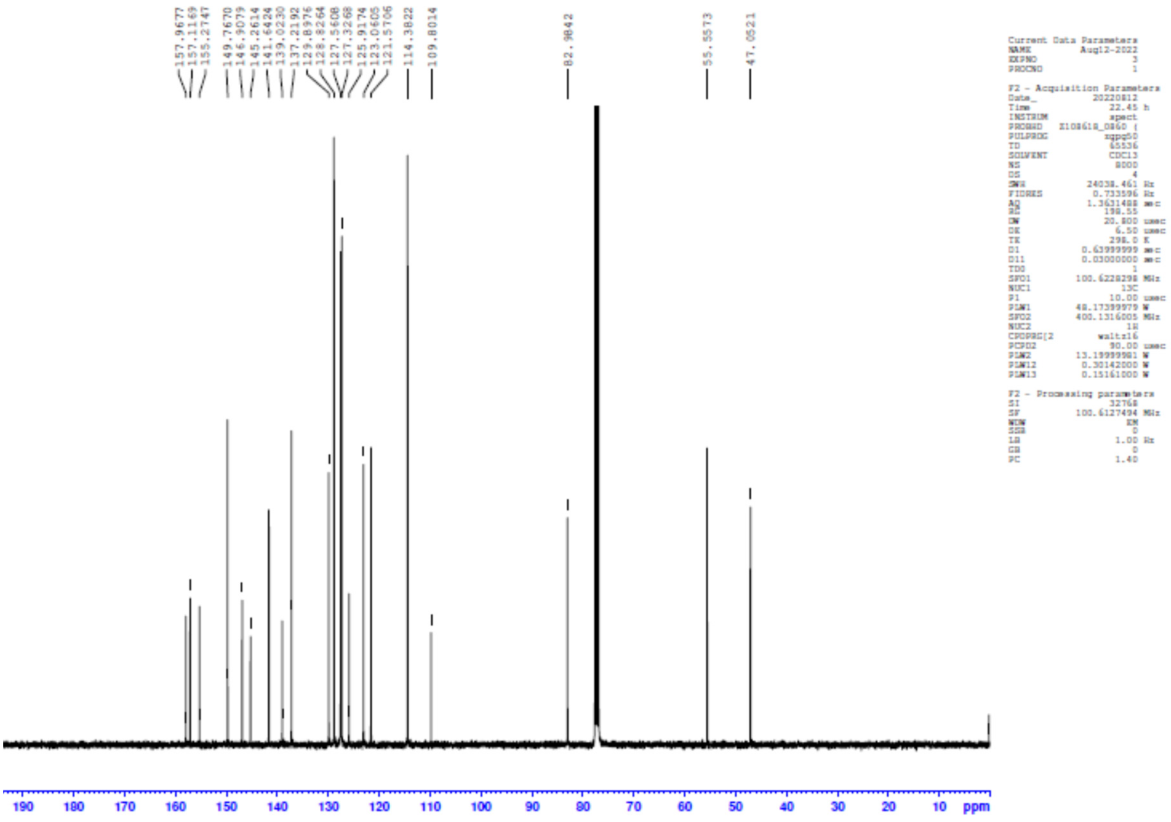

Compound **25**

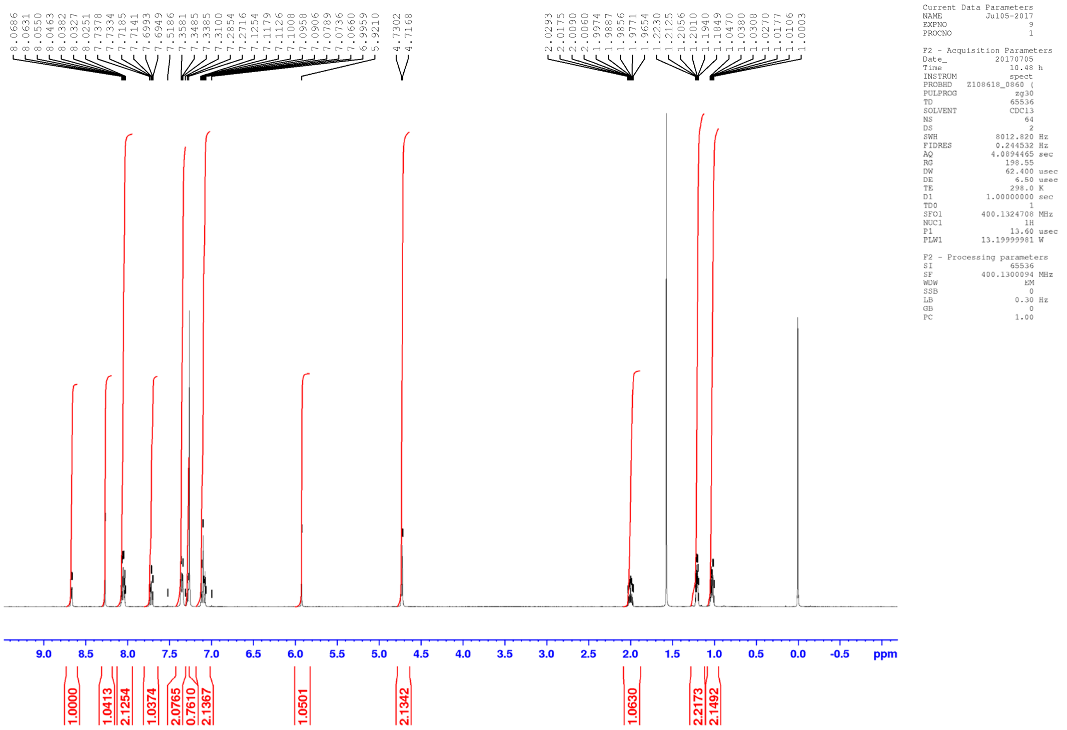
8.0686
8.0631
8.0550
8.0463
8.0382
8.0327
8.0251
7.7378
7.7334
7.7185
7.7141
7.6993
7.6949
7.5186
7.3581
7.3485
7.3385
7.3100
7.2854
7.2716
7.1254
7.1179
7.1126
7.1008
7.0958
7.0906
7.0789
7.0736
7.0660
6.9959
5.9210
4.7302
4.7168
2.0293
2.0175
2.0090
2.0060
1.9974
1.9887
1.9856
1.9771
1.9654
1.2230
1.2125
1.2056
1.2010
1.1940
1.1849
1.0470
1.0380
1.0308
1.0270
1.0177
1.0106
1.0003
9.0 8.5 8.0 7.5 7.0 6.5 6.0 5.5 5.0 4.5 4.0 3.5 3.0 2.5 2.0 1.5 1.0 0.5 0.0 -0.5 ppm
1.0000
1.0413
2.1254
1.0374
2.0765
0.7610
2.1367
1.0501
2.1342
1.0630
2.2173
2.1492
Current Data Parameters
NAME Ju105-2017
EXPNO 9
PROCNO 1
F2 - Acquisition Parameters
Date_ 20170705
Time 10.48 h
INSTRUM spect
PROBHD Z108618_0860 (
PULPROG zg30
TD 65536
SOLVENT CDCl3
NS 64
DS 2
SWH 8012.820 Hz
FIDRES 0.244532 Hz
AQ 4.0894465 sec
RG 198.55
DW 62.400 usec
DE 6.50 usec
TE 298.0 K
D1 1.00000000 sec
TD0 1
SFO1 400.1324708 MHz
NUC1 1H
P1 13.60 usec
PLW1 13.19999981 W
F2 - Processing parameters
SI 65536
SF 400.1300094 MHz
WDW EM
SSB 0
LB 0.30 Hz
GB 0
PC 1.00

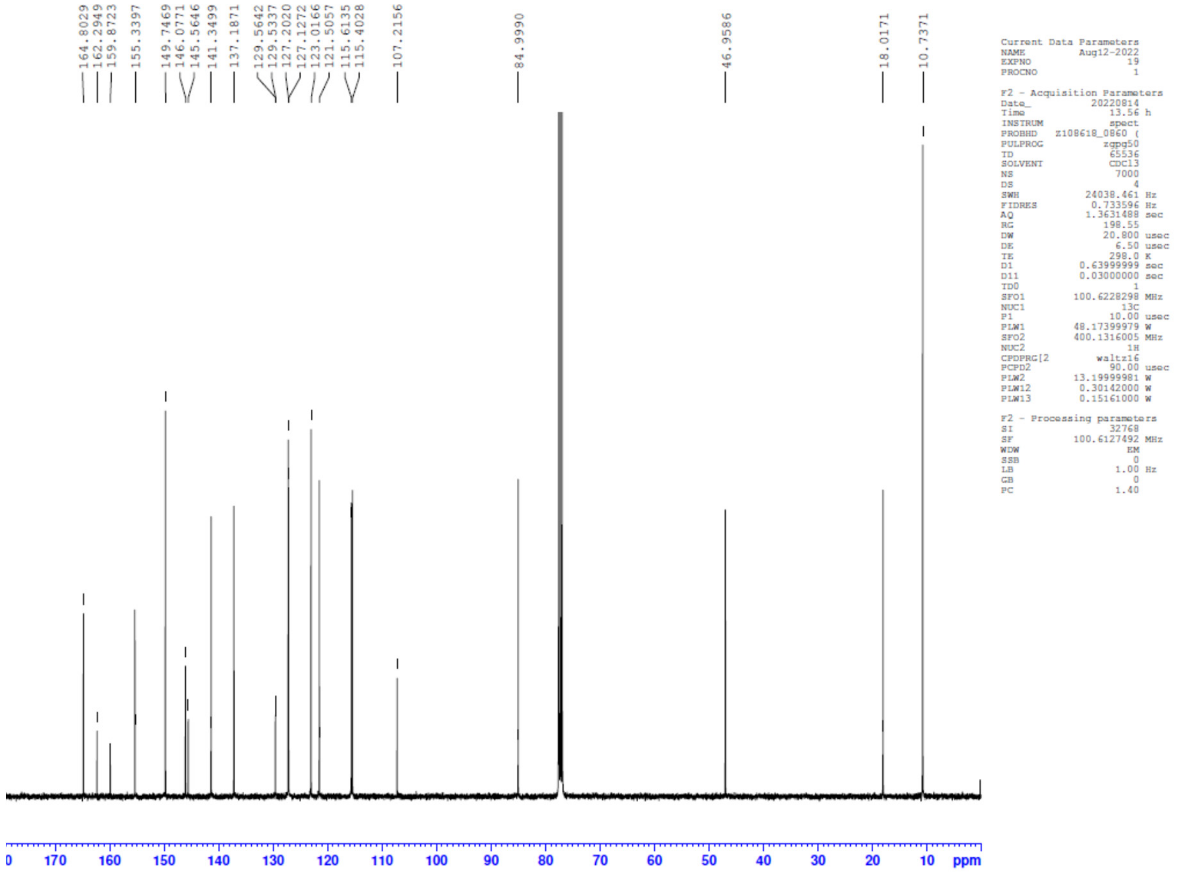
164.8029
162.2949
159.8723
155.3397
149.7469
146.0771
145.5646
141.3499
137.1871
129.5642
129.5337
127.2020
127.1272
123.0166
121.5057
115.6135
115.4028
107.2156
84.9990
46.9586
18.0171
10.7371
170 160 150 140 130 120 110 100 90 80 70 60 50 40 30 20 10 ppm
Current Data Parameters
NAME Aug12-2022
EXPNO 19
PROCNO 1
F2 - Acquisition Parameters
Date_ 20220814
Time 13.56 h
INSTRUM spect
PROBHD Z108618_0860 (
PULPROG zgpg30
TD 65536
SOLVENT CDCl3
NS 7000
DS 4
SWH 24038.461 Hz
FIDRES 0.733596 Hz
AQ 1.3631488 sec
RG 198.55
DW 20.800 usec
DE 6.50 usec
TE 298.0 K
D1 0.63999999 sec
D11 0.03000000 sec
TD0 1
SFO1 100.6228298 MHz
NUC1 13C
P1 10.00 usec
PLW1 48.17399979 W
SFO2 400.1316005 MHz
NUC2 1H
CPDPRG[2 waltz16
PCPD2 90.00 usec
PLW2 13.19999981 W
PLW12 0.30142000 W
PLW13 0.15161000 W
F2 - Processing parameters
SI 32768
SF 100.6127492 MHz
WDW EM
SSB 0
LB 1.00 Hz
GB 0
PC 1.40

Compound **26**

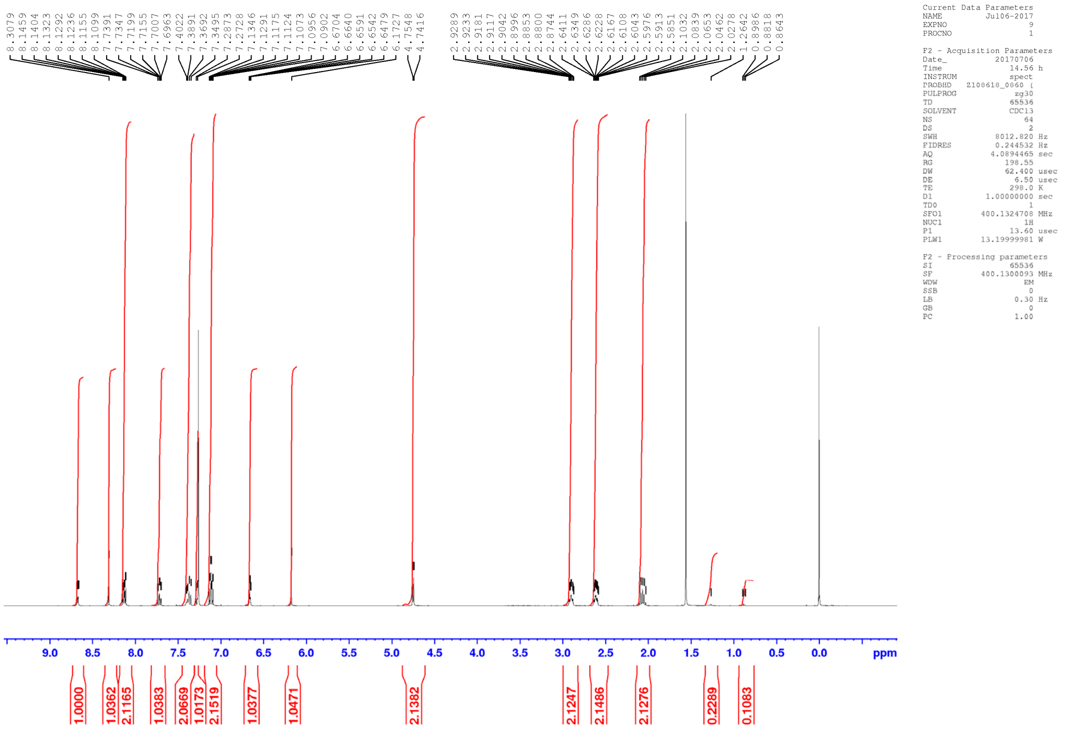

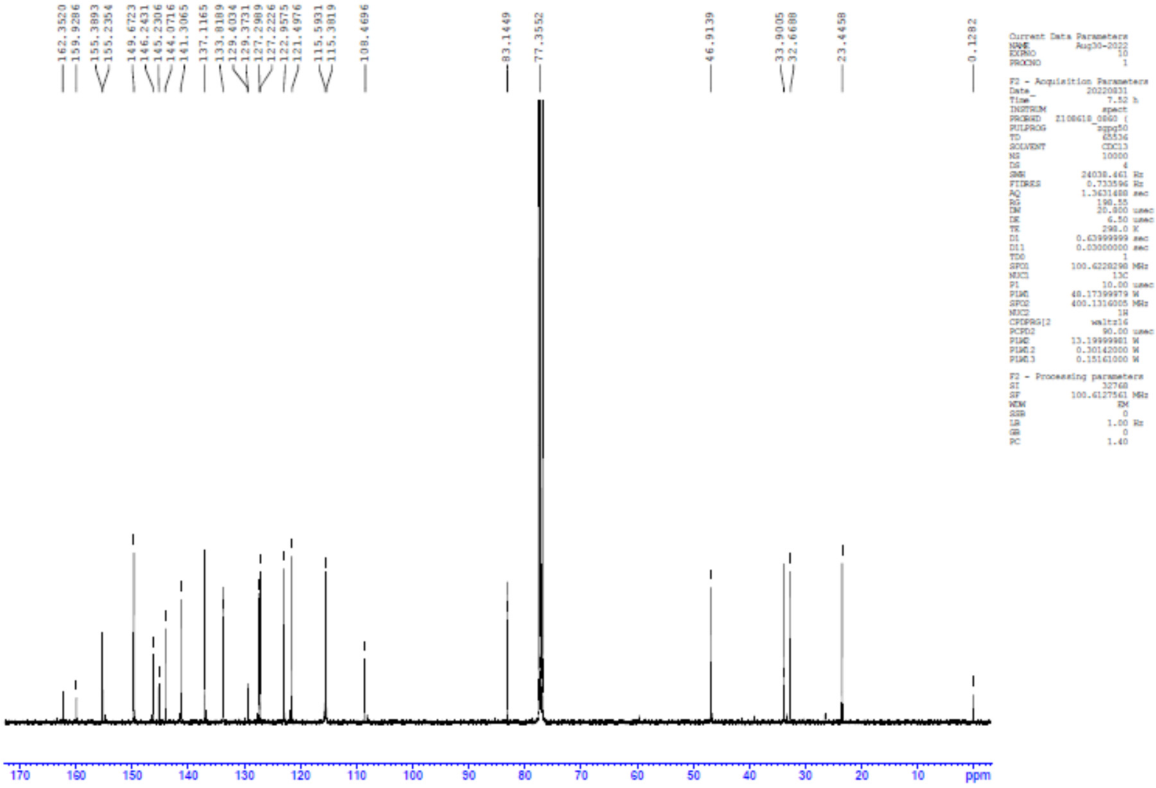

Compound **27**

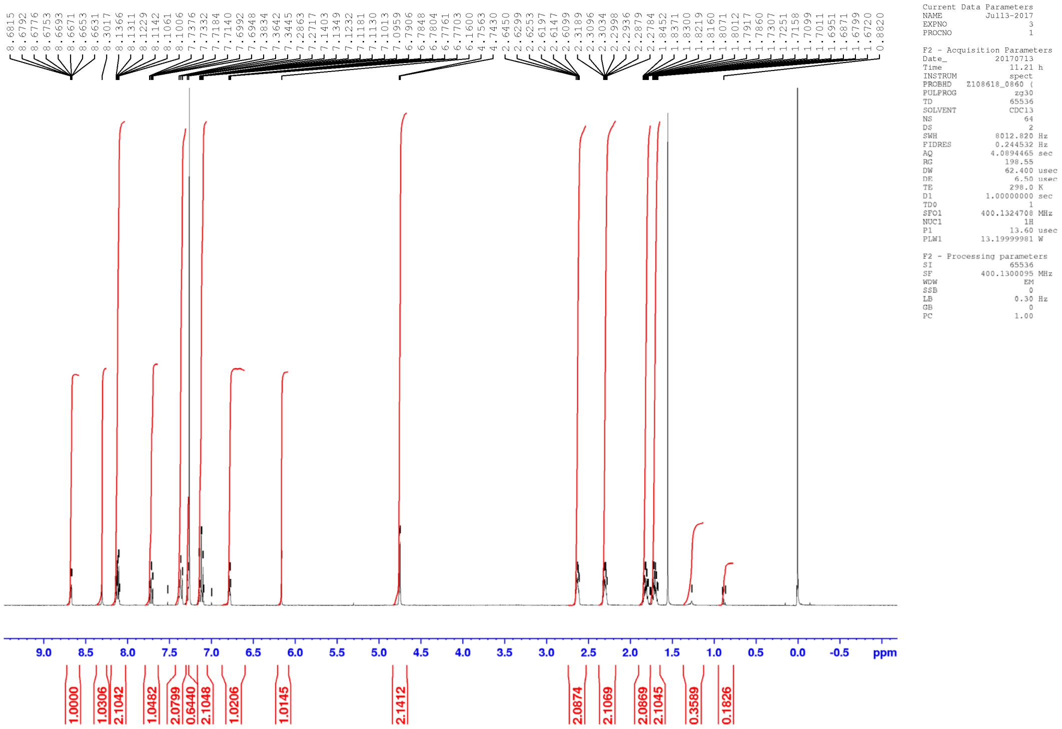

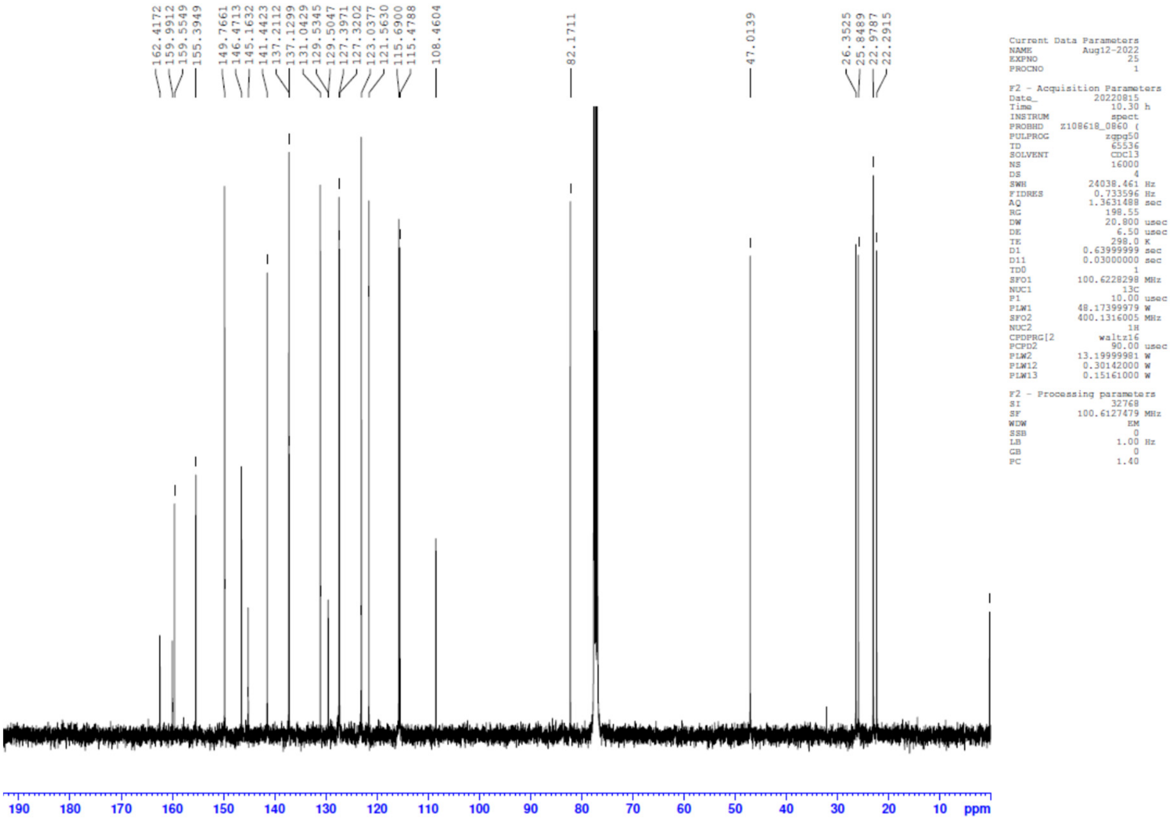

Compound **28**

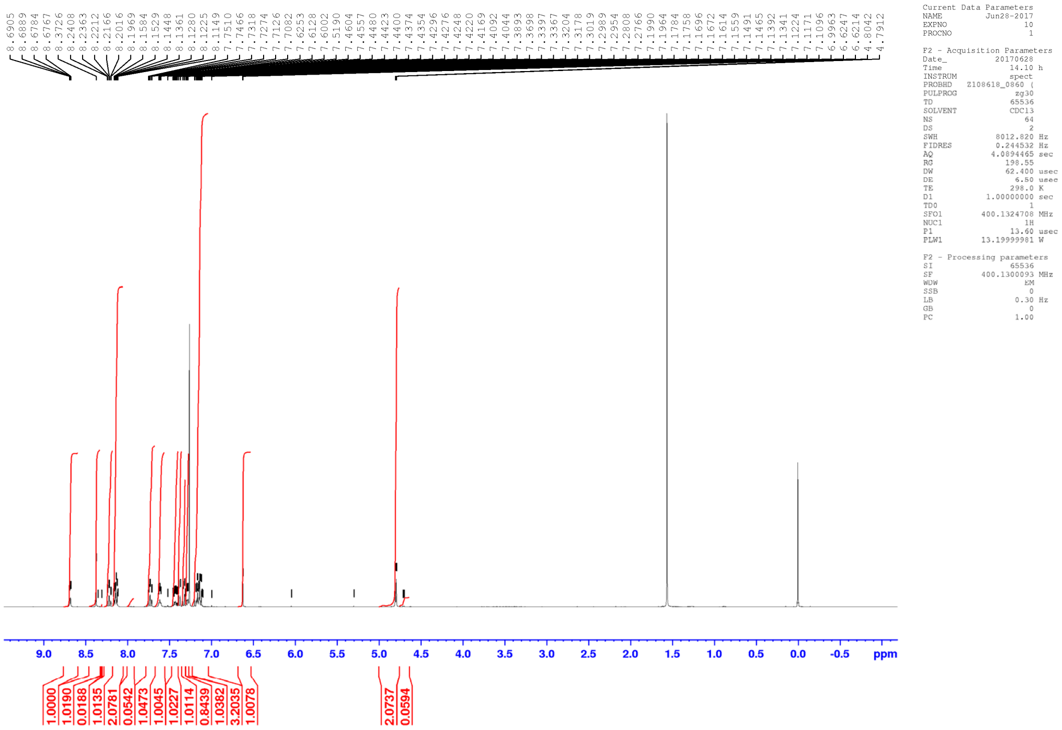

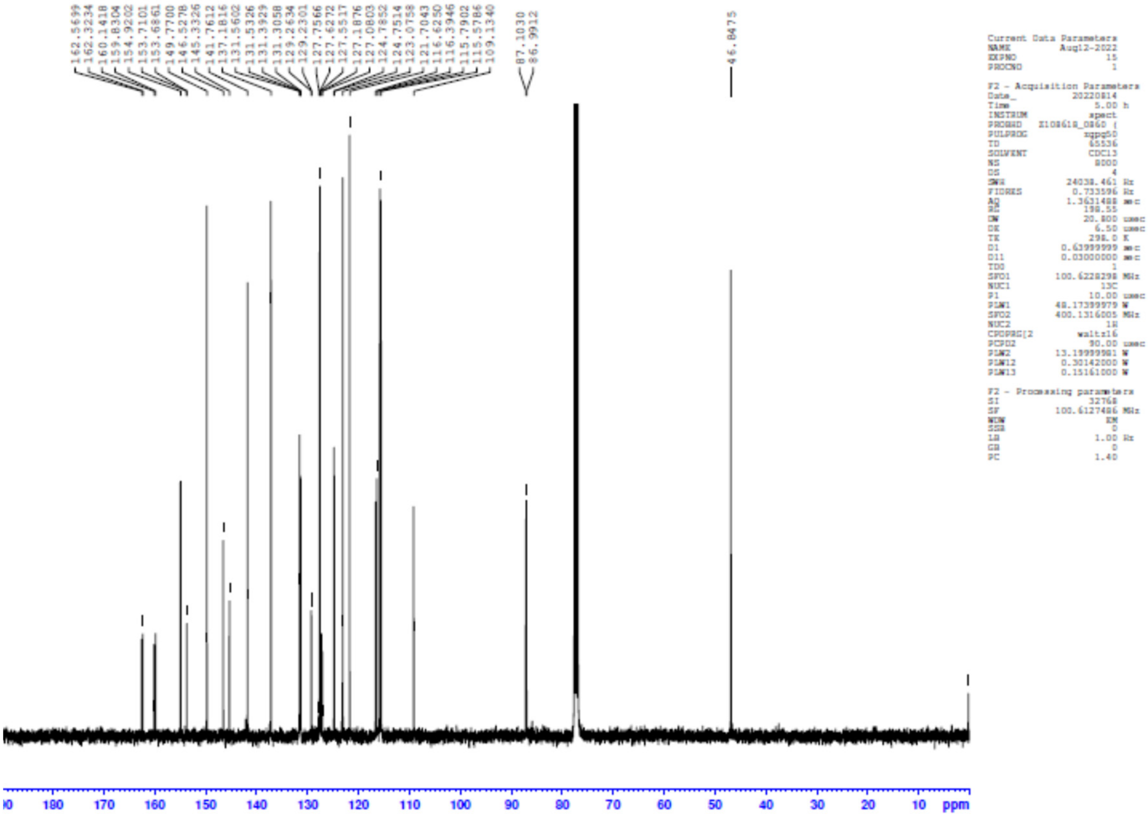

Compound **30**

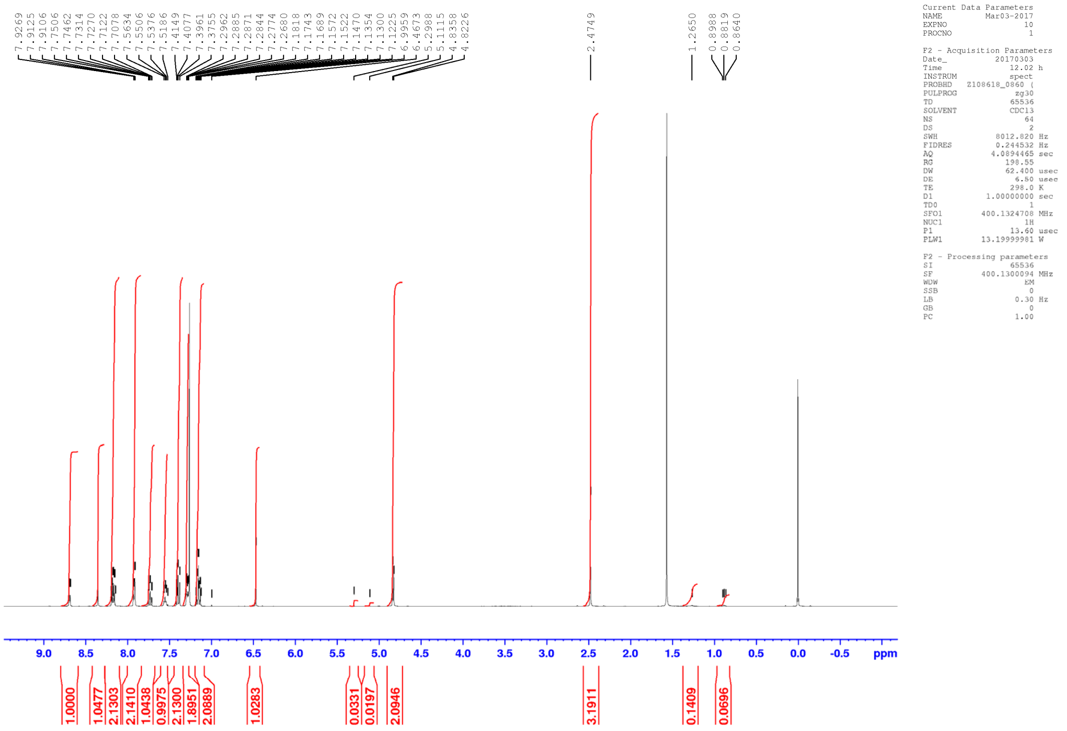

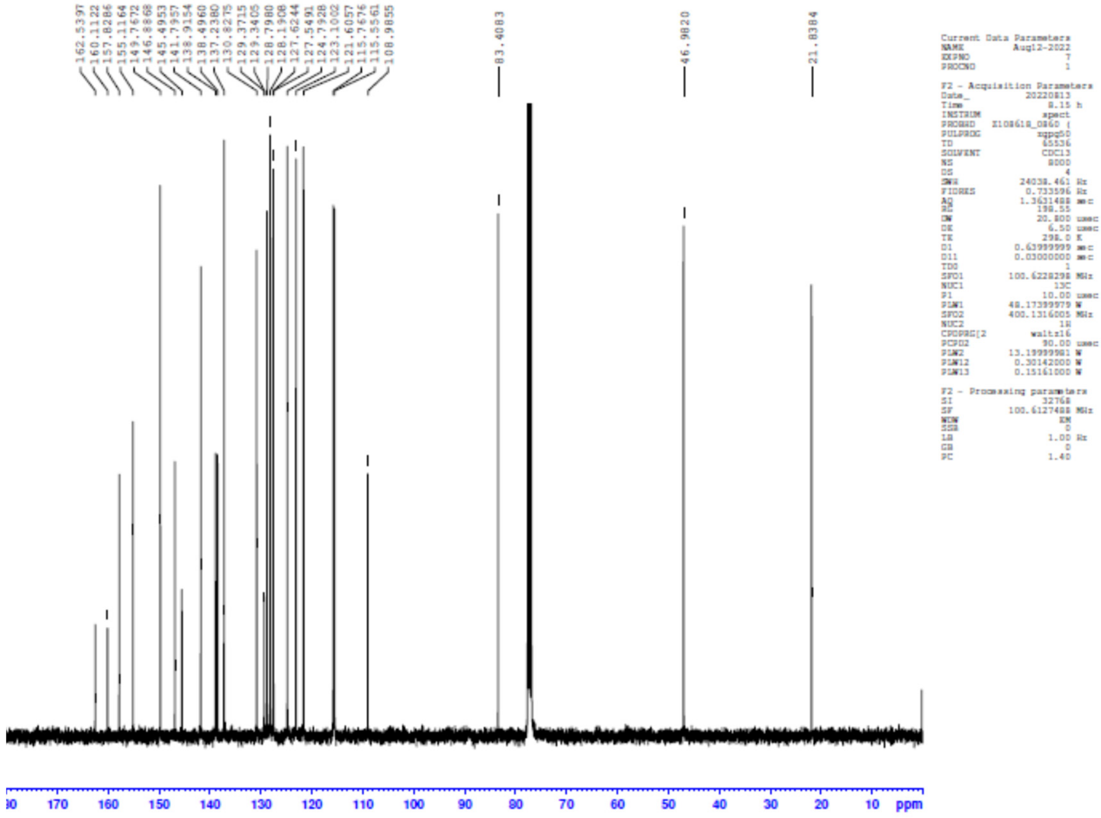

Compound **34**

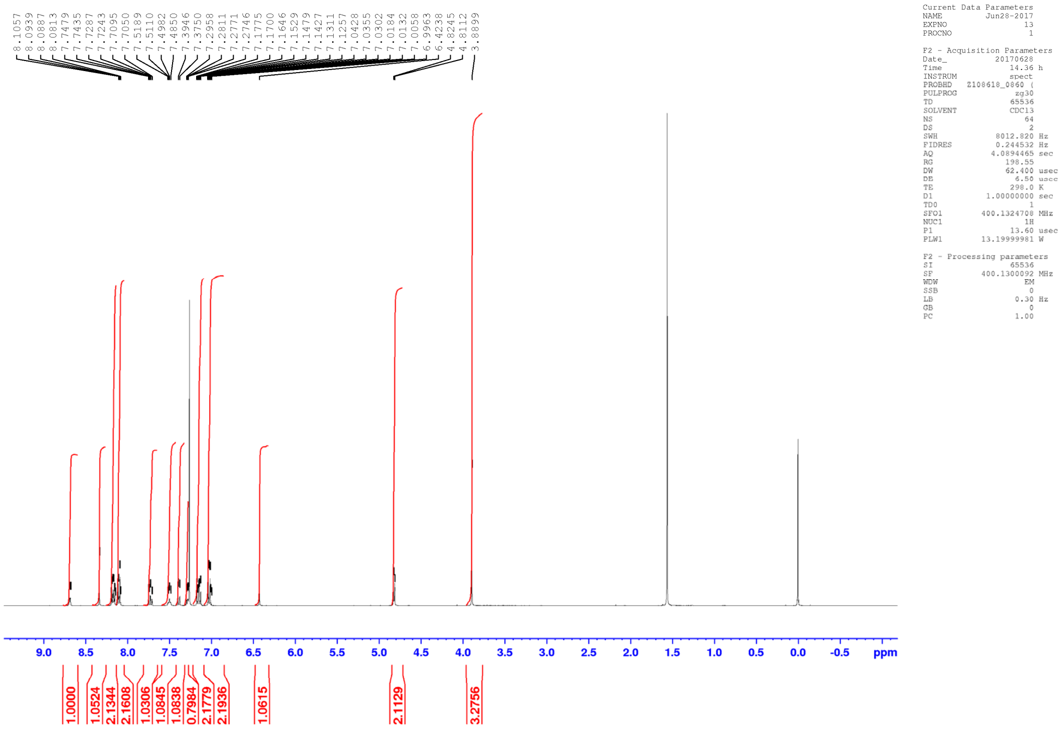

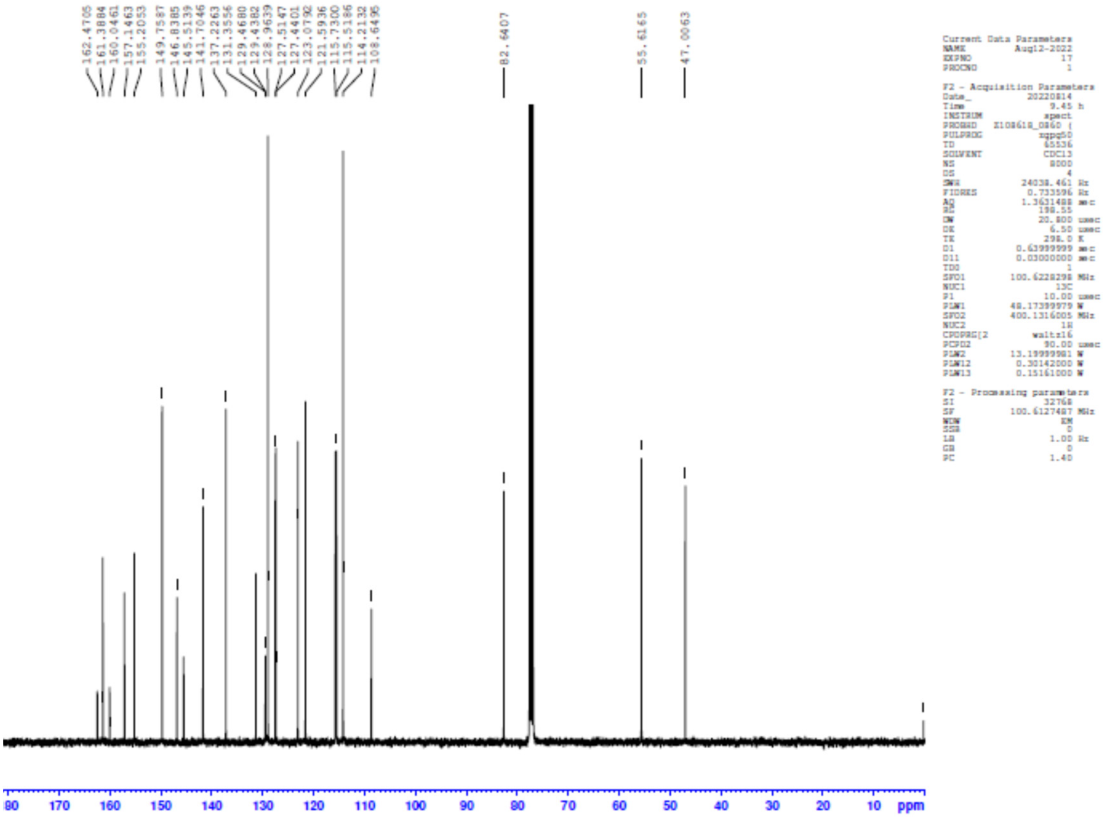

Compound **35**

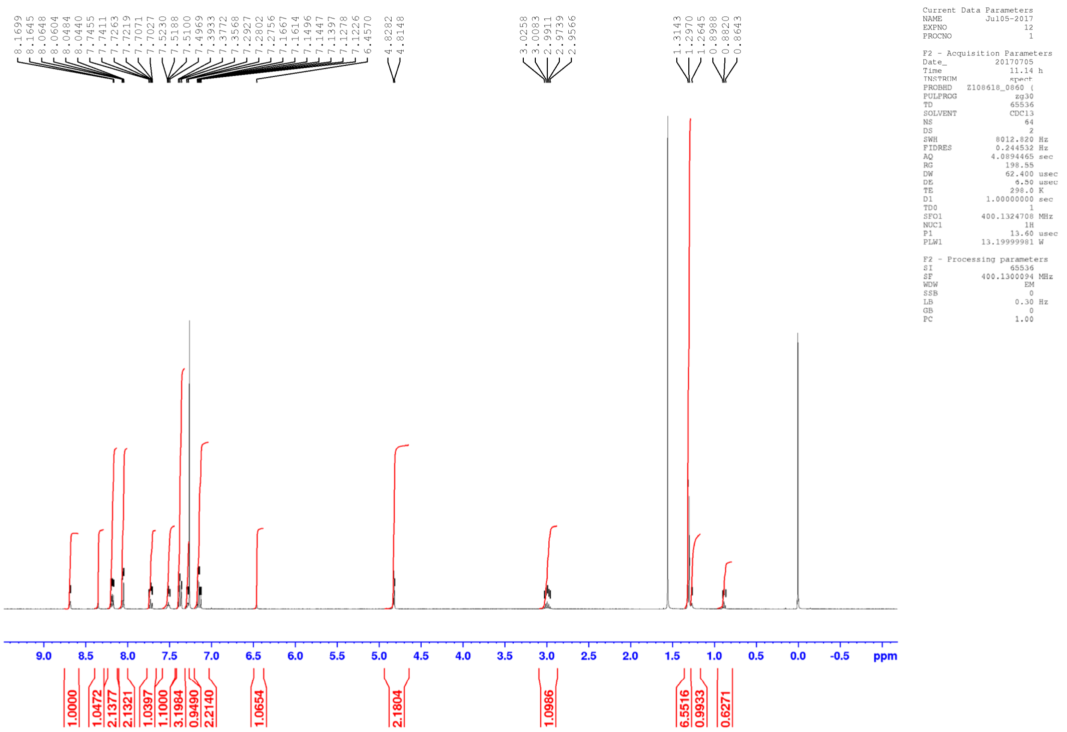

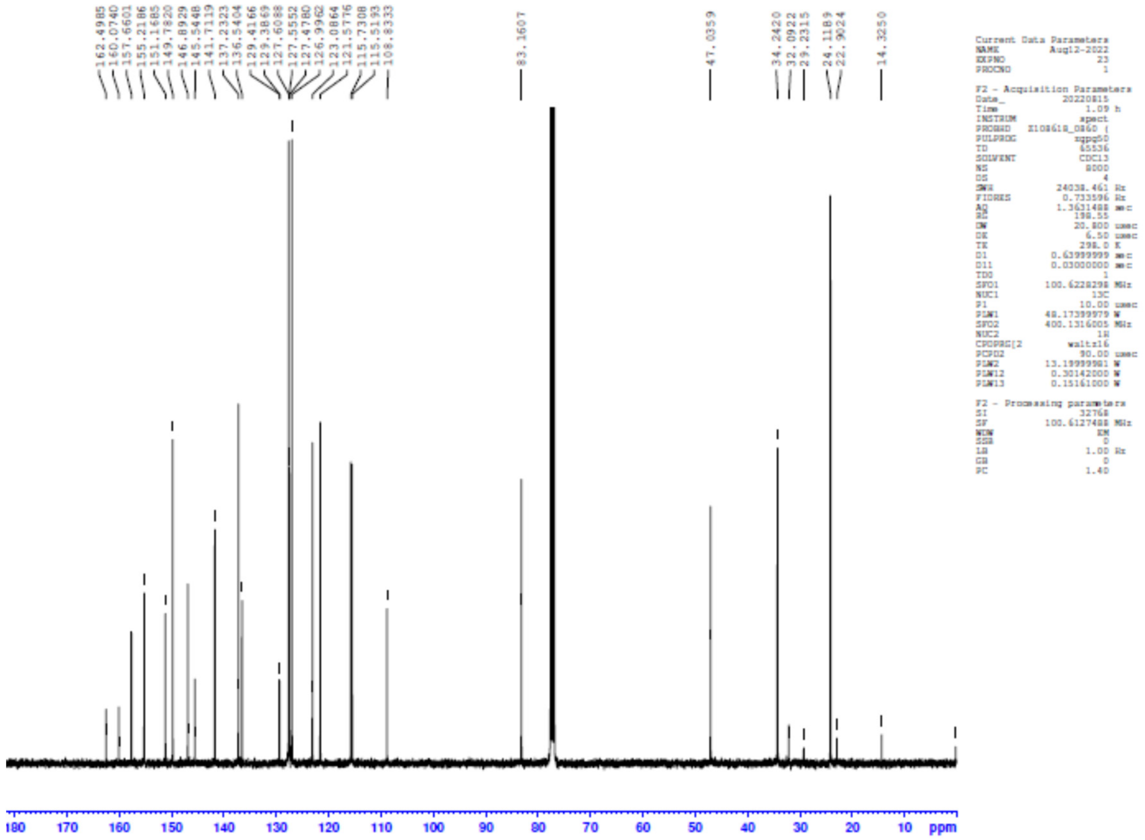

Compound **36**

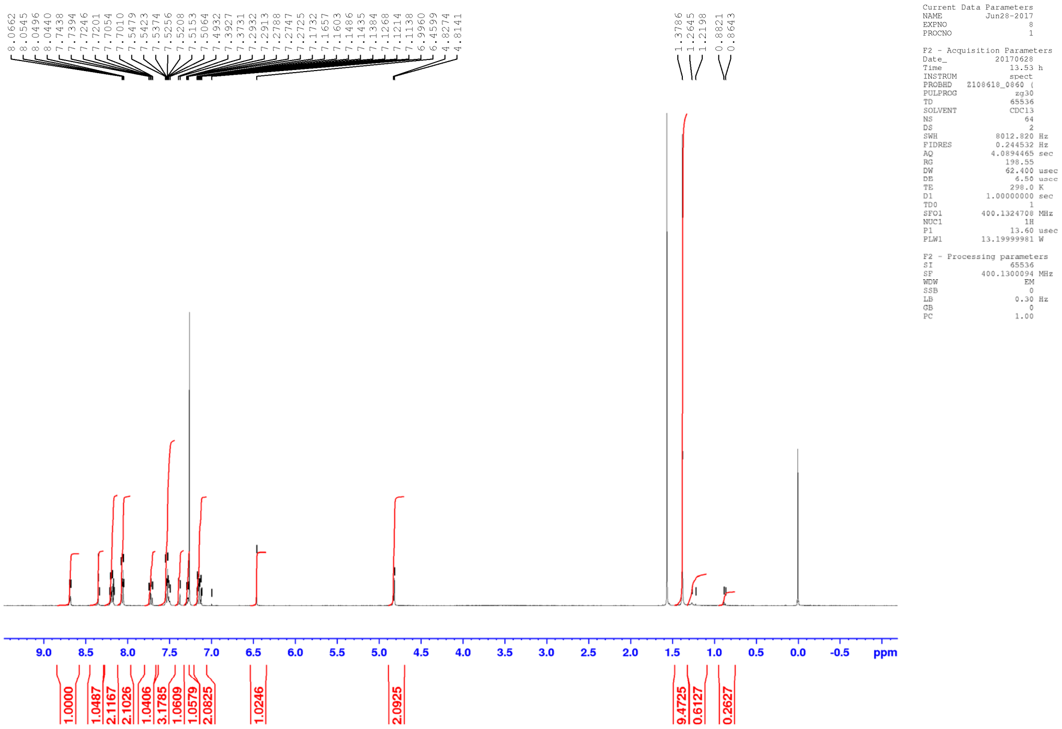
8.0662
8.0545
8.0496
8.0440
7.7438
7.7394
7.7246
7.7201
7.7054
7.7010
7.5479
7.5423
7.5374
7.5256
7.5208
7.5153
7.5064
7.4932
7.3927
7.3731
7.2932
7.2913
7.2788
7.2747
7.2725
7.1732
7.1657
7.1603
7.1486
7.1435
7.1384
7.1268
7.1214
7.1138
6.9960
6.4599
4.8274
4.8141
1.3786
1.2645
1.2198
0.8821
0.8643
ppm
1.0000
1.0487
2.1167
2.1026
1.0406
3.1785
1.0609
1.0579
2.0825
1.0246
2.0925
9.4725
0.6127
0.2627

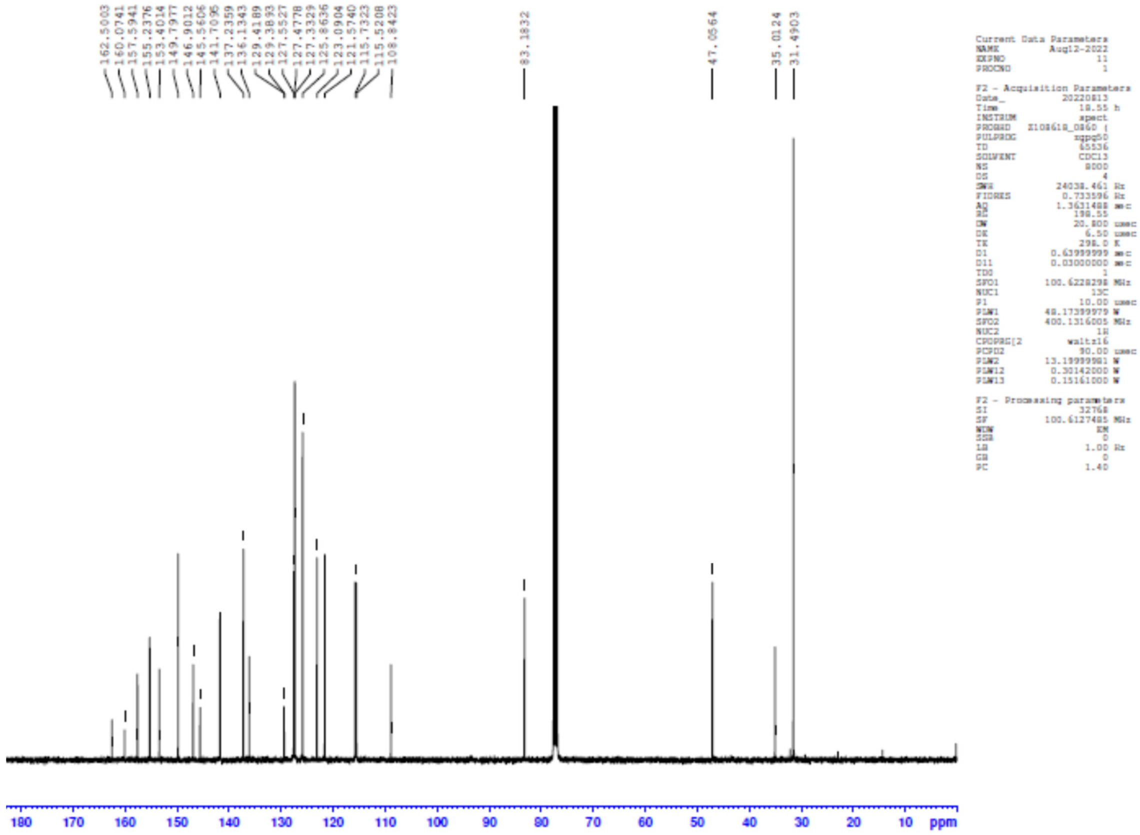
162.5003
160.0741
157.5941
155.2376
153.4014
149.7977
146.9012
145.5606
141.7095
137.2359
136.1343
129.4189
129.3893
127.5527
127.4778
127.3329
125.8636
123.0904
121.5740
115.7323
115.5208
108.8423
83.1832
47.0564
35.0124
31.4903
ppm

Compound **38**

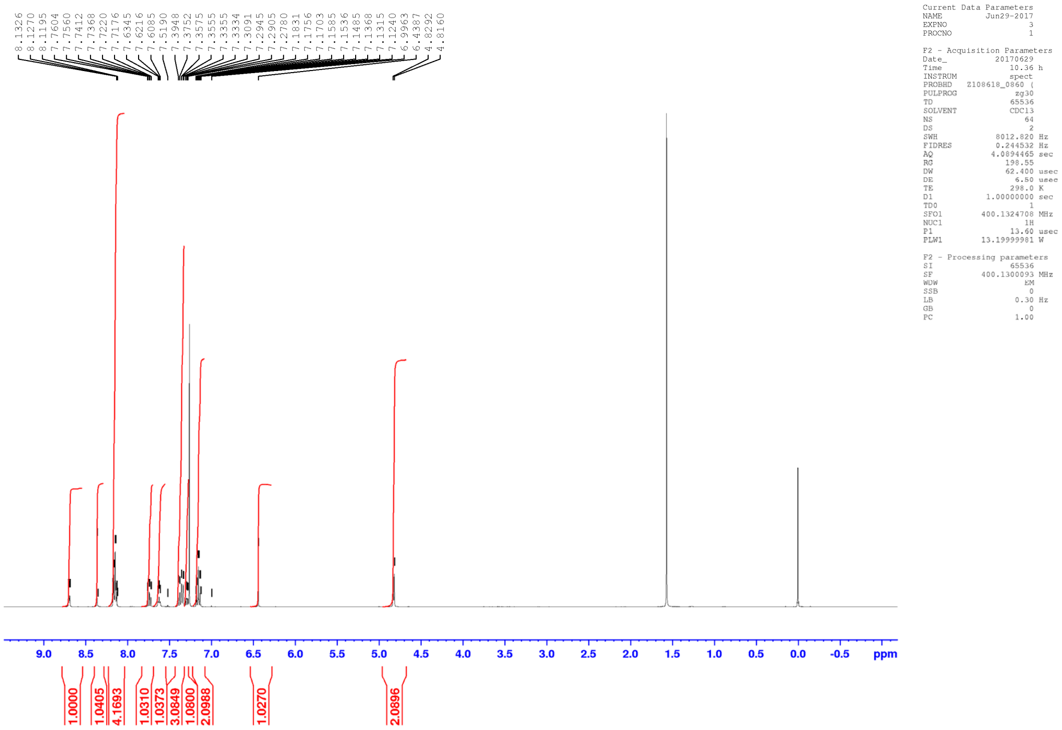
8.1326
8.1270
8.1195
7.7604
7.7560
7.7412
7.7368
7.7220
7.7176
7.6345
7.6216
7.6085
7.5190
7.3948
7.3752
7.3575
7.3555
7.3355
7.3334
7.3091
7.2945
7.2905
7.2780
7.1831
7.1756
7.1703
7.1585
7.1536
7.1485
7.1368
7.1315
7.1240
6.9963
6.4387
4.8292
4.8160
Current Data Parameters
NAME Jun29-2017
EXPNO 3
PROCNO 1
F2 - Acquisition Parameters
Date_ 20170629
Time 10.36 h
INSTRUM spect
PROBHD Z108618_0860 (
PULPROG zg30
TD 65536
SOLVENT CDCl3
NS 64
DS 2
SWH 8012.820 Hz
FIDRES 0.244532 Hz
AQ 4.0894465 sec
RG 198.55
DW 62.400 usec
DE 6.50 usec
TE 298.0 K
D1 1.00000000 sec
TD0 1
SFO1 400.1324708 MHz
NUC1 1H
P1 13.60 usec
PLW1 13.19999981 W
F2 - Processing parameters
SI 65536
SF 400.1300093 MHz
WDW EM
SSB 0
LB 0.30 Hz
GB 0
PC 1.00
9.0
8.5
8.0
7.5
7.0
6.5
6.0
5.5
5.0
4.5
4.0
3.5
3.0
2.5
2.0
1.5
1.0
0.5
0.0
-0.5
ppm
1.0000
1.0405
4.1693
1.0310
1.0373
3.0849
1.0800
2.0988
1.0270
2.0896

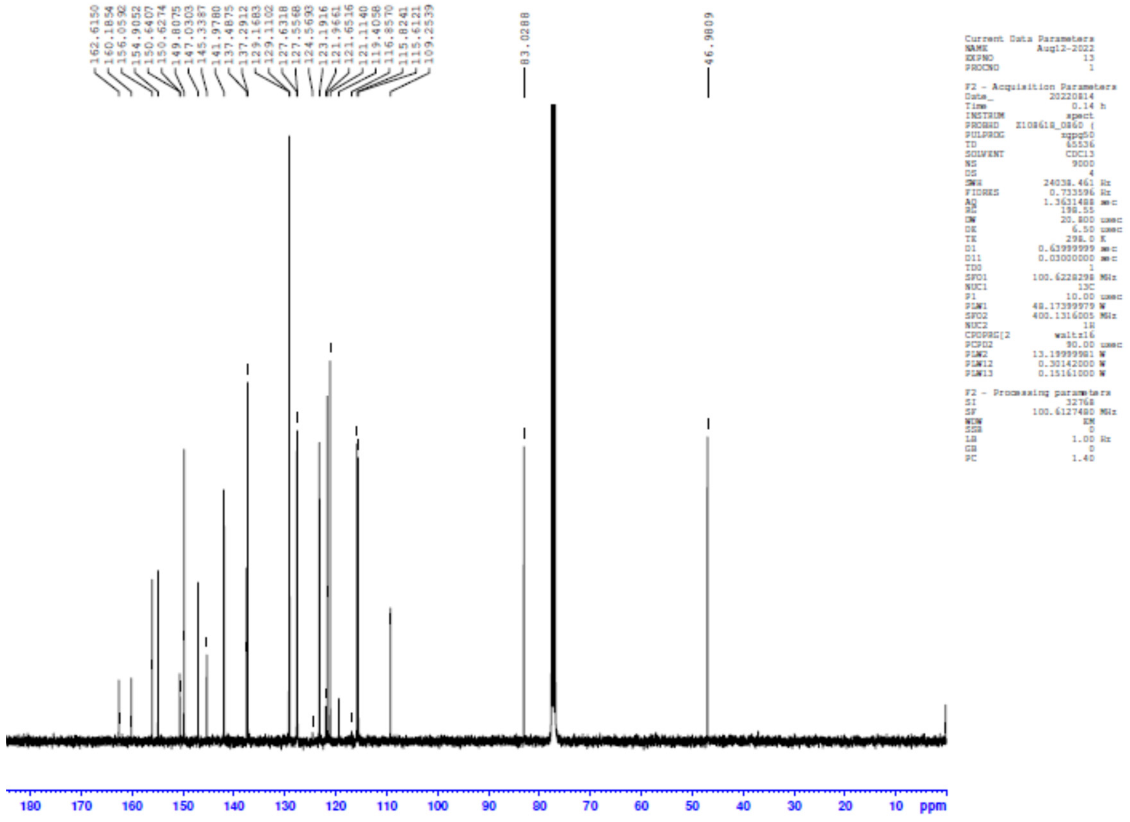
162.6150
160.1854
156.0592
154.9052
150.6407
150.6274
149.8075
147.0303
145.3387
141.9780
137.4875
137.2912
129.1683
129.1102
127.6318
127.5568
124.5693
123.1916
121.9461
121.6516
121.1140
119.4058
116.8570
115.8241
115.6121
109.2539
83.0288
46.9809
Current Data Parameters
NAME Aug12-2022
EXPNO 13
PROCNO 1
F2 - Acquisition Parameters
Date_ 20220814
Time 0.14 h
INSTRUM spect
PROBHD Z108618_0860 (
PULPROG zgpg30
TD 65536
SOLVENT CDCl3
NS 9200
DS 4
SWH 24038.461 Hz
FIDRES 0.733596 Hz
AQ 1.3631488 sec
RG 198.55
DW 20.800 usec
DE 6.50 usec
TE 298.0 K
D1 0.63999999 sec
D11 0.03000000 sec
TD0 1
SFO1 100.6228298 MHz
NUC1 13C
P1 10.00 usec
PLW1 48.17399979 W
SFO2 400.1316005 MHz
NUC2 1H
CPDPRG[2 waltz16
PCPD2 90.00 usec
PLW2 13.19999981 W
PLW12 0.30142000 W
PLW13 0.15161000 W
F2 - Processing parameters
SI 32768
SF 100.6127480 MHz
WDW EM
SSB 0
LB 1.00 Hz
GB 0
PC 1.40
180
170
160
150
140
130
120
110
100
90
80
70
60
50
40
30
20
10
ppm

Compound **39**

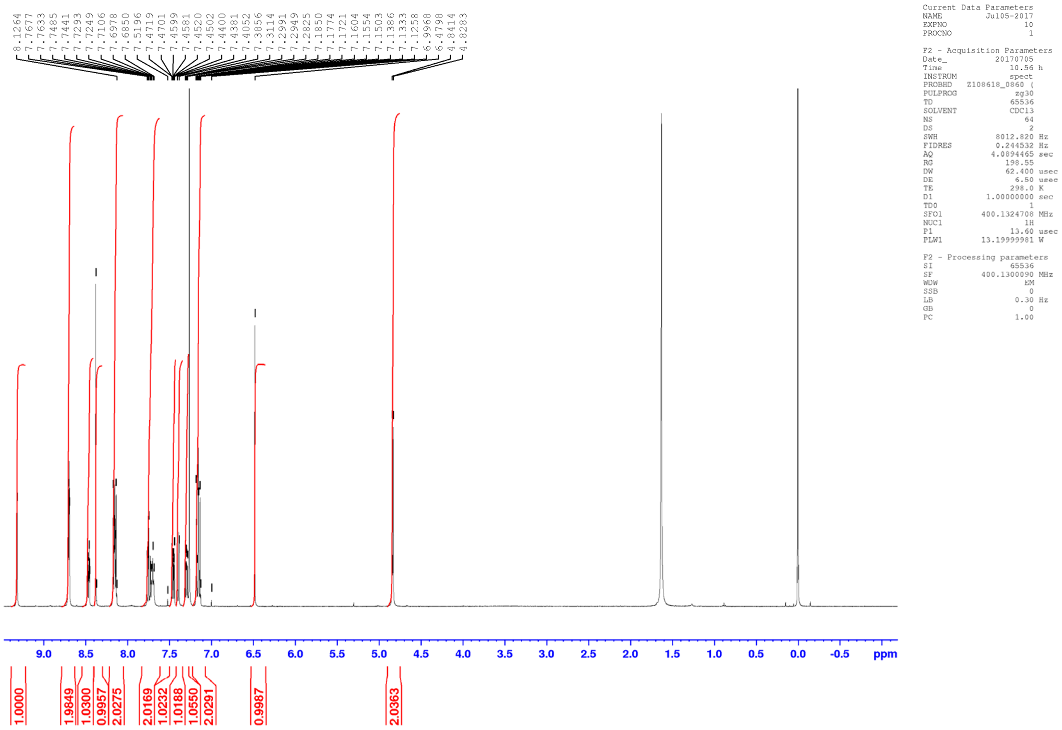
Current Data Parameters
NAME Jul05-2017
EXPNO 10
PROCNO 1
F2 - Acquisition Parameters
Date_ 20170705
Time 10.56 h
INSTRUM spect
PULPROG zg30
TD 65536
SOLVENT CDCl3
NS 64
DS 2
SWH 8012.820 Hz
FIDRES 0.244532 Hz
AQ 4.0894465 sec
RG 198.55
DW 62.400 usec
DE 6.50 usec
TE 298.0 K
D1 1.00000000 sec
TD0 1
SFO1 400.1324708 MHz
NUC1 1H
P1 13.60 usec
PLW1 13.19999981 W
F2 - Processing parameters
SI 65536
SF 400.1300090 MHz
WDW EM
SSB 0
LB 0.30 Hz
GB 0
PC 1.00
ppm

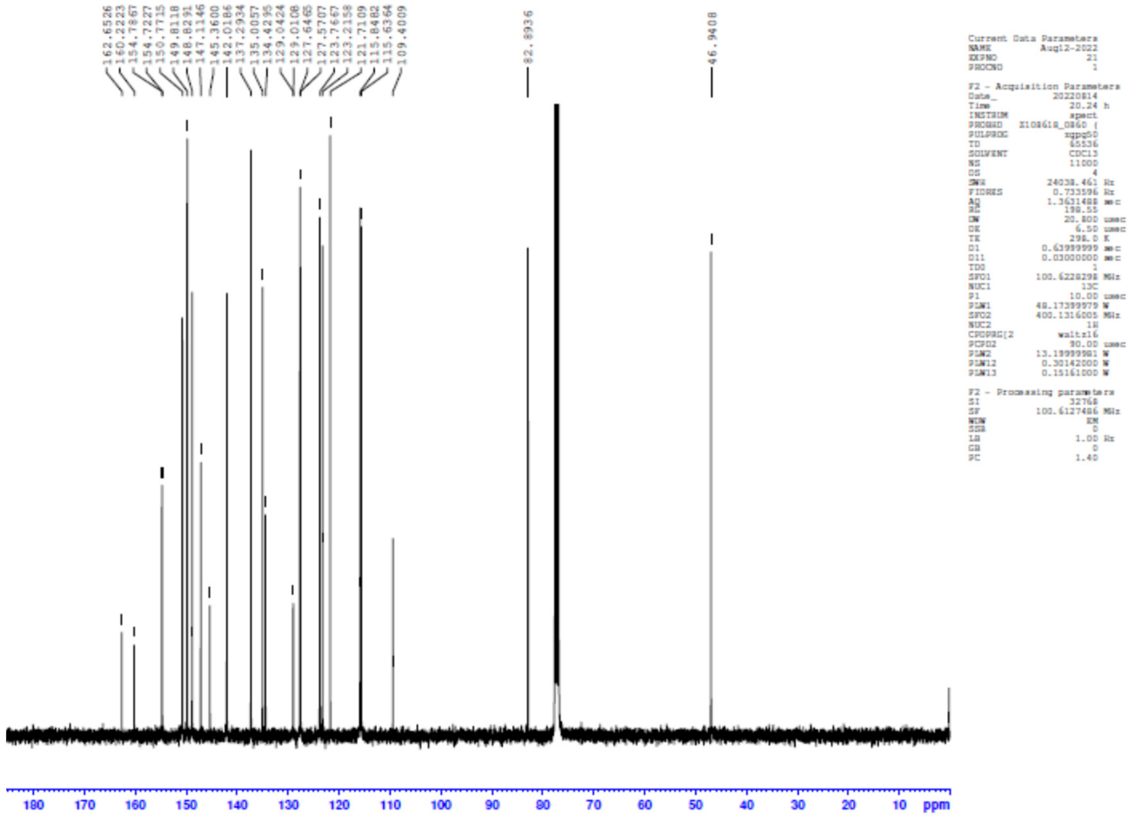
162.6526
160.2223
154.7867
154.7227
150.7715
149.8118
148.8291
147.1146
145.3600
142.0186
137.2934
135.0057
134.4235
129.0424
128.0108
127.6465
123.5707
123.7767
122.2158
121.7109
115.8482
115.6364
109.4009
82.8936
46.9408
ppm

Compound **41**

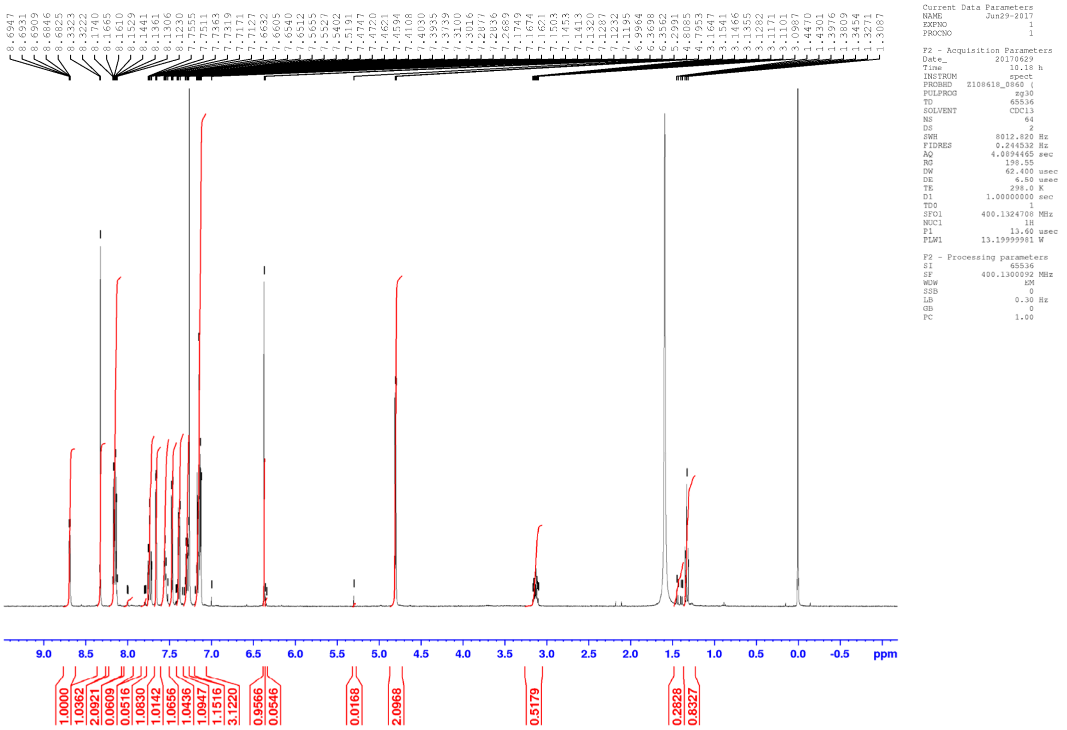

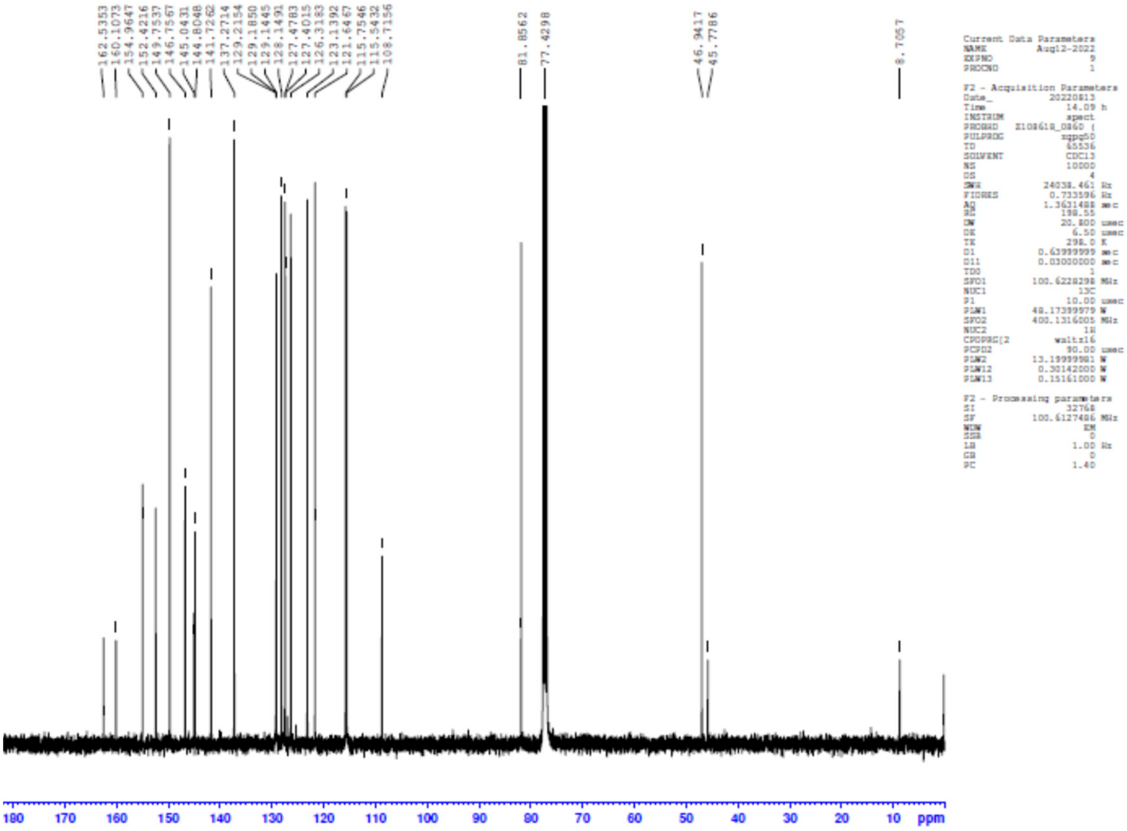

Compound **42**

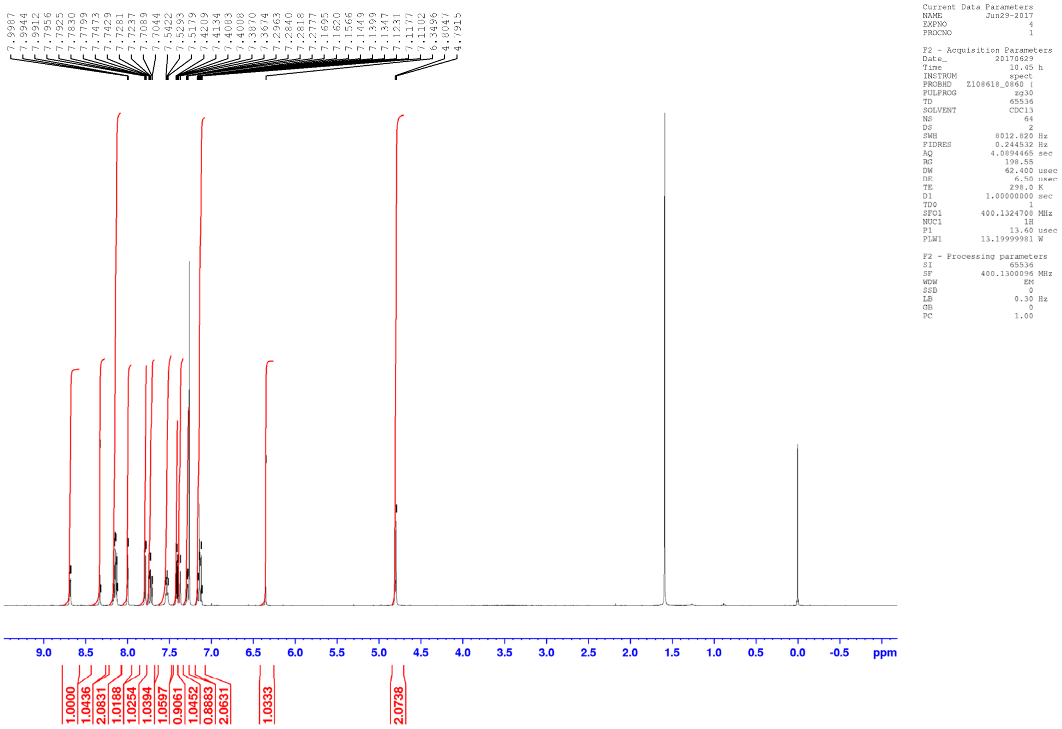

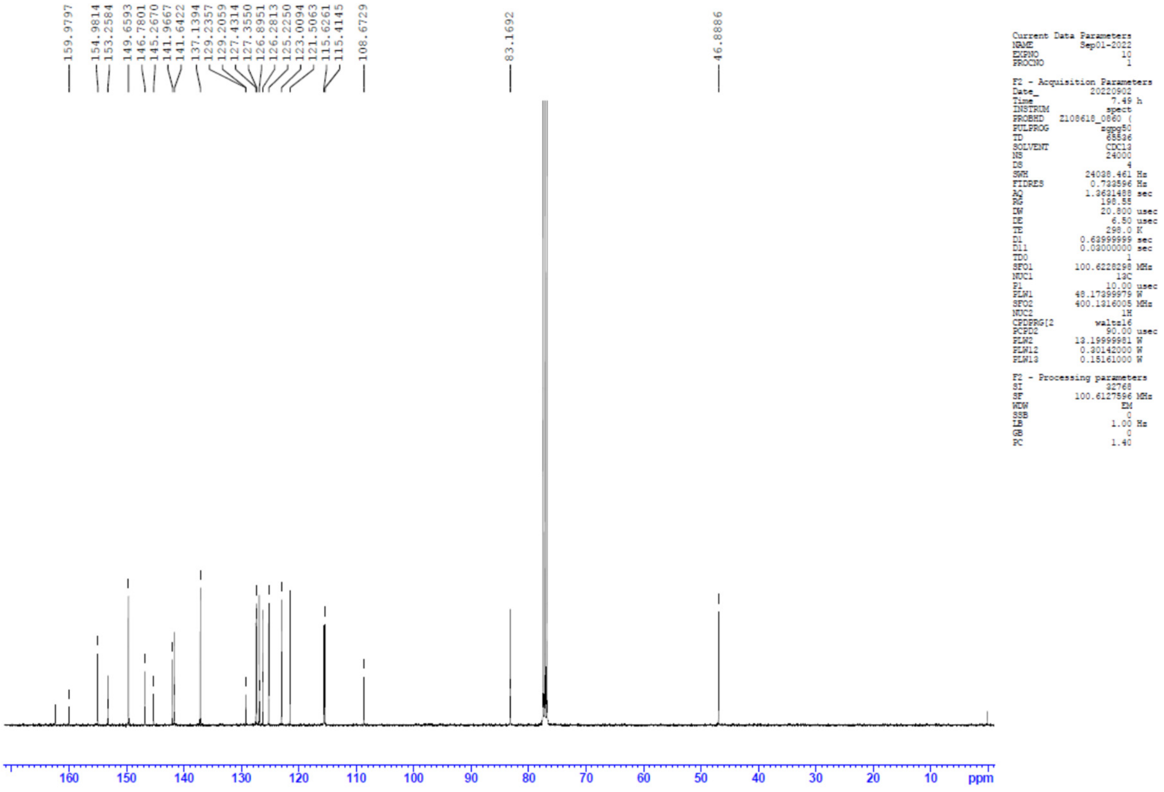

Compound **44**

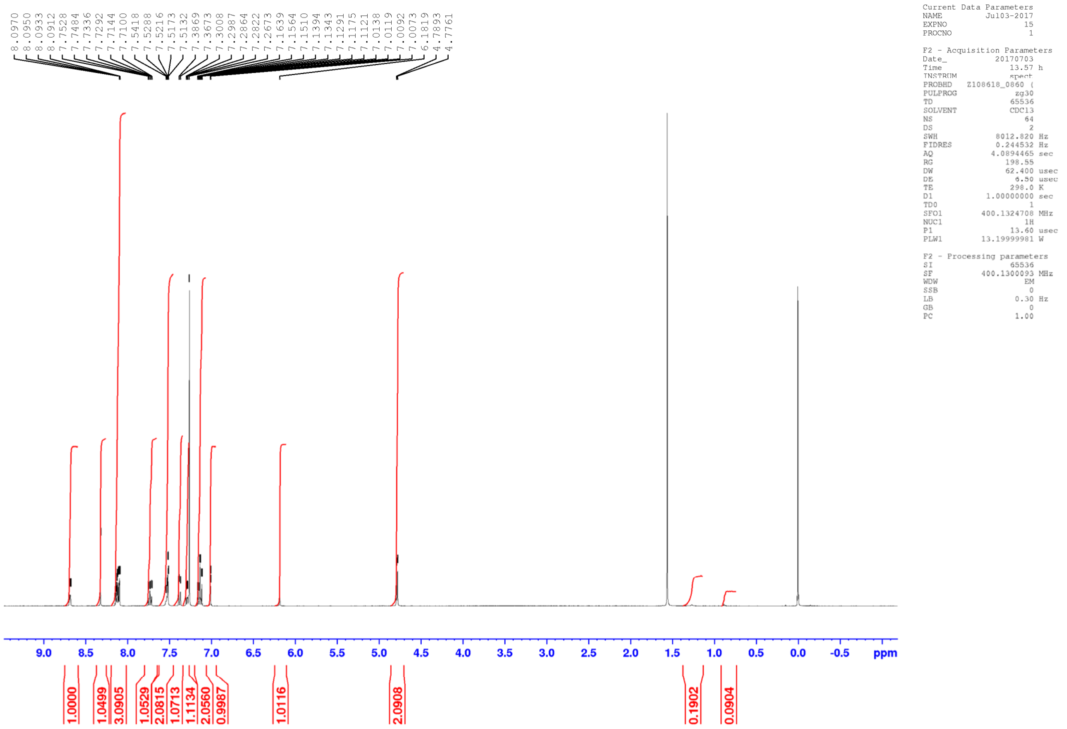

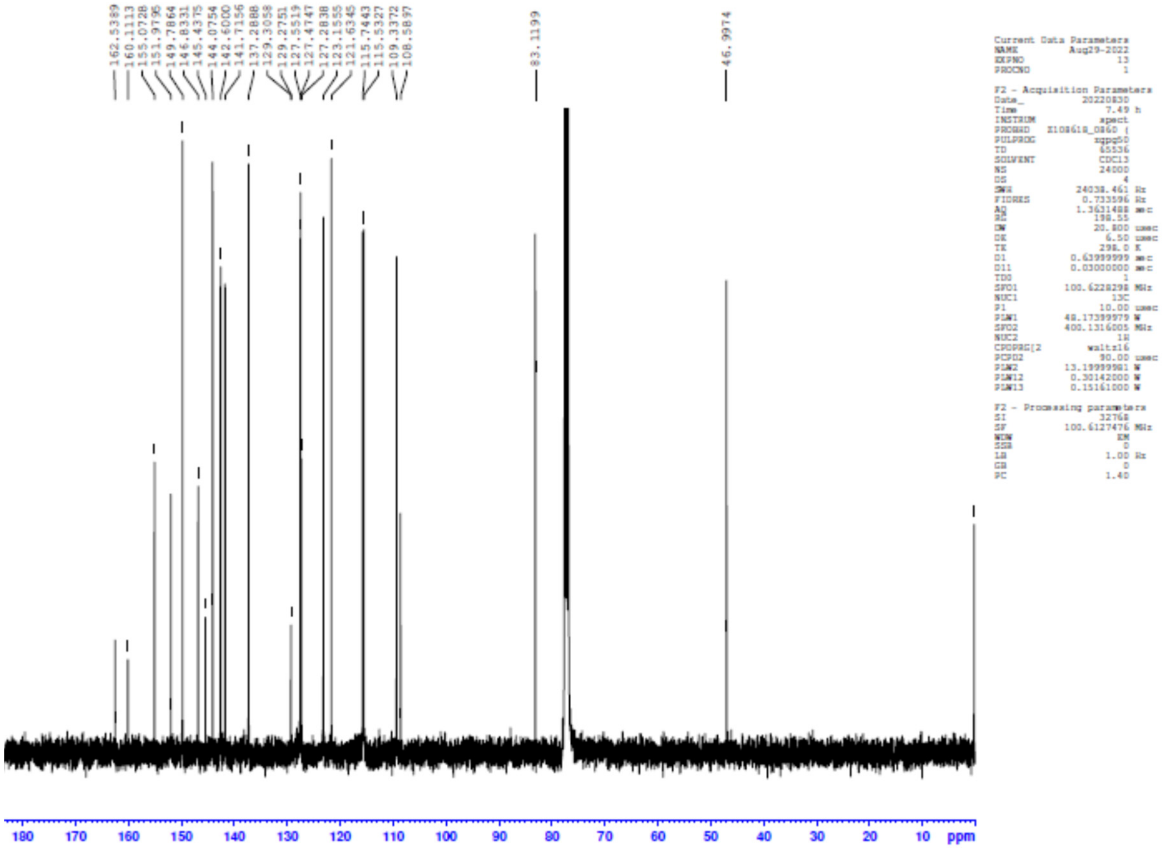

Compound **52**

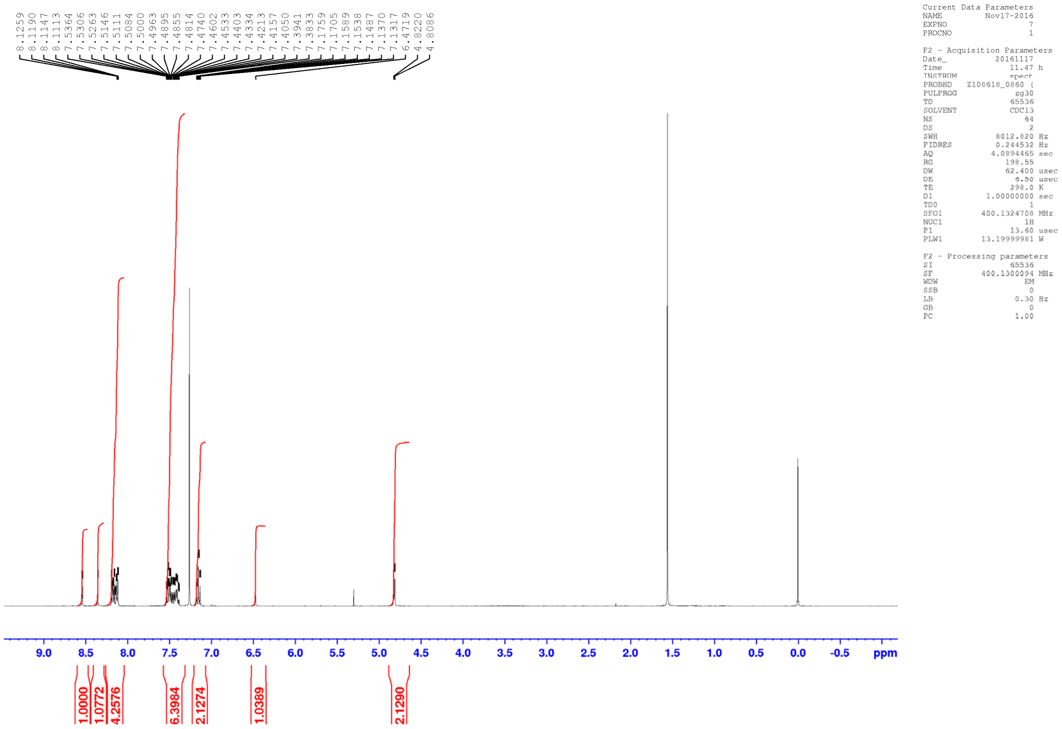

8.1259
8.1190
8.1147
8.1113
7.5364
7.5306
7.5263
7.5146
7.5111
7.5084
7.5000
7.4963
7.4895
7.4855
7.4814
7.4740
7.4602
7.4533
7.4403
7.4334
7.4213
7.4157
7.4050
7.3941
7.3833
7.1759
7.1705
7.1589
7.1538
7.1487
7.1370
7.1317
6.4719
4.8220
4.8086
Current Data Parameters
NAME Nov17-2016
EXPNO 7
PROCNO 1
F2 - Acquisition Parameters
Date_ 20161117
Time 11.47 h
INSTRUM spect
PROBHD Z108618_0860 (
PULPROG zg30
TD 65536
SOLVENT CDCl3
NS 64
DS 2
SWH 8012.820 Hz
FIDRES 0.244532 Hz
AQ 4.0894465 sec
RG 198.55
DW 62.400 usec
DE 6.50 usec
TE 298.0 K
D1 1.00000000 sec
TD0 1
SFO1 400.1324708 MHz
NUC1 1H
P1 13.60 usec
PLW1 13.19999981 W
F2 - Processing parameters
SI 65536
SF 400.1300094 MHz
WDW EM
SSB 0
LB 0.30 Hz
GB 0
PC 1.00
9.0
8.5
8.0
7.5
7.0
6.5
6.0
5.5
5.0
4.5
4.0
3.5
3.0
2.5
2.0
1.5
1.0
0.5
0.0
-0.5
ppm
1.0000
1.0772
4.2576
6.3984
2.1274
1.0389
2.1290

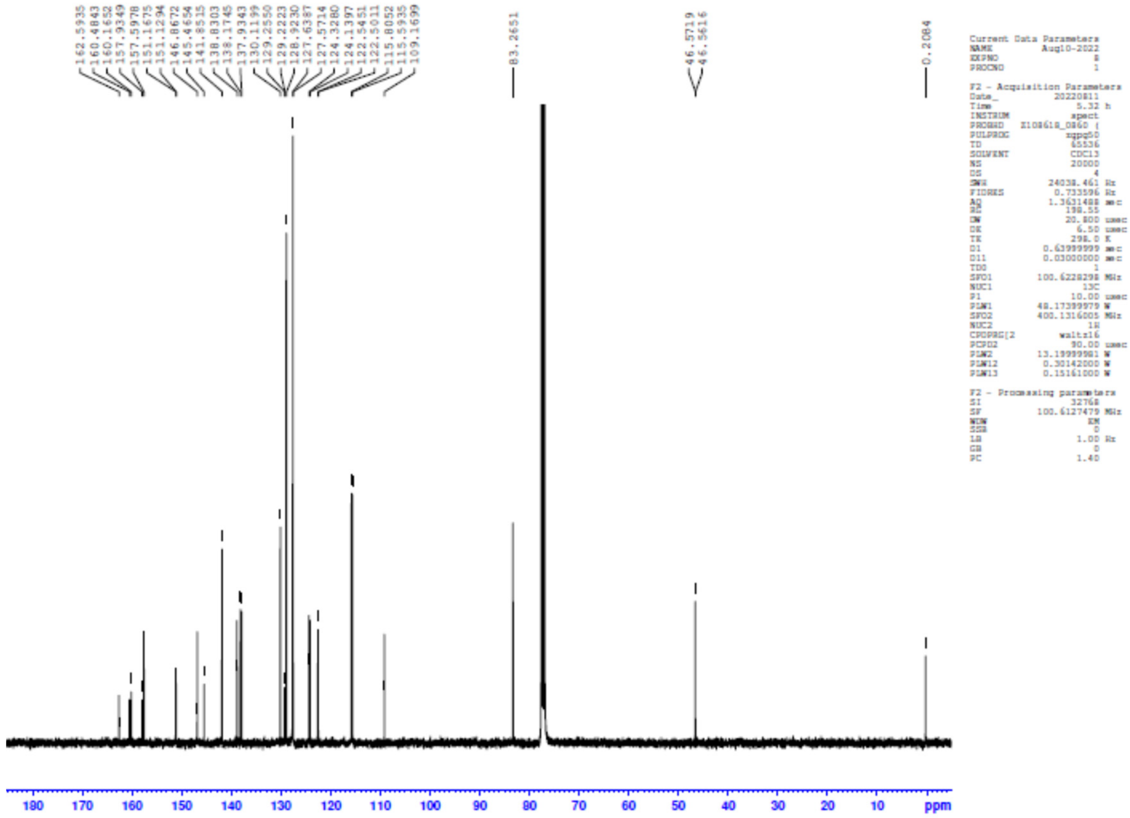

162.5935
160.4843
160.1652
157.9349
157.5978
151.1675
151.1294
146.8672
145.4654
141.8535
138.8303
138.1745
137.9343
130.1199
129.2550
129.2223
128.9230
128.6387
127.5714
124.3280
124.1397
122.5511
122.5011
115.8052
115.5935
109.1699
83.2651
46.5719
46.5616
0.2084
Current Data Parameters
NAME Aug10-2022
EXPNO 8
PROCNO 1
F2 - Acquisition Parameters
Date_ 20220811
Time 5.32 h
INSTRUM spect
PROBHD Z108618_0860 (
PULPROG zgpg30
TD 65536
SOLVENT CDCl3
NS 20000
DS 4
SWH 24038.461 Hz
FIDRES 0.733596 Hz
AQ 1.3631488 sec
RG 198.55
DW 20.800 usec
DE 6.50 usec
TE 298.0 K
D1 0.63999999 sec
D11 0.03000000 sec
TD0 1
SFO1 100.6228298 MHz
NUC1 13C
P1 10.00 usec
PLW1 48.17399979 W
SFO2 400.1316005 MHz
NUC2 1H
CPDPRG[2 waltz16
PCPD2 90.00 usec
PLW2 13.19999981 W
PLW12 0.30142000 W
PLW13 0.15161000 W
F2 - Processing parameters
SI 32768
SF 100.6127479 MHz
WDW EM
SSB 0
LB 1.00 Hz
GB 0
PC 1.40
180
170
160
150
140
130
120
110
100
90
80
70
60
50
40
30
20
10
ppm

Compound **53**

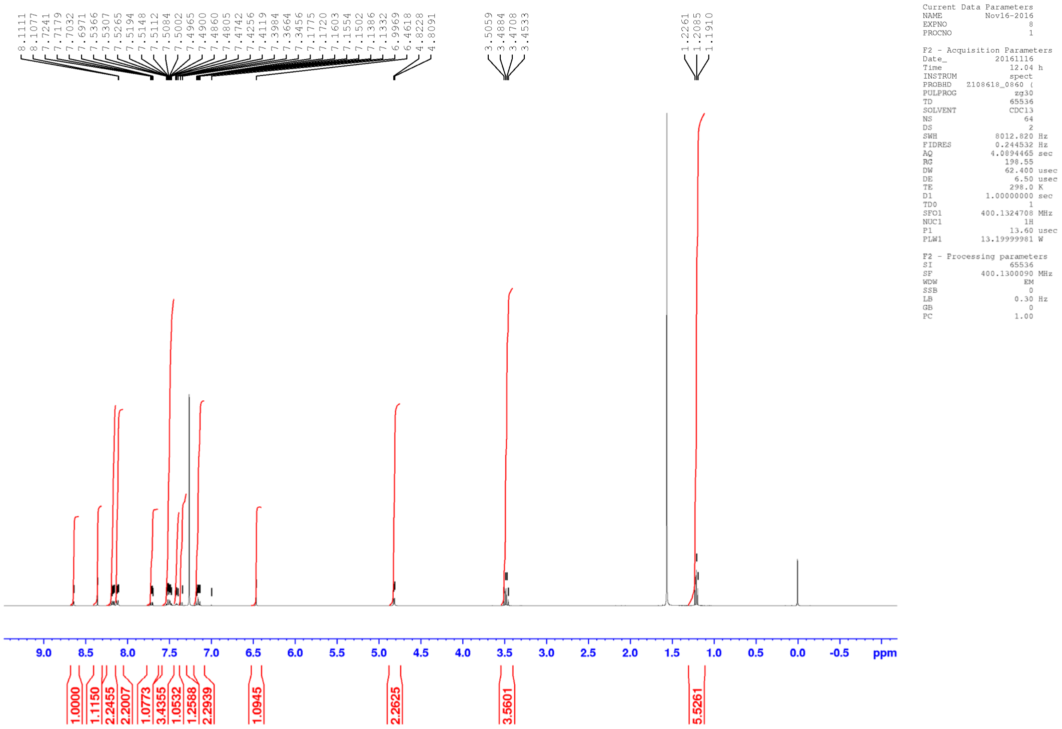

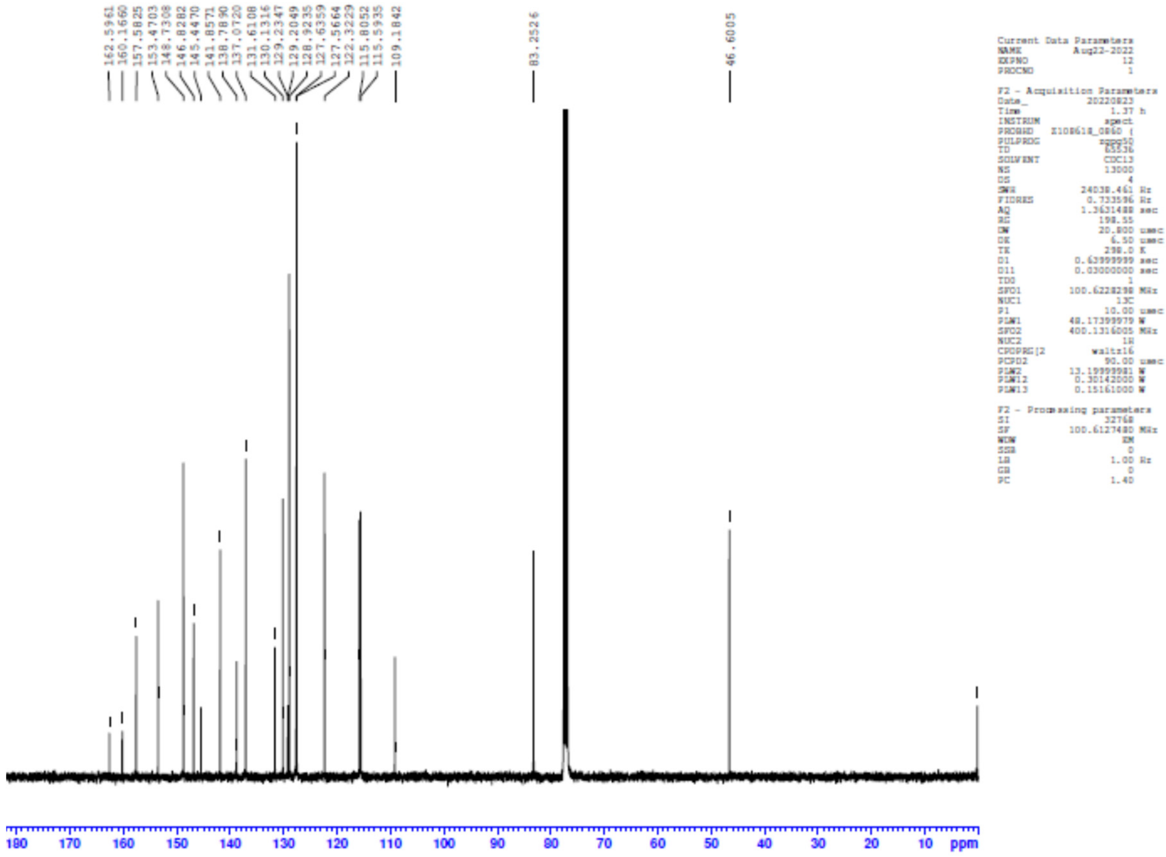

Compound **54**

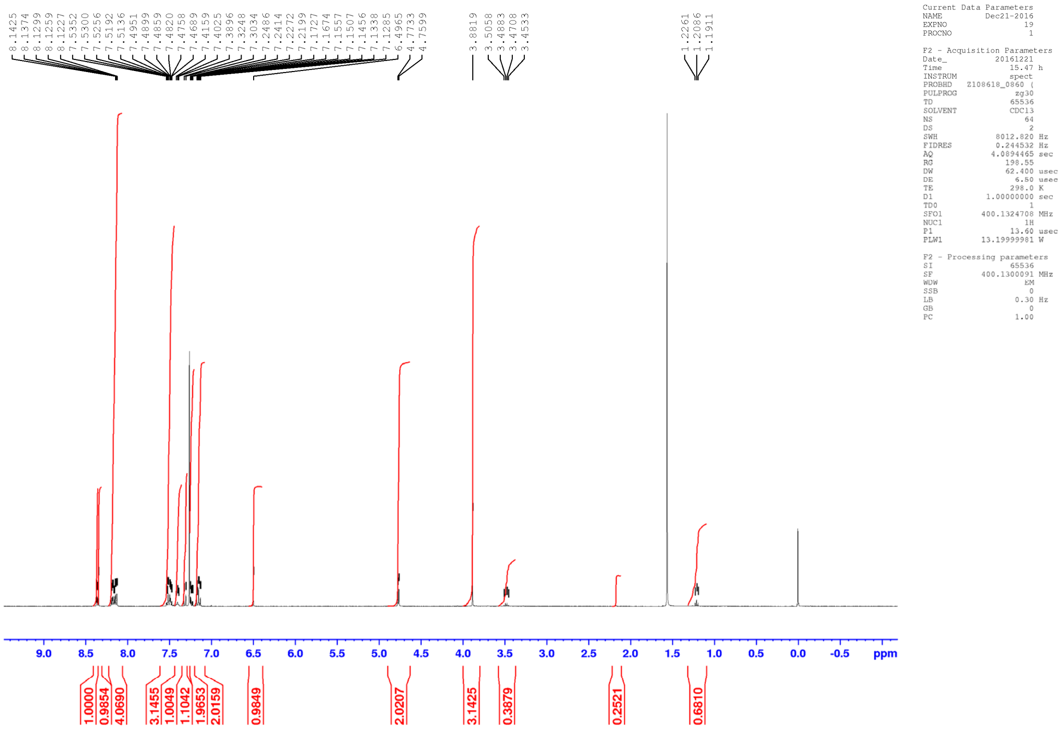

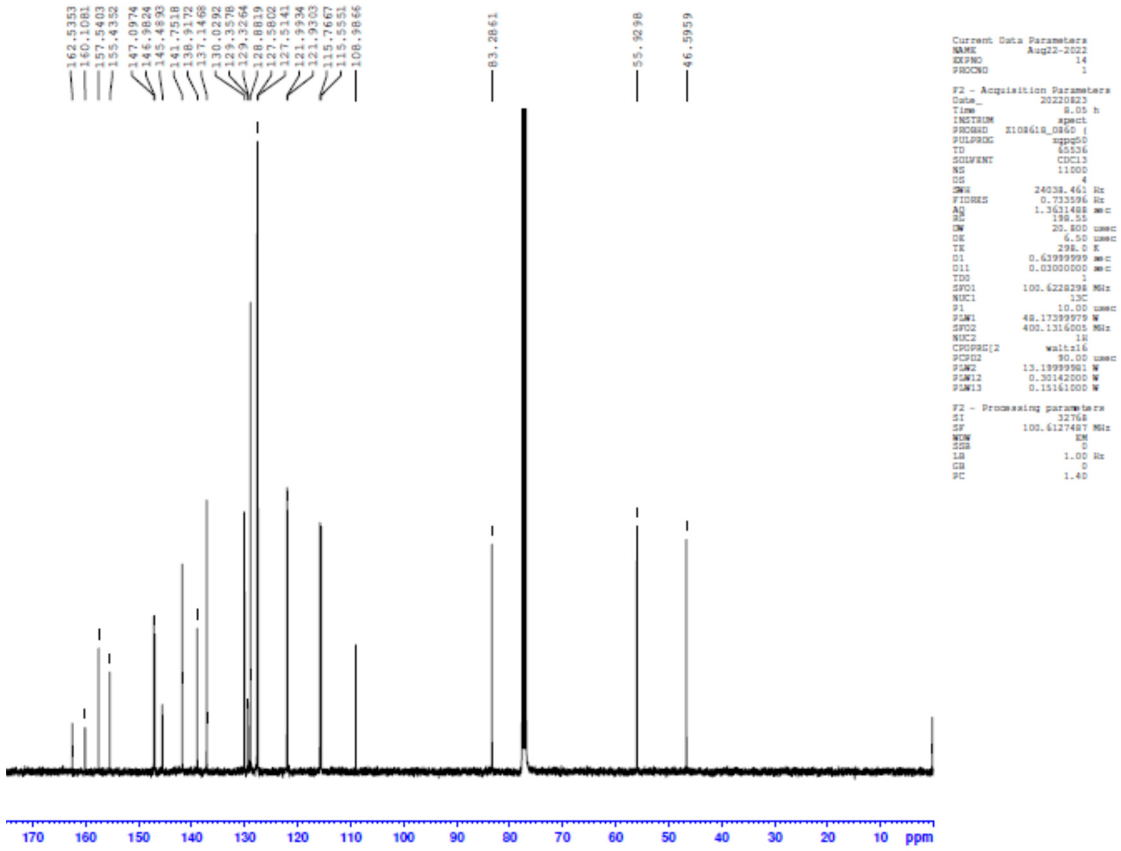

Compound **55**

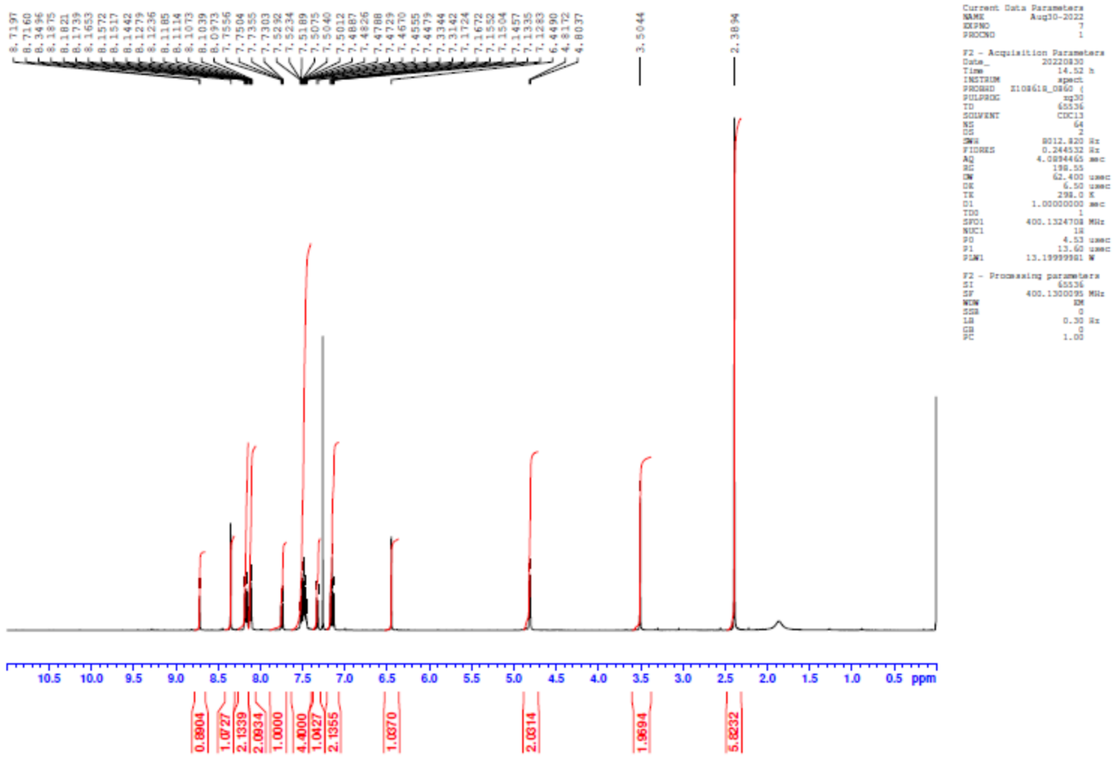

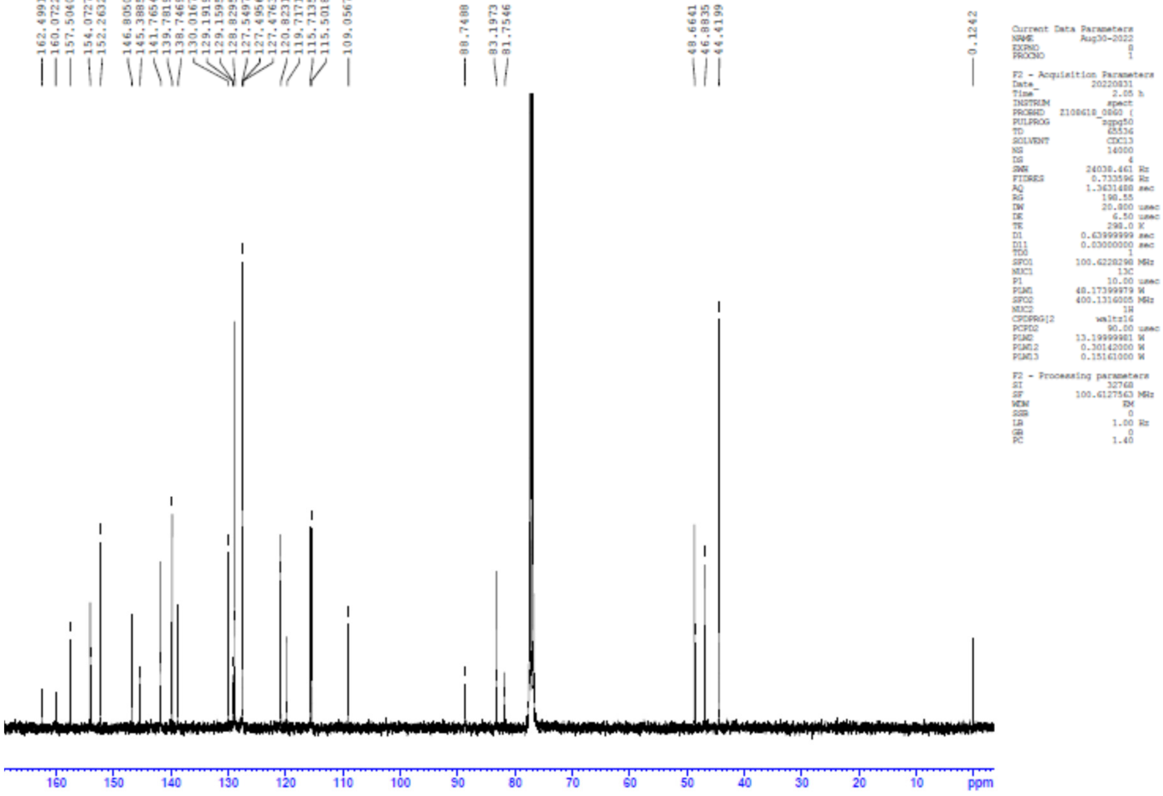

Compound **62**

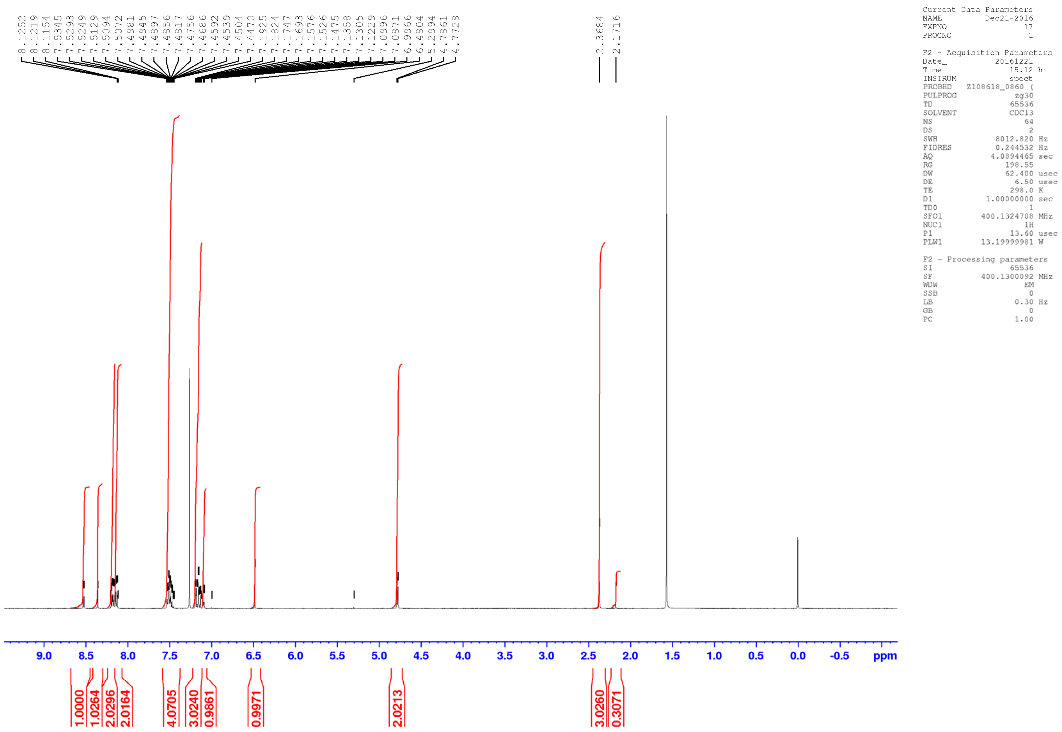

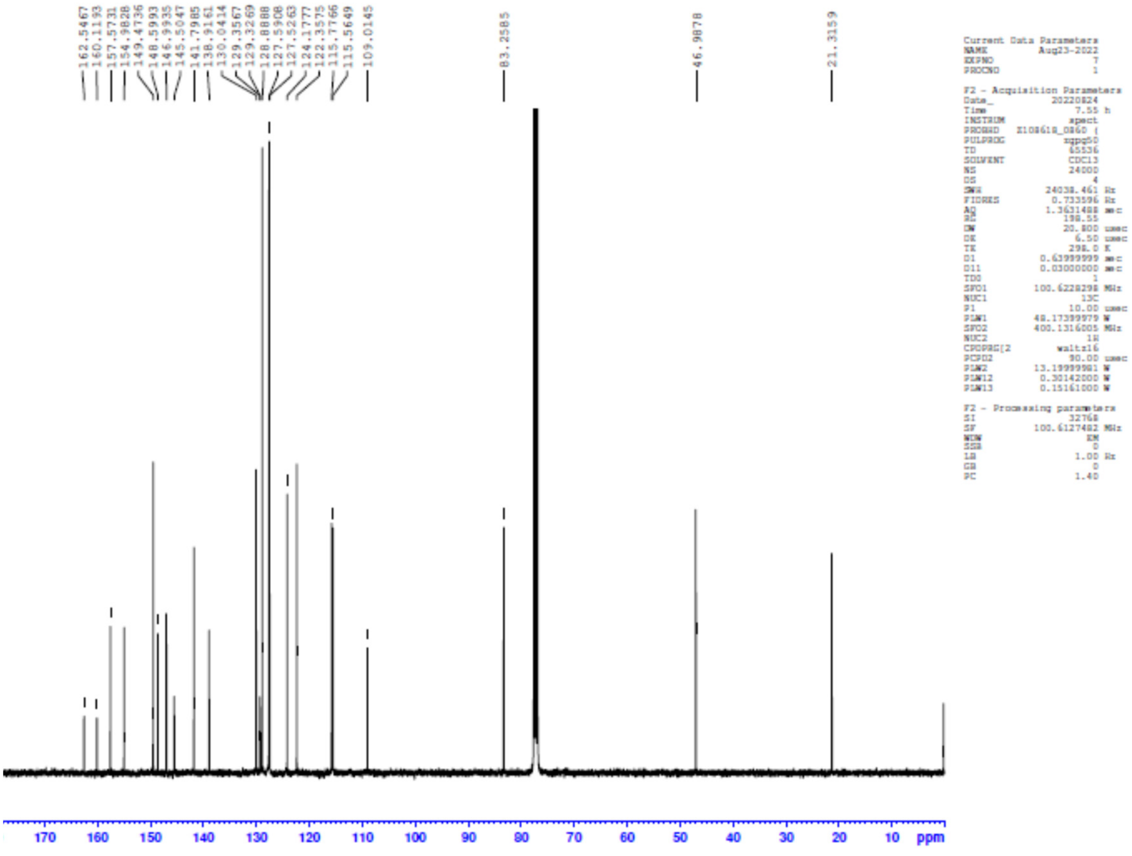

Compound **63**

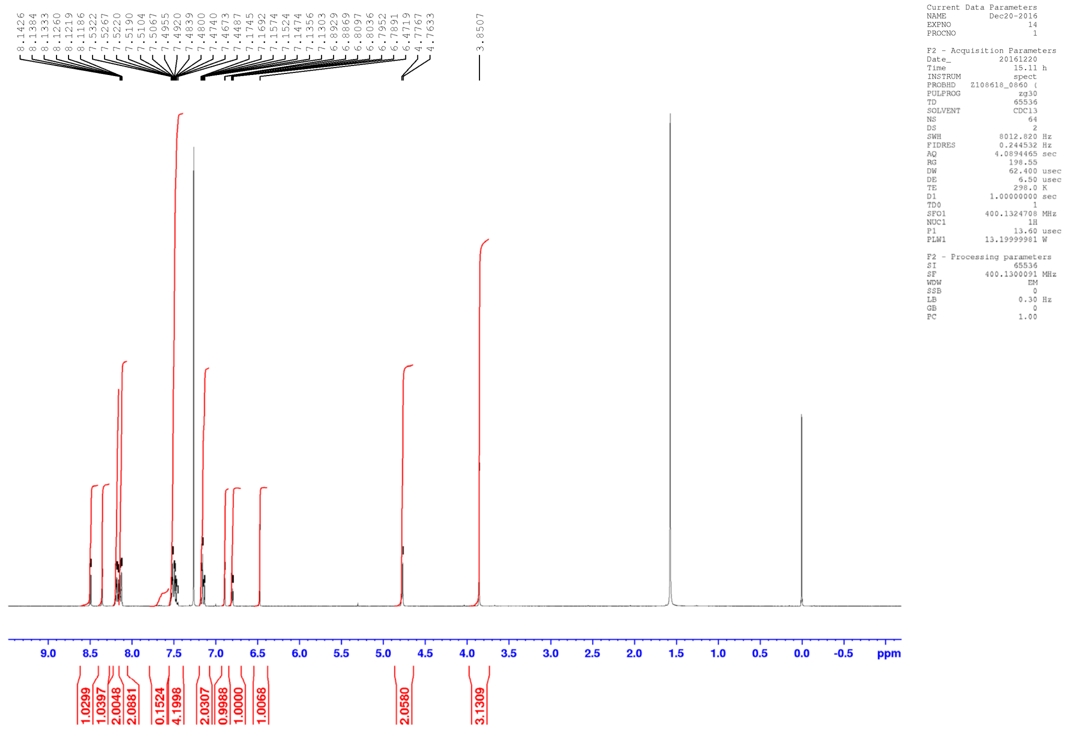

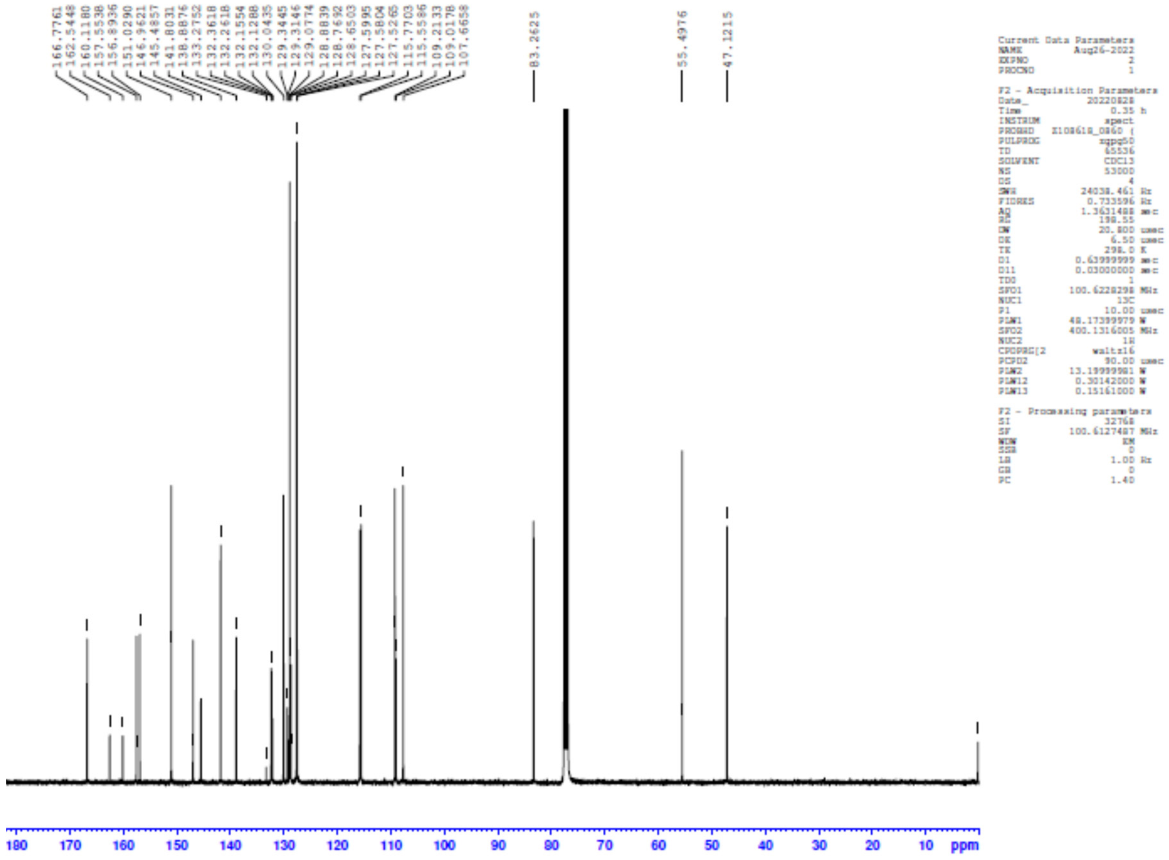

Compound **68**

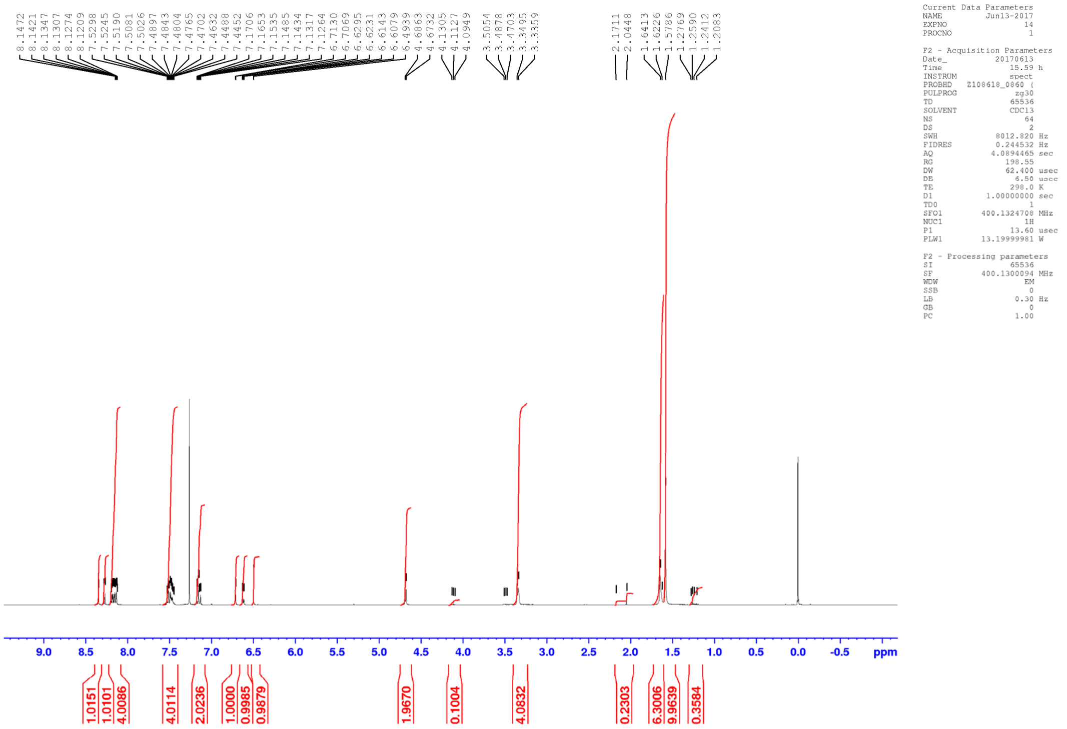

8.1472
8.1421
8.1347
8.1307
8.1274
8.1209
7.5298
7.5245
7.5190
7.5081
7.5026
7.4897
7.4843
7.4804
7.4765
7.4702
7.4632
7.4488
7.4452
7.1706
7.1653
7.1535
7.1485
7.1434
7.1317
7.1264
6.7130
6.7069
6.6295
6.6231
6.6143
6.6079
6.4939
4.6863
4.6732
4.1305
4.1127
4.0949
3.5054
3.4878
3.4703
3.3495
3.3359
2.1711
2.0448
1.6413
1.6226
1.5786
1.2769
1.2590
1.2412
1.2083
9.0 8.5 8.0 7.5 7.0 6.5 6.0 5.5 5.0 4.5 4.0 3.5 3.0 2.5 2.0 1.5 1.0 0.5 0.0 -0.5 ppm
1.0151
1.0101
4.0086
4.0114
2.0236
1.0000
0.9985
0.9879
1.9670
0.1004
4.0832
0.2303
6.3006
9.9639
0.3584
Current Data Parameters
NAME Jun13-2017
EXPNO 14
PROCNO 1
F2 - Acquisition Parameters
Date_ 20170613
Time 15.59 h
INSTRUM spect
PROBHD Z108618_0860 (
PULPROG zg30
TD 65536
SOLVENT CDCl3
NS 64
DS 2
SWH 8012.820 Hz
FIDRES 0.244532 Hz
AQ 4.0894465 sec
RG 198.55
DW 62.400 usec
DE 6.60 usec
TE 298.0 K
D1 1.00000000 sec
TD0 1
SFO1 400.1324708 MHz
NUC1 1H
P1 13.60 usec
PLW1 13.19999981 W
F2 - Processing parameters
SI 65536
SF 400.1300094 MHz
WDW EM
SSB 0
LB 0.30 Hz
GB 0
PC 1.00

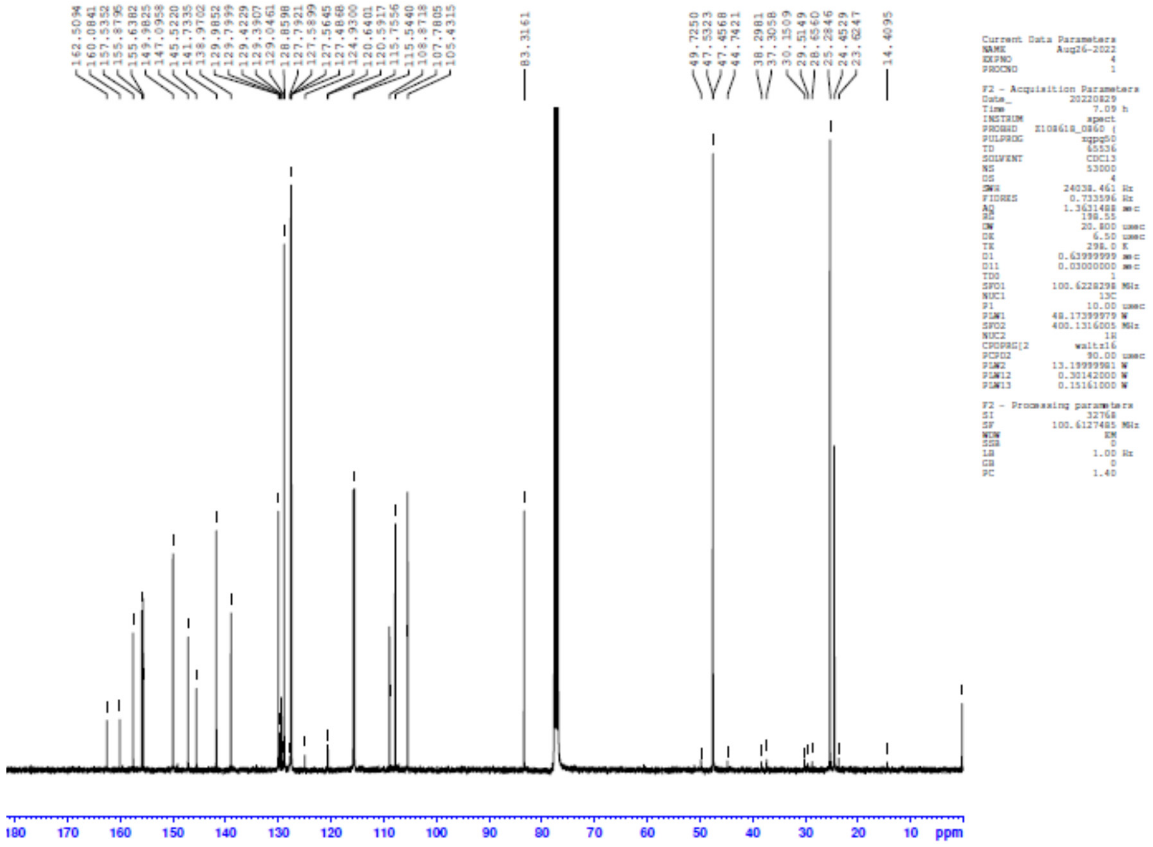

162.5094
160.0841
157.5352
155.8796
155.6382
149.9825
147.0958
145.5220
141.7335
138.9702
129.9852
129.7999
129.4229
129.3907
129.0461
128.8598
127.7921
127.5899
127.5645
127.4868
124.9300
120.6401
120.5917
115.7556
115.5440
108.8718
107.7805
105.4315
83.3161
49.7250
47.5323
47.4568
44.7421
38.2981
37.3058
30.1509
29.5149
28.6560
25.2846
24.4529
23.6247
14.4095
180 170 160 150 140 130 120 110 100 90 80 70 60 50 40 30 20 10 ppm
Current Data Parameters
NAME Aug26-2022
EXPNO 4
PROCNO 1
F2 - Acquisition Parameters
Date_ 20220829
Time 7.09 h
INSTRUM spect
PROBHD Z108618_0860 (
PULPROG zgpg30
TD 65536
SOLVENT CDCl3
NS 53000
DS 4
SWH 24038.461 Hz
FIDRES 0.733596 Hz
AQ 1.3631488 sec
RG 198.55
DW 20.800 usec
DE 6.50 usec
TE 298.0 K
D1 0.63999999 sec
D11 0.03000000 sec
TD0 1
SFO1 100.6228298 MHz
NUC1 13C
P1 10.00 usec
PLW1 48.17399979 W
SFO2 400.1316005 MHz
NUC2 1H
CPDPRG[2 waltz16
PCPD2 90.00 usec
PLW2 13.19999981 W
PLW12 0.30142000 W
PLW13 0.15161000 W
F2 - Processing parameters
SI 32768
SF 100.6127485 MHz
WDW EM
SSB 0
LB 1.00 Hz
GB 0
PC 1.40
